# Meta-analysis and systematic review of peripheral platelet-associated biomarkers to explore the pathophysiology of alzheimer's disease

**DOI:** 10.1186/s12883-023-03099-5

**Published:** 2023-02-11

**Authors:** Jiajia Fu, Xiaohui Lai, Yan Huang, Ting Bao, Jing Yang, Sihui Chen, Xueping Chen, Huifang Shang

**Affiliations:** 1 Department of Neurology, West China Hospital, Sichuan University, Chengdu, Sichuan China; 2 Management Center, West China Hospital, Sichuan University, Chengdu, Sichuan China

**Keywords:** Alzheimer's disease, Platelets, Meta-analysis, Systematic review

## Abstract

**Introduction:**

Platelets are the primary peripheral reserve of amyloid precursor protein (APP), providing more than 90% of blood amyloid-beta (Aβ). Some oxidative stress markers and neurotransmitter markers were also differentially expressed in the peripheral platelets of AD. Therefore, the present study explored the differences in platelet-associated biomarkers between AD and healthy controls using meta-analysis and systematic review to reveal the value of platelet in the pathogenesis and development of AD.

**Methods:**

We searched all the related studies that probed into the platelets in AD based on PubMed, Embase, and web of science databases from the establishment to November 04, 2021.

**Results:**

Eighty-eight studies were included in the meta-analysis, and the platelets data of 702 AD and 710 controls were analyzed. The results of standardized mean difference (SMD) showed that platelets in AD had lower levels of APP ratio (SMD: -1.89; *p* < 0.05), ADAM10 (SMD: -1.16; *p* < 0.05), Na + -K + -ATPase (SMD: -7.23; *p* < 0.05), but higher levels of HMW/LMW tau (SMD: 0.92; *p* < 0.05), adenosine A_2_ receptor (SMD: 4.27; *p* < 0.05), MAO-B (SMD: 1.73; *p* < 0.05), NO (SMD: 4.25; *p* < 0.05) and ONOO^−^ (SMD: 7.33; *p* < 0.05). In the systematic review, some other platelet markers seem to be meaningful in AD patients.

**Conclusion:**

The results of the present meta-analysis and systematic review demonstrated that the alterations of APP metabolic enzymes, oxidative stress markers, and neurotransmitter factors in platelets were similar to their changes in the central nervous system of AD, suggesting that platelet could be a good source of peripheral biomarkers and may play an important role in the pathophysiological development of AD.

**Supplementary Information:**

The online version contains supplementary material available at 10.1186/s12883-023-03099-5.

## Introduction

Alzheimer's disease (AD) is an age-related progressive neurodegenerative disorder, leading to the progressive cognitive deterioration [1]. AD is the most common dementia, accounting for 60% to 70% of dementia. Alzheimer's Disease International (ADI) estimates that more than 50 million people worldwide are living with dementia, which will increase to 152 million by 2050. With the rapid aging of the global population, AD has become a major social problem, costing the world an estimated $1 trillion a year and placing a crushing burden on patients and their caregivers [2]. However, there is no effective treatment and no clear therapeutic targets in AD. Therefore, it is very important to clarify the pathophysiology of AD and explore more effective biomarkers.

The pathological features of AD are insoluble parenchymal deposits of amyloid-beta (Aβ) and tau tangle. Aβ42 is the main component of senile plaques in the brain of AD patients. The Aβ is a highly hydrophobic peptide of 39–43 amino acids, released by the proteolytic cleavage of amyloid-β precursor protein (APP) [3], which is hydrolyzed to Aβ via the amyloidosis pathway with a continuous enzymatic action of β- and γ- secretase [3]. APP is cleaved by α- and γ- secretase to prevent Aβ formation in the non-amyloidosis pathway. Beta-site APP-cleaving enzyme 1 (BACE1) has been reported to have β-secretase activity. A-disintegrin and metalloprotease 10 (ADAM10) is associated with α-secretase activity. Presenilin-1 (PSEN-1) is composed of one of the four proteins of the γ-secretase complex. Studies have shown that both central and peripheral biomarkers of APP metabolism could provide important insights into the neurobiology of AD [4], some of which have diagnostic significance [5].

APP is a ubiquitous protein that is expressed in various cells, including platelets, white blood cells, and so on. Human platelets are the largest source of circulating APP, and the blood APP cleavage pathway is close to the amyloidosis and non-amyloidosis pathway in the central nervous system (CNS) [6]. Platelets contain the cellular mechanisms necessary for APP processing, and platelets are the preferential activation of amyloid workarounds in AD patients [4]. Full-length and processed APP exists in human platelets and has been shown to contain BACE1and release Aβ [4]. More recently, Tang et al. found increased levels of Aβ, increased immunoreactivity of BACE1, and decreased immunoreactivity of ADAM10 in platelets of AD, indicating that the amyloidogenic pathway of the APP metabolism is activated in platelets of AD patients, paralleling the intracerebral APP processing in AD [4]. However, Bermejo-Besco et al. found a reduction of APP levels and augmented levels of ADAM10 in AD patients [7]. Zainaghi et al. showed that changes in platelet APP fragments were associated with membrane fluidity and cognitive decline [6]. Zubenko et al. found that relatives of AD patients with increased platelet membrane fluidity developed dementia significantly earlier than those with normal platelet membrane fluidity [8]. The fluorescence anisotropy of 1,6-diphenyl-1,3,5-hexatriene (DPH) in platelet membranes was found to be significantly reduced in AD patients [8], but other studies found the opposite result [9].

Oxidative stress (OS) is a state of imbalance between free radical production and free radical degradation by the antioxidant system, and OS is considered to be a key factor in the pathogenesis of AD and mild cognitive impairment (MCI) [10]. OS and Aβ production are proportionally related; Aβ could induce oxidative stress in vivo and in vitro, while OS could increase Aβ production through proteolytic APP. Butterfield et al. experimentally verified that Aβ-associated free radical damage is a fundamental process of AD [10]. Studies also found that OS in platelets increased with aging, and was more prominent in AD patients [11]. Some studies found that NO production in platelets of AD was significantly increased [9], but the results were not consistent [12].

Although the neurobiological background of AD is characterized primarily by the accumulation of amyloid plaques and tangles in the brain, changes in other neurotransmitter systems are also contributing to cognitive deficits and behavioral disorders in AD patients [13]. Some intracellular signaling pathways important for platelet activation were also being described as regulating APP processing, including the synthesis and release of neurotransmitters (serotonin, glutamate, dopamine, etc.) [14]. Genetic studies suggest the role of serotonin (5-hydroxytryptamine, 5-HT) 2A and 5-HT 2C receptor polymorphisms in developing AD behavior and psychological symptoms. Platelets are considered to be an easily available peripheral model for studying the presynaptic (5-HT reuptake, monoamine oxidase B/MAO-B/ activity) and postsynaptic (5-HT 2A receptor binding) processes in central 5-hydroxyaminergic neurons [15]. However, current findings on platelet 5-HT concentration and platelet MAO-B activity in AD patients are inconsistent.

The abnormalities in APP metabolism, APP secretases, OS, and other intracellular signaling pathway biomarkers have been demonstrated in platelets of AD, but the results were conflicting. In the present study, we analyzed the differences of platelet-associated metabolic markers between AD patients and normal controls through meta-analysis and systematic review, exploring the related roles of platelets in the development and pathogenesis of AD.

## Methods

### Search strategy and selection criteria

The two investigators conducted systematic document retrieval. Original studies reporting measures of platelet-associated markers in peripheral blood were searched through PubMed, Embase, and Web of Science from the establishment to November 04, 2021. Mesh terms and topic terms were used as the searching term, including "platelet" and "Alzheimer s disease", "Alzheimer Dementia", "Senile Dementia", "Alzheimer Type Dementia", "Alzheimer Sclerosis", "Presenile Dementia." (supplementary material 1) Any disagreements among investigators were resolved through negotiation and arbitration. To avoid missing literature, we also looked through the references of all the relevant articles.

English-language or Chinese-language publications reporting concentrations of platelet-associated markers in living human beings were included if they met the following criteria: (1) original studies reported data in at least two of the groups (AD and control); (2) the principles that these studies used to diagnose AD were qualified; (3)literature sources and necessary data were available and all included articles reported the platelet-associated markers data of the control group and AD patients, with mean value with standard derivation (SD); (4) The control group had normal cognitive function, no history of neurological disease; and (5) The included studies were Cohort or case–control studies (only initial data was extracted from the cohort study data).

The exclusion criteria were demonstrated as follows: (1) letters, editorials, summaries, conference abstracts, case reports, or publications without sufficient information; (2) unable to extract valid outcome data from the literature; and (3) articles were excluded if they measured marker concentrations in postmortem samples, and had sample size less than 5, or used the samples that overlapped with other studies.

### Data extraction and quality evaluation

Two participants (Fu and Huang) extracted data separately and a third (Bao) investigator verified the data abstracted by the two authors to avoid mistakes and bias. The following data were extracted from each included study: author's name, publication year, country, sample size, age and gender distribution, diagnostic criteria for AD, and platelet-associated markers data. We use Endnote X9 for literature selection and collation. In addition to using the duplicate function of the document management software, we carried out manual verification. In the case of missing data, we used get-data software to extract the relevant data of the article chart, or contacted the corresponding author asking for the relevant data. Quality assessments of all potentially eligible studies were conducted using the Newcastle- Ottawa Scale (NOS) [16]. The evaluation item consists of 3 parts (selection, comparability, exposure or outcome) with a maximum score of 9. Studies with NOS scores lower than five were recognized to be of inferior quality and therefore excluded. The systematic review assessed platelet markers measured in less than three studies qualitatively.

### Statistical analysis

Standardized mean difference (SMD) with 95% CI was used to compare the platelet-associated markers between AD patients and healthy controls. When the forest map effect box (95%CI of SMD) did not cross the 0 limits, the effect value was statistically significant (test for overall effect *P* < 0.05). Heterogeneity analysis was assessed by using the Cochrane Q test and I^2^ statistic. I^2^ > 50% or *P* < 0.1 represented substantial heterogeneity and random-effects model was chosen. To assess each study's influence on the pooled estimate, sensitivity analysis was applied by removing each study by turns, and meta-regression was used to explore the causes of heterogeneity. Egger's tests estimated publication bias. Probability value *P* value < 0.05 was considered to be statistically significant. All statistical analysis of this meta-analysis was performed using Stata version 15.0 software (supplementary material 2).

## Results

### Literature search findings

**Fig. 1:**
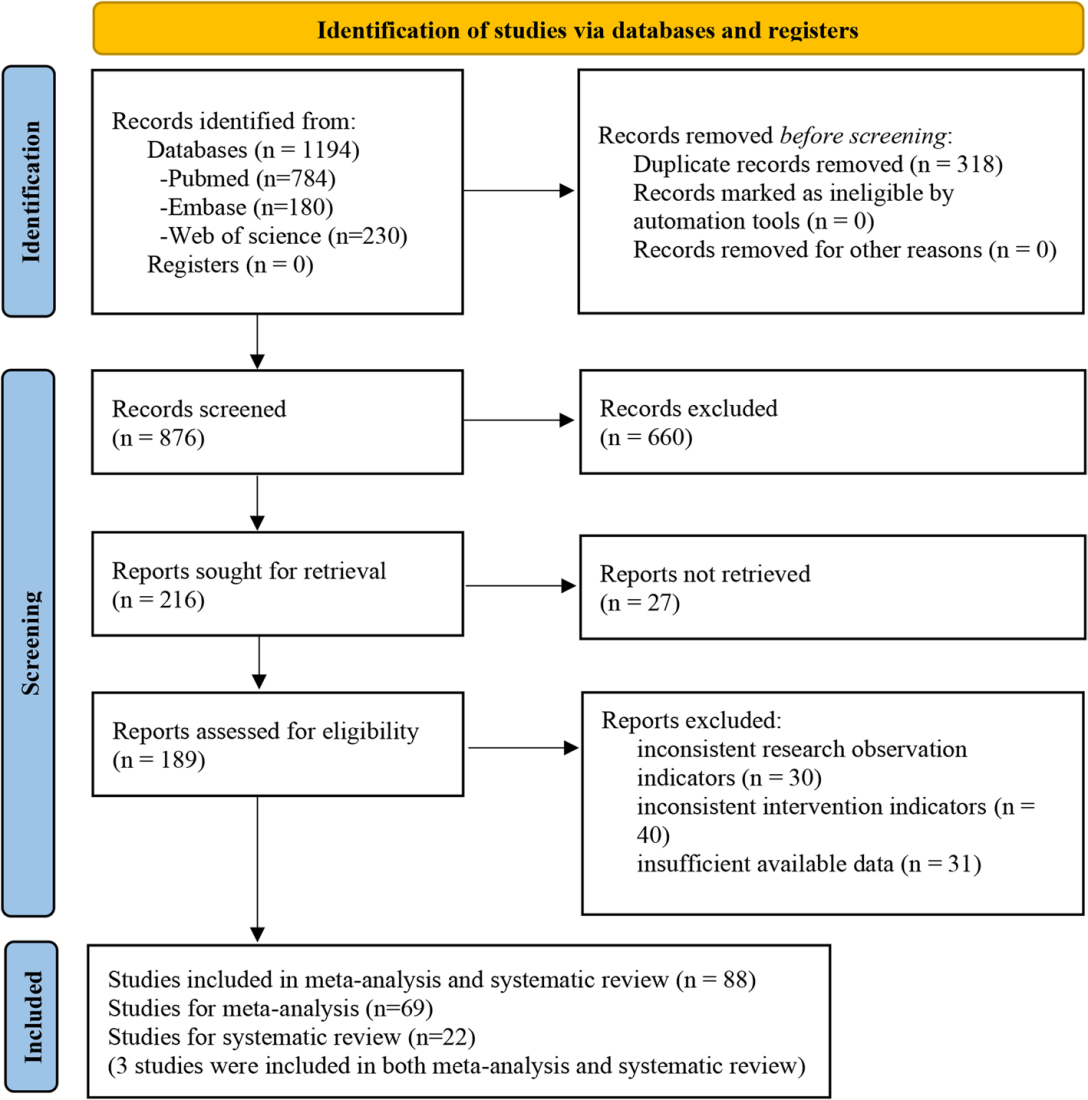
Literature screening flow chart

The initial literature search generated altogether 1194 records (Fig. 1). A total of 88 studies, including 3 Cohort studies and 85 Case–control studies, were ultimately included, and these studies included 1189 subjects with AD and 1214 controls. Among these studies (69 studies), 20 markers, including 662 subjects with AD and 680 controls, were quantitatively analyzed in the meta-analysis and baseline characteristics were shown on supplementary material 3, while among them (22 studies), 24 markers were reviewed systematically because there were less than three studies for each marker (supplementary material 4). Quality assessments of the studies were detailed in supplementary material 5. Individual marker performances and heterogeneity analyses were shown in supplementary material 6.

### Comparisons between AD and platelet-associated marker levels

**Fig. 2:**
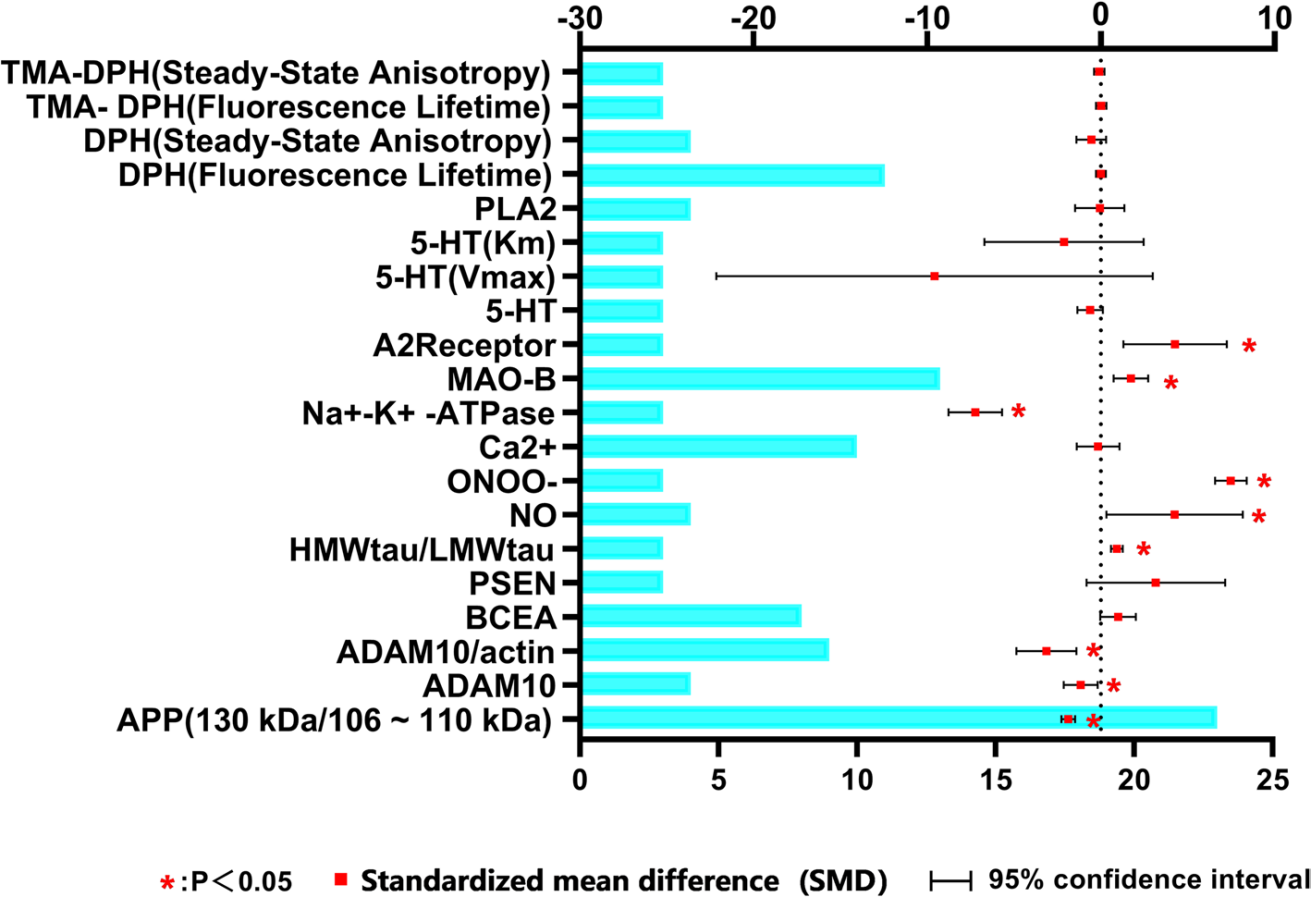
SMD (95%Confidence Interval) of platelet-associated markers for meta-analysis. The bar chart represents the number of articles for meta-analysis

The SMD results showed that patients with AD had lower levels of APP ratio (SMD: -1.89; 95% CI -2.28 to -1.49, *p* < 0.05), ADAM10/actin (SMD: -3.13; 95% CI -4.86 to -1.40, *p* < 0.05), ADAM10 (SMD: -1.16; 95% CI -2.13 to -0.18, *p* < 0.05), and Na^+^-K^+^ -ATPase (SMD: -7.23; 95% CI -8.77 to -5.68, *p* < 0.05), but higher levels of high molecular weight (HMW) / low molecular weight (LMW) tau (SMD: 0.92; 95% CI 0.58 to 1.25, *p* < 0.05), adenosine A_2_ receptor (SMD: 4.27; 95% CI 1.29 to 7.25, *p* < 0.05), MAO-B (SMD: 1.73; 95% CI 0.73 to 2.72, *p* < 0.05), NO production (SMD: 4.25; 95% CI 0.32 to 8.17, *p* < 0.05) and ONOO^−^ production (SMD: 7.33; 95% CI 6.57 to 8.39, *p* < 0.05) (Fig. 2). There were no significant differences in the levels of BACE-1, PSEN-1, 5-HT, Ca^2+^, Phospholipase A_2_ (PLA_2_), DPH and I-[4-(trimethylamino) phenyl]-6-phenyl-1,3,5, hexatriene (TMA-DPH), between AD patients and control subjects (Fig. 2).

### Investigation of heterogeneity

High heterogeneity was found in most comparisons (supplementary material 6). Sensitivity analyses indicated that the removal of any study had no significant effect on the results of the meta-analysis result (supplementary material 7). Significant publication bias was not found in most studies, as demonstrated by the funnel plots and confirmed by the Egger’s tests (supplementary materials 7), but publication bias was detected in studies focusing on APP ratio (Egger's intercept -3.67, *p* = 0.001), ADAM10/actin (Egger's intercept -3.53, *p* = 0.010), and MAO-B (Egger's intercept 2.58, *p* = 0.025) (supplementary material 7). The heterogeneity of the markers with publication bias was detected by the shear and complement method (supplementary material 7).

### Systematic review

**Fig. 3:**
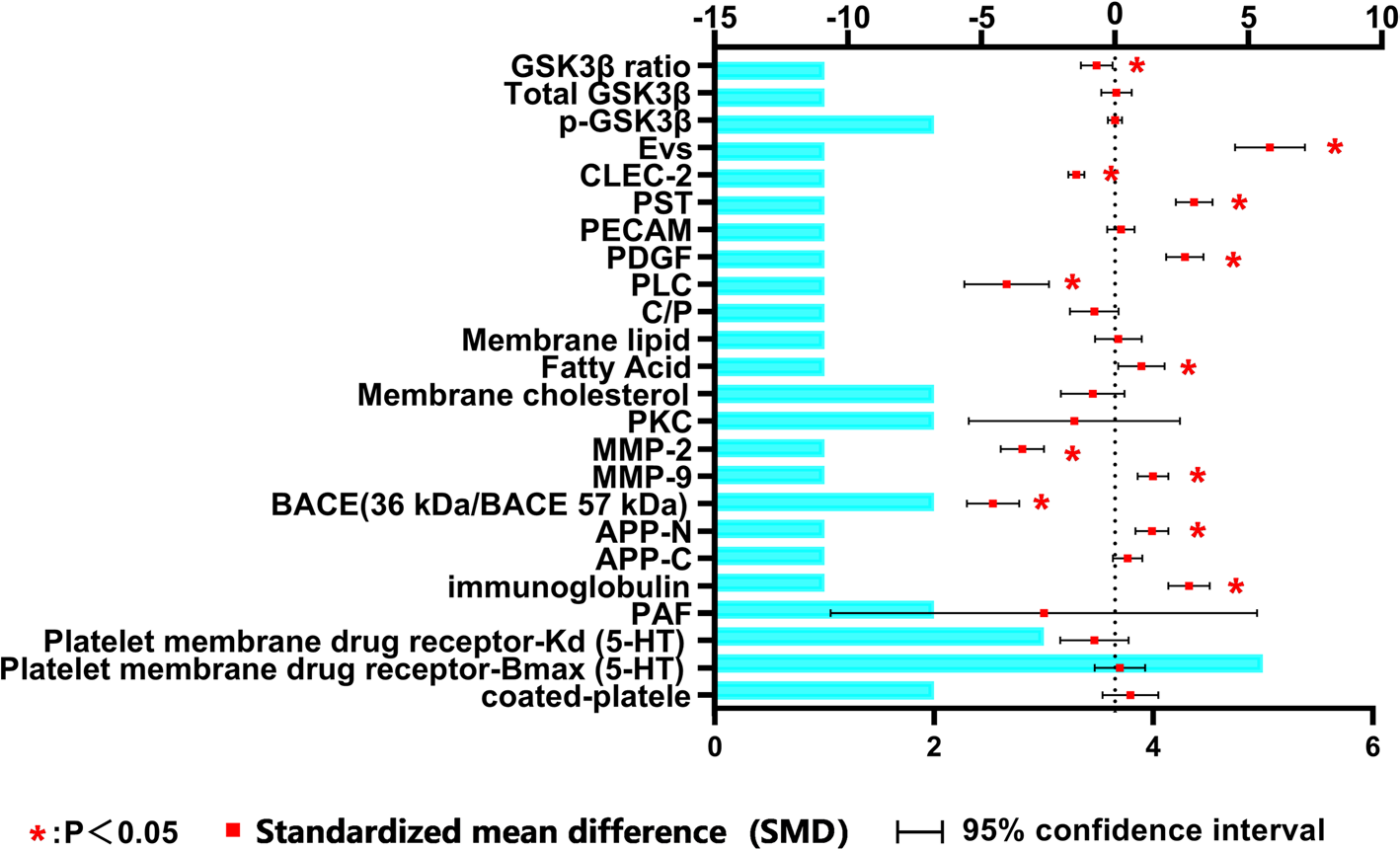
SMD (95%Confidence Interval) of platelet-associated markers for systematic review. The bar chart represents the number of articles for systematic review. Platelet membrane drug receptor: different drugs act on platelet membrane 5-HT receptor (Although there have been more than three studies on this marker, the results are classified as a systematic review because different drugs act on this receptor)

Due to the limited number of related studies on some peripheral platelet-related markers, systematic reviews were conducted (supplementary material 2). The results of systemic review showed that the levels of platelet immunoglobulin, APP-N, BACE (36 kDa/BACE 57 kDa), matrix metalloproteinases-9 (MMP-9), MMP-2, platelet membrane fatty acids, phospholipase C (PLC), phenolsulphotransferase (PST), plasma-derived growth factor (PDGF), C-type lectin-like receptor 2(CLEC2), extracellular vesicles (EVs), glycogen synthase kinase 3-beta (GSK3β) ratios were significantly different between AD patients and controls (Fig. 3), but further studies were needed to verify the alterations of these markers in AD patients.

## Discussion

### The APP Processing System

A meta-analysis in 2017 found that the APP ratio in peripheral platelets of AD patients was significantly lower than that in controls [17]. Our meta-analysis updated relevant data in recent years, and we also found decreased APP ratio in AD patients’ platelets [17]. APP is the precursor of Aβ protein and is highly expressed in the brain and platelets. Aβ protein secreted by platelets has been reported to contain a small number of intact 140–150-KD APPs and a large number of carboxy-truncated 120–130-KD and 110-KD APPs. These carboxy-truncated APPs may undergo further cleavage before the activated platelets are released into granules [18]. Platelet-released APP forms may have growth factor-related functions. 90-KD APP has been shown to promote the growth of cultured fibroblasts and neurons. However, it is more likely that APP containing the serine protease inhibitor form may be released by platelets and the platelet APP also participate in the clotting cascade by inhibiting activated clotting pathway enzymes. Studies have found that platelet APP ratio decreased gradually with the progression of the disease, suggesting that this peripheral parameter changed in the early stage of AD, and platelet APP ratio also significantly altered with the clinical status progressed [18]. In addition, some studies conducted diagnostic studies on peripheral platelet APP ratio found that its accuracy could reach 90.5% and its sensitivity could reach 80% [19]. Therefore, the platelet APP isoform ratios could be used as a periphery biomarker in diagnosing and developing AD.

Currently, results on platelet APP secretase in AD patients are controversial, and there is no relevant meta-analysis. Our meta-analysis found that ADAM10 level in peripheral platelets was significantly lower in AD patients than in healthy controls, consistent with the findings in the CNS [20]. ADAM10 is a type I membrane glycoprotein of 748 amino acids, responsible for the exfoliation of extracellular domains and regulating the hydrolysis of multiple proteins in the membrane. ADAM10 synaptic localization and activity decreased in AD patients, leading to an increase in Aβ level and damaging the plasticity of synaptic structures. Some studies have found that the decrease of ADAM10 platelet expression level is related to the disease stage of AD. Other study also found that the level of ADAM10 in platelets in AD patients was significantly lower than that in healthy controls, and decreased significantly with the increase of disease severity in AD patients [21]. Therefore, platelet ADAM10 is an applicable peripheral biomarker that can reflect the pathological changes and disease severity of AD.

Our analysis found that platelet BACE1 was higher in AD patients than in healthy controls, but the increase was insignificant (*P* = 0.059). Besides, in the systematic review we found that the platelet BACE isoform ratio (36KDa/57KDa) appeared to differ between AD patients and controls. BACE1 is an enzyme having β-secretase activity, and β-secretase activity was found to be significantly increased in platelets of AD patients and correlated with the MMSE score [22]. A study of platelet APP in AD showed a significant increase in cell-associated APP fragments after β-secretase cleavage [23]. Catalysis by APP pyrolysis to produce Aβ N- terminus was originally referred to as β-secretase activity and is a rate-limiting step in Aβ production. Several postmortem brain studies have found increased levels of β -secretase activity in BACE protein and up-regulation of BACE mRNA in brain regions associated with AD [24]. Therefore, more studies are needed to verify the alteration of BACE-1 and BACE isoform ratio in peripheral platelet of AD patients, and the association with the central pathological changes.

Our meta-analysis found no significant difference in platelet PSEN-1 level between AD patients and healthy controls; this finding was different from previous study [25]. The PSEN protein encoded by PSEN-1 and PSEN-2 genes is an important component of γ-secretase [26]. Mutations in PSEN-1 gene mutation result in conformational changes, which further affect γ -secretase activity and increase the production of Aβ42 [26]. However, there is no consistent finding in the PSEN-1 level in the CNS of AD patients. This inconsistency may be caused by the fact that PSEN-1 is involved in both amyloidosis and non-amyloidosis pathways of APP, and less is known about other pathophysiological functions of this protein. Therefore, the role of platelet PSEN-1 in APP metabolism in AD patients still needs to be further explored. Besides, some studies analyzed the value of combined utilization of the platelet APP ratio and APP secretase (ADAM10、BACE1 and PSEN-1) in AD, and found that the combination has a sensitivity of 88.9%, providing its potential application value in AD diagnosis and screening [27].

In addition to the significant findings in Aβ protein pathway, we also found that, in AD patient the ratio of HMW to LMW Tau in the peripheral platelet was significantly higher than that in healthy controls, with low heterogeneity and bias. Hyperphosphorylation of Tau protein leads to microtubule separation and entanglement aggregation, which is one of the neuropathological hallmarks of AD. Tau protein is also present in peripheral platelet. HMW human tau variants have been described mainly in the peripheral nervous system and neural crista-derived cell lines, and it may be related to the aggregation of tau near 60KD. The aggregation of such polymers may be related to the pathophysiology of the AD in the central nervous system and peripheral blood cells [28]. Besides, the high HMW/ LMW Tau ratio was correlated with the severity of cognitive impairment [29]. The Tau protein ratio in platelets could reflect the central pathology of AD, and has a certain value in AD diagnosis (sensitivity 71.43%, specificity 69.23%) [30].

### Oxidative stress system

Except for the APP Processing System, oxidative stress is also important pathogenesis of AD. Our meta-analysis found that the levels of NO and ONOO- production in platelets of AD patients were higher than those in the healthy control group, suggesting the involvement of oxidative stress in platelets of AD patients. During the chronic progression of AD, NO overexpression may lead to neurodegenerative disease through protein nitration induced by reactive peroxynitrite [9]. The complex pathology of AD is not limited to the brain. Oxidative stress was detected in platelets during normal aging, and it was even more intense in AD [31]. We found that platelet levels of NO and ONOO^−^ production were significantly elevated in AD patients, which may be good biomarkers for peripheral oxidative stress in AD patients. It was noticed that three of the four studies included in analyzing NO and ONOO^−^ production came from the same laboratory, while the inconsistent findings reported by the fourth study was from a different laboratory. Therefore, race and laboratory bias should be considered when explaining the NO and ONOO^−^ production in AD. In addition, Na + /K + -ATPase activity in the platelet membrane was found to be significantly reduced in platelets in AD patients, compared to control individuals. Na + /K + -ATPase is an important part of ion homeostasis. Because ATPase is rich in sulfhydryl (SH), its sulfhydryl group may be the target of free radical OS induction [32]. Studies found that ONOO^−^ can inhibit Na + /K + -ATPase activity of cell membrane [32]. The reduction of Na + /K + -ATPase activity in platelets of AD patients indicates the connection between ion channel and oxidative stress in platelets, and their contributions to the pathophysiological process related to AD.

However, our meta-analysis found that Ca^2+^ accumulation was not significant different in platelets of AD patients. Oxidative stress leads to induced cellular dysfunction, resulting in calcium ion accumulation and neuronal death [33]. Although some studies have found increased Ca^2+^ concentration in platelet APP, the alteration of Ca^2+^ influx through membrane calcium channel and the change of Ca^2+^ release from internal stores is complex; more studies are required to verify the role of platelet calcium concentration in pathological condition of AD.

### Other metabolic systems

This study also analyzed other neurotransmitter systems and found that platelet MAO-B levels were significantly higher in AD than in controls. Activated MAO induces deposition of Aβ through abnormal cleavage of APP. In addition, activated MAO contributes to the formation of neurofibrillary tangles, the loss of neural cells, and cognitive impairment [34]. Increased MAO-B activity in aging brains leads to increased oxidative stress [35]. Platelets serve as a good peripheral model for AD, and we found significant differences in platelet MAO-B levels between AD patients and controls, consistent with changes in the CNS. Although some neurotransmitters (e.g. cholinesterase, 5-HT) were found to be affected by MAO activation after cognitive impairment [35], our results found no significant difference in platelet 5-HT between AD patients and controls. This may be due to the very slow oxidation of 5-HT by MAO-B in the periphery platelets; MAO-B was found to be poor in oxidizing serotonin in platelets [36]. These results suggest that the MAO-B metabolic pathway plays a certain role in both central and peripheral system of AD.

The meta-analysis found that peripheral platelet A_2A_ adenosine receptor expression was significantly higher in AD patients than in controls, consistent with brain tissue changes. Adenosine is an important endogenous neuromodulator that affects a variety of neurodegenerative diseases by interacting with four G-protein-coupled receptors A_1_, A_2A_, A_2B_ and A_3_ [37]. A_2A_ adenosine receptor antagonists have neuroprotective effects in neurodegenerative diseases [37]. Injury of A_2A_ adenosine receptor prevents Aβ -induced synaptic toxicity and cognitive deficits in different animal models [38]. Therefore, the increased platelet A_2A_ adenosine receptor expression, may play a role in the pathological processes of AD, and could be used as a potential therapeutic target. In addition, our systematic analysis also found that some biomarkers that may be meaningful in platelets in AD, including GSK3β, CLEC2, EVs, PST, PDGF, PLC, membrane lipoprotein, MMPs, APP-N, APP-C, immunoglobulin. GSK-3 is a proline-oriented serine/threonine kinase that was first identified due to its role in the regulation of glycogen metabolism. GSK3 has been found to play an important role in AD through a variety of pathways. GSK3β can modify APP and tau proteins [39]. Forlenza’s study found reduced rates of P-GSK3β in peripheral plaques of AD patients, consistent with the findings in CNS. However, there are only a few relevant studies on these related markers in platelets, more studies are needed to verify the role of these markers in AD.

### Limitations

Although this study is the first comprehensive and systematic review of previous studies on platelet-associated markers in patients with AD, noticeable heterogeneity exists, and the present meta-analysis still has some limitations. First, since the severity of disease in the AD group included in the meta-analysis was different, and most studies did not provide disease severity data, the impact of disease severity on platelet-associated markers in AD patients is unknown. Second, some of studies were published before the year 2000, and many were not shown in the form of the mean (standard deviation). Although we tried to contact the authors, relevant results were still not obtained. In addition, grey literature was not searched in this study. Thirdly, different experimental methods and conditions of different studies may cause some heterogeneity. Fourthly, some markers were positive in individual studies but negative in aggregate analyses. The first reason is that the number of studies is not enough, and the objectives, perspectives and contents of the studies are not enough. Due to incomplete information in the included studies, the potential mechanism of these platelet-associated markers and their potential correlation with other clinical regulatory factors (such as race, medication status, course of disease, *APOE* gene carrying and mental health level) cannot be further studied.

## Conclusions

In this meta-analysis, compared with the control group, APP ratio, ADAM10, ADAM10/actin, Na + -K + -ATPase in platelets of AD group significantly decreased; HMW tau/LMW tau, adenosine A_2_ receptor, MAO-B, NO and ONOO^−^ productions significantly increased; BACE-1, PSEN-1, 5-HT, Ca^2+^, DPH, TMA-DPH had no significant difference. The systematic review found that platelet immunoglobulin, APP-N, BACE (36 KD/57KD), MMP-9, MMP-2, platelet membrane fatty acids, PLC, PST, PDGF, CLEC2, EVs, GSK3β ratios may be biomarkers worthy of further attention in the future. The present study indicates that platelet is a good peripheral model to study those metabolic mechanisms in CNS of AD patients; many platelet markers may be useful in the diagnosis of AD, and could be used as potential therapeutic targets.

## Supplementary Information

**Additional file 1:** **Table S1.** Search strategy of different databases.

**Additional file 2:** **Table S2.** statistical analysis code of meta-analysis in Stata software

**Additional file 3:** **Table S3.** Study calculating the 5-HT of Platelet. **Table S4.** Study calculating the tau of Platelet. **Table** **S5.** Study calculating the APP of Platelet. **Table S6.** Study calculating the BACE-1 of Platelet. **Table S7.** Study calculating the PSEN-1 of Platelet. **Table S8.** Study calculating the ADAM-10 of Platelet. **Table S9.** Study calculating the Ca^2+^ of Platelet. **Table** **S10.** Study calculating the PLA2 of Platelet. **Table S11.** Study calculating the NO of Platelet. **Table S12. **Study calculating the Platelet membrane fluidity. **Table S13.** Study calculating the adenosine A_2._receptor of Platelet. **Table S14.** Study calculating the Na^+^-K^+^ -ATPase of Platelet. **Table S15.** Study calculating the MAO-B of Platelet.

**Additional file 4: Table S16.** Study calculating the coated-platelet. **Table S17.** Study calculating the Platelet membrane drug receptor (5-HT). **Table S18.** Study calculating the PAF of Platelet. **Table S19.** Study calculating the immunoglobulin of Platelet. **Table S20.** Study calculating the BACE (36 kDa/BACE 57 kDa) of Platelet. **Table S21.** Study calculating the APP of Platelet. **Table S22.** Study calculating the MMP-9 of Platelet. **Table S23.** Study calculating the MMP-2 of Platelet. **Table S24.** Study calculating the PKC of Platelet. **Table S25.** Study calculating the Lipid composition of platelet membrane. **Table S26.** Study calculating the PDGF of Platelet. **Table S27.** Study calculating the PECAM-1 of Platelet. **Table S28.** Study calculating the PST of Platelet. **Table S29.** Study calculating the CLEC-2 of Platelet. **Table S30.** Study calculating the EVs of Platelet. **Table S31.** Study calculating the GSKβ of Platelet.

**Additional file 5: Table 32.** quality assessment of NOS.

**Additional file 6:** **Figure S1.** Forest plot for APP(130kDa106-110kDa. **Figure S2.** Forest plot for ADAM10. **Figure S3.** Forest plot for ADAM10/actin. **Figure S4.** Forest plot for BCEA. **Figure S5.** Forest plot for PSEN. **Figure S6.** HMWtau/LMWtau. **Figure S7.** Forest plot for NO production. **Figure S8.** Forest plot for ONOO- production. **Figure S9.** Forest plot for Ca2+. **Figure S10.** Forest plot for Na+-K+-ATPase. **Figure S11.** Forest plot for MAO-B. **Figure S12.** Forest plot for 5-HT. **Figure S13.** Forest plot for 5-HT(Vmax). **Figure S14.** Forest plot for 5-HT(Km). **Figure S15.** Forest plot for A2 Receptor. **Figure S16.** Forest plot for PLA2. **Figure S17.** Forest plot for DPH(Fluorescence Lifetime). **Figure S18.** Forest plot for DPH(Steady-State Anisotropy). **Figure S19.** Forest plot for TMA-DPH(Fluorescence Lifetime). **Figure S20.** Forest plot for TMA-DPH(Steady-State Anisotropy).

**Additional file 7: Figure S21.** Sensitivity analysis for APP(130 kDa/106-110kDa). **Figure S22.** funnel plot of APP(130 kDa/106-110kDa); Egger’s test: *p*=0.001. **Figure S23.** filled funnel plot of APP(130 kDa/106-110kDa). **Figure S24.** Sensitivity analysis for ADAM-10. **Figure S25.** funnel plot of ADAM-10; Egger’s test: *p*＞0.05. **Figure S26.** Sensitivity analysis for ADAM-10/actin. **Figure S27.** funnel plot of ADAM-10/actin; Egger’s test: *p*=0.010. **Figure S28.** filled funnel plot of ADAM-10/actin. **Figure S29.** Sensitivity analysis for BACE-1. **Figure S30.** funnel plot of BCEA-1; Egger’s test: *p*＞0.05**.** **Figure S31.** Sensitivity analysis for PSEN-1. **Figure S32.** funnel plot of PSEN-1; Egger’s test: *p*＞0.05. **Figure S33.** Sensitivity analysis for HMWtau/LMWtau. **Figure S34.** funnel plot of HMWtau/LMWtau; Egger’s test: *p*＞0.05. **Figure S35.** Sensitivity analysis for NO production. **Figure S36.** funnel plot of NO production; Egger’s test: *p*＞0.05. **Figure S37.** Sensitivity analysis for ONOO- production. **Figure S38.** funnel plot of ONOO- production; Egger’s test: *p*＞0.05. **Figure S39.** Sensitivity analysis for Ca2+. **Figure S40.** funnel plot of Ca2+; Egger’s test: *p*＞0.05. **Figure S41.** Sensitivity analysis for Na+-K+ -ATPase. **Figure S42.** funnel plot of Na+ -K+ -ATPase; Egger’s test: *p*＞0.05. **Figure S43.** Sensitivity analysis for MAO-B. **Figure S44.** funnel plot of MAO-B; Egger’s test: *p*=0.025. **Figure S45.** filled funnel plot of MAO-B. **Figure S46.** Sensitivity analysis for 5-HT. **Figure S47.** funnel plot of 5-HT; Egger’s test: *P*＞0.05. **Figure S48.** Sensitivity analysis for 5-HT(Bmax). **Figure S49.** funnel plot of 5-HT(Bmax); Egger’s test: *P*＞0.05. **Figure S50.** Sensitivity analysis for 5-HT(Vmax). **Figure S51.** funnel plot of 5-HT(Vmax); Egger’s test: *P*＞0.05. **Figure S52.** Sensitivity analysis for 5-HT(Km). **Figure S53.** funnel plot of 5-HT(Km); Egger’s test: *P*＞0.05. **Figure S54.** Sensitivity analysis for A2Receptor. **Figure S55.** funnel plot of A2Receptor; Egger’s test: *P*＞0.05. **Figure S56.** Sensitivity analysis for PLA2. **Figure S57.** funnel plot of PLA2 ;Egger’s test: *P*＞0.05. **Figure S58.** Sensitivity analysis for DPH(Steady-State Anisotropy). **Figure S59.** funnel plot of DPH(Steady-State Anisotropy); Egger’s test: *P*＞0.05. **Figure S60.** Sensitivity analysis for DPH(Fluorescence Lifetime). **Figure S61.** funnel plot of DPH(Fluorescence Lifetime); Egger’s test: *P*＞0.05. **Figure S62.** Sensitivity analysis for TMA-DPH(Steady-State Anisotropy). **Figure S63.** funnel plot of TMA-DPH(Steady-State Anisotropy); Egger’s test: *P*＞0.05. **Figure S64.** Sensitivity analysis for TMA- DPH(Fluorescence Lifetime). **Figure S65.** funnel plot of TMA- DPH(Fluorescence Lifetime); Egger’s test: *P*＞0.05.

## Data Availability

Detailed data have been shown in the supplementary materials. All data generated or analysed during this study are included in this published article and its supplementary information files.

## Abbreviations

DHP: 1,6-dipheny1-1,3,5-hexatriene
HMW: High molecular weight
LMW: Low molecular weight
Bmax: The maximum number of binding sites
Kd: Apparent binding affinity

## References

[1] Kirson NY, Desai U, Ristovska L (2016). Assessing the economic burden of Alzheimer's disease patients first diagnosed by specialists. BMC Geriatr.

[2] Patterson C (2018). Alzheimer's Disease International.

[3] Reinhard C, Hébert SS, De Strooper B (2005). The amyloid-beta precursor protein: integrating structure with biological function. EMBO J.

[4] Tang K, Hynan LS, Baskin F, Rosenberg RN (2006). Platelet amyloid precursor protein processing: a bio-marker for Alzheimer's disease. J Neurol Sci.

[5] Diniz BS, Pinto Júnior JA, Forlenza OV (2008). Do CSF total tau, phosphorylated tau, and beta-amyloid 42 help to predict progression of mild cognitive impairment to Alzheimer's disease? A systematic review and meta-analysis of the literature. World J Biol Psychiatry.

[6] Zainaghi IA, Forlenza OV, Gattaz WF (2007). Abnormal APP processing in platelets of patients with Alzheimer's disease: correlations with membrane fluidity and cognitive decline. Psychopharmacol.

[7] Bermejo-Bescós P, Martín-Aragón S, Jiménez-Aliaga K (2013). Processing of the platelet amyloid precursor protein in the mild cognitive impairment (MCI). Neurochem Res.

[8] Zubenko GS, Huff FJ, Beyer J, Auerbach J, Teply I (1988). Familial risk of dementia associated with a biologic subtype of Alzheimer's disease. Arch Gen Psychiatry.

[9] Vignini A, Nanetti L, Moroni C (2007). Modifications of platelet from Alzheimer disease patients: a possible relation between membrane properties and NO metabolites. Neurobiol Aging.

[10] Butterfield DA (1997). beta-Amyloid-associated free radical oxidative stress and neurotoxicity: implications for Alzheimer's disease. Chem Res Toxicol.

[11] Kawamoto EM, Munhoz CD, Glezer I (2005). Oxidative state in platelets and erythrocytes in aging and Alzheimer's disease. Neurobiol Aging.

[12] Yu Jie, Jia Jian-ping (2009). Platelet function in patients with Alzheimer disease: analysis of 40 cases. Zhonghua Yi Xue Za Zhi.

[13] Garcia-Alloza M, Gil-Bea FJ, Diez-Ariza M (2005). Cholinergic-serotonergic imbalance contributes to cognitive and behavioral symptoms in Alzheimer's disease. Neuropsychologia.

[14] Racchi M, Govoni S (2003). The pharmacology of amyloid precursor protein processing. Exp Gerontol.

[15] Camacho A, Dimsdale JE (2000). Platelets and psychiatry: lessons learned from old and new studies. Psychosom Med.

[16] Wells GA, Shea B, O'Connell D, Peterson J, Welch V, Losos M, et al. The Newcastle-Ottawa Scale (NOS) for assessing the quality if nonrandomized studies in meta-analyses. Available from: URL: http://www.ohri.ca/programs/clinical_epidemiology/oxford.htm [cited 2009 Oct 19]

[17] Shi Y, Gu L, Alsharif AA, Zhang Z (2017). The Distinction of Amyloid-β Protein Precursor (AβPP) Ratio in Platelet Between Alzheimer's Disease Patients and Controls: A Systematic Review and Meta-Analysis. J Alzheimers Dis.

[18] Evin G, Zhu A, Holsinger RM, Masters CL, Li QX (2003). Proteolytic processing of the Alzheimer's disease amyloid precursor protein in brain and platelets. J Neurosci Res.

[19] Baskin F, Rosenberg RN, Iyer L, Hynan L, Cullum CM (2000). Platelet APP isoform ratios correlate with declining cognition in AD. Neurology.

[20] Tyler SJ, Dawbarn D, Wilcock GK, Allen SJ (2002). alpha- and beta-secretase: profound changes in Alzheimer's disease. Biochem Biophys Res Commun.

[21] Manzine PR, de França Bram JM, Barham EJ (2013). ADAM10 as a biomarker for Alzheimer's disease: a study with Brazilian elderly. Dement Geriatr Cogn Disord.

[22] Johnston JA, Liu WW, Todd SA (2005). Expression and activity of beta-site amyloid precursor protein cleaving enzyme in Alzheimer's disease. Biochem Soc Trans.

[23] Zimmermann M, Borroni B, Cattabeni F, Padovani A, Di Luca M (2005). Cholinesterase inhibitors influence APP metabolism in Alzheimer disease patients. Neurobiol Dis.

[24] Fukumoto H, Cheung BS, Hyman BT, Irizarry MC (2002). Beta-secretase protein and activity are increased in the neocortex in Alzheimer disease. Arch Neurol.

[25] Borroni B, Agosti C, Marcello E, Di Luca M, Padovani A (2010). Blood cell markers in Alzheimer Disease: Amyloid Precursor Protein form ratio in platelets. Exp Gerontol.

[26] Liu X, Liu Y, Ji S (2021). Secretases Related to Amyloid Precursor Protein Processing. Membranes (Basel)..

[27] Bram JMF, Talib LL, Joaquim HPG, Sarno TA, Gattaz WF, Forlenza OV (2019). Protein levels of ADAM10, BACE1, and PSEN-1 in platelets and leukocytes of Alzheimer's disease patients. Eur Arch Psychiatry Clin Neurosci.

[28] Maccioni RB, Farías G, Morales I, Navarrete L (2010). The revitalized tau hypothesis on Alzheimer's disease. Arch Med Res.

[29] Farías G, Pérez P, Slachevsky A, Maccioni RB (2012). Platelet tau pattern correlates with cognitive status in Alzheimer's disease. J Alzheimers Dis.

[30] Guzmán-Martínez L, Tapia JP, Farías GA, González A, Estrella M, Maccioni RB (2019). The Alz-tau Biomarker for Alzheimer's Disease: Study in a Caucasian Population. J Alzheimers Dis.

[31] Teunissen CE, Lütjohann D, von Bergmann K (2003). Combination of serum markers related to several mechanisms in Alzheimer's disease. Neurobiol Aging.

[32] Muriel P, Castañeda G, Ortega M, Noël F (2003). Insights into the mechanism of erythrocyte Na+/K+-ATPase inhibition by nitric oxide and peroxynitrite anion. J Appl Toxicol.

[33] Varadarajan S, Yatin S, Aksenova M, Butterfield DA (2000). Review: Alzheimer's amyloid beta-peptide-associated free radical oxidative stress and neurotoxicity. J Struct Biol.

[34] Behl T, Kaur D, Sehgal A (2021). Role of Monoamine Oxidase Activity in Alzheimer's Disease: An Insight into the Therapeutic Potential of Inhibitors. Molecules..

[35] Ramsay RR (2016). Molecular aspects of monoamine oxidase B. Prog Neuropsychopharmacol Biol Psychiatry.

[36] Oreland L (2004). Platelet monoamine oxidase, personality and alcoholism: the rise, fall and resurrection. Neurotoxicology.

[37] Borea PA, Gessi S, Merighi S, Vincenzi F, Varani K (2018). Pharmacology of Adenosine Receptors: The State of the Art. Physiol Rev.

[38] Canas PM, Porciúncula LO, Cunha GM (2009). Adenosine A2A receptor blockade prevents synaptotoxicity and memory dysfunction caused by beta-amyloid peptides via p38 mitogen-activated protein kinase pathway. J Neurosci.

[39] Lucas JJ, Hernández F, Gómez-Ramos P, Morán MA, Hen R, Avila J (2001). Decreased nuclear beta-catenin, tau hyperphosphorylation and neurodegeneration in GSK-3beta conditional transgenic mice. EMBO J.

