# Supplementary material for: Meta-analysis and systematic review of peripheral platelet-associated biomarkers to explore the pathophysiology of alzheimer's disease
Source: BMC Neurol. 2023 Feb 11;23:66. doi: 10.1186/s12883-023-03099-5 (PMC9921402; doi:10.1186/s12883-023-03099-5)
Supplement: Supplementary file 1 — Additional file 1: Table S1. Search strategy of different databases. [file 12883_2023_3099_MOESM1_ESM.docx]

Table S1: Search strategy of different databases.

| Database | Search strategy |
| --- | --- |
| Pubmed | ((((((((Blood Platele[Title/Abstract]) OR (Blood Platelet[Title/Abstract])) OR (Platelet, Blood[Title/Abstract])) OR (Platele, Blood[Title/Abstract])) OR (Thrombocytes[Title/Abstract])) OR (Thrombocyte[Title/Abstract])) OR (Platele [Title/Abstract])) OR (Platelet[Title/Abstract])) AND (((((((((((((((((((((((((Alzheimer's disease[Title/Abstract]) OR (Alzheimer Disease[Title/Abstract])) OR (Alzheimer Dementia[Title/Abstract])) OR (Alzheimer Dementias[Title/Abstract])) OR (Dementia Alzheimer[Title/Abstract])) OR (Alzheimer's Disease[Title/Abstract])) OR (Dementia Senile[Title/Abstract])) OR (Senile Dementia[Title/Abstract])) OR (Dementia Alzheimer Type[Title/Abstract])) OR (Alzheimer Type Dementia[Title/Abstract])) OR (Alzheimer Type Senile Dementia[Title/Abstract])) OR (Dementia Primary Senile Degenerative[Title/Abstract])) OR (Sclerosis Alzheimer[Title/Abstract])) OR (Alzheimer Syndrome[Title/Abstract])) OR (Alzheimer's Diseases[Title/Abstract])) OR (Alzheimer Diseases[Title/Abstract])) OR (Alzheimers Diseases[Title/Abstract])) OR (Senile Dementia Alzheimer Type[Title/Abstract])) OR (Dementia Presenile[Title/Abstract])) OR (Presenile Dementia[Title/Abstract])) OR (Alzheimer Disease Late Onset[Title/Abstract])) OR (Late Onset Alzheimer Disease[Title/Abstract])) OR (Alzheimer Disease Early Onset[Title/Abstract])) OR (Early Onset Alzheimer Disease[Title/Abstract])) OR (Presenile Alzheimer Dementia[Title/Abstract])) |
| Web of science | ((TS=(Alzheimer Dementia) OR TS=(Alzheimer Dementias) OR TS=(Alzheimer Disease) OR TS=(Alzheimer's Disease) OR TS=(Dementia, Senile) OR TS=(Senile Dementia) OR TS=(Presenile Dementia) OR TS=(Early Onset Alzheimer Disease) OR TS=(Presenile Alzheimer Dementia) OR TS=(Alzheimer Syndrome) OR TS=(Alzheimer Sclerosis))) AND (TS=(Blood Platelets) OR TS=(Blood Platelet) OR TS=(Platelet, Blood) OR TS=(Platelets, Blood) OR TS=(Thrombocytes) OR TS=(Thrombocyte) OR TS=(Platelets) OR TS=(Platelet)) |
| Embase | ('alzheimer disease':ab,ti OR 'alzheimer dementia':ab,ti OR 'senile dementia':ab,ti OR 'alzheimer sclerosis':ab,ti OR 'alzheimer syndrome':ab,ti OR 'presenile dementia':ab,ti OR 'early onset alzheimer disease':ab,ti OR 'presenile alzheimer dementia':ab,ti) and ('blood platelets':ab,ti OR 'blood platelet':ab,ti OR 'platelet, blood':ab,ti OR thrombocytes:ab,ti OR thrombocyte:ab,ti OR platelets:ab,ti OR platelet:ab,ti) |
