# Supplementary material for: Meta-analysis and systematic review of peripheral platelet-associated biomarkers to explore the pathophysiology of alzheimer's disease
Source: BMC Neurol. 2023 Feb 11;23:66. doi: 10.1186/s12883-023-03099-5 (PMC9921402; doi:10.1186/s12883-023-03099-5)
Supplement: Supplementary file 2 — Additional file 2: Table S2. statistical analysis code of meta-analysis in Stata software [file 12883_2023_3099_MOESM2_ESM.docx]

Table S2: statistical analysis code of meta-analysis in Stata software

| analytical method | statistical analysis code |
| --- | --- |
| heterogeneity | metan adn admean adsd controln controlmean controlsd,fixed label(namevar= study )  metaan _ES _seES,label( study )dl  metan adn admean adsd controln controlmean controlsd,random label(namevar= study )  metareg _ES,wsse( _seES ) eform |
| Sensitivity analyses | metaninf adn admean adsd controln controlmean controlsd, label(namevar=study) |
| Bias test | metafunnel _ES _seES  metabias6 _ES _seES |
| Shear and complement method | meta _ES _seES, id( study ) eform  metatrim _ES _seES,eform funnel |
