# Supplementary material for: Meta-analysis and systematic review of peripheral platelet-associated biomarkers to explore the pathophysiology of alzheimer's disease
Source: BMC Neurol. 2023 Feb 11;23:66. doi: 10.1186/s12883-023-03099-5 (PMC9921402; doi:10.1186/s12883-023-03099-5)
Supplement: Supplementary file 3 — Additional file 3: Table S3. Study calculating the 5-HT of Platelet. Table S4. Study calculating the tau of Platelet. Table S5. Study calculating the APP of Platelet. Table S6. Study calculating the BACE-1 of Platelet. Table S7. Study calculating the PSEN-1 of Platelet. Table S8. Study calculating the ADAM-10 of Platelet. Table S9. Study calculating the Ca2+ of Platelet. Table S10. Study calculating the PLA2 of Platelet. Table S11. Study calculating the NO of Platelet. Table S12. Study calculating the Platelet membrane fluidity. Table S13. Study calculating the adenosine A2.receptor of Platelet. Table S14. Study calculating the Na+-K+ -ATPase of Platelet. Table S15. Study calculating the MAO-B of Platelet. [file 12883_2023_3099_MOESM3_ESM.docx]

Table S3: Study calculating the 5-HT of Platelet.

| study | country | Diagnostic  criteria | Cognitive scores | P (n) | C (n) | Sex (P) (M/F) | Sex (C) (M/F) | Age(P) | Age(C) | MMSE  (P) | MMSE  (C) | Vmax(P) (pmol/min/10^7^ platelets) | Vmax(C) (pmol/min/10^7^ platelets) | Km(P)(10^-7^M) | Km(C)(10^-7^M) | 5-HT  ng/108 platelets (P) | 5-HT  ng/108 platelets (C) |
| --- | --- | --- | --- | --- | --- | --- | --- | --- | --- | --- | --- | --- | --- | --- | --- | --- | --- |
| Koren/1993 | Israel | NINCDS-ADRDA | NR | 26 | 29 | 12/14 | 11/18 | 74.3±1.15 | 77.2+1.47 | NR | NR | 3.21±0.13 | 5.82±0.20 | 0.54±0.01 | 0.50±0.01 |  |  |
| Inestrosa/1993 | Chile | NINCDS-ADRDA | NR | 11 | 20 | NR | NR | 68.3+2.0 | 70.1+2.1 | NR | NR | 2.1±0.2 | 5.0±0.2 | 0.44±0.01 | 0.66±0.02 |  |  |
| Kumar/1995 | USA | NINCDS-ADRDA | MMSE | 20 | 18 | 0/20 | 0/18 | 76.0±8.27 | 72.6±7.24 | NR | NR | 14.5±7.02 | 9.3±5.7 | 1.02±0.69 | 0.57±0.38 |  |  |
| kumar/2008 | USA | NINCDS-ADRDA | MMSE | 22 | 20 | 2/20 | 0/20 | 75.9±7.71 | 72.6±6.88 | NR | NR |  |  |  |  | 65.7±28.41 | 112.9±35.11 |
| Muck-Seler/2009 | Croatia | NINCDS-ADRDA | MMSE | 74 | 49 | 0/74 | 0/49 | 76.9±9.9 | 73.7±8.8 | 11.7±8.1 | 28.56±1.94 |  |  |  |  | 0.81±0.34 | 0.84±0.43 |
| Hochstrasser/2012 | Austria | NINCDS-ADRDA | MMSE | 34 | 26 | 6/28 | 14/12 | 79±1.1 | 72±1.2 | 18.1±1.2 | 27.7±0.9 |  |  |  |  | 529±88 | 565±79 |

Table S4: Study calculating the tau of Platelet.

| study | country | diagnostic  criteria | Cognitive scores | P  (n) | C  (n) | Sex (M/F) (P) | Sex (M/F) (C) | Age(P) | Age(C) | MMSE  (P) | MMSE  (C) | HMWtau/(LMWtau)  (P) | HMWtau/(LMWtau)  (C) |
| --- | --- | --- | --- | --- | --- | --- | --- | --- | --- | --- | --- | --- | --- |
| Neumann/2011 | Chile | NINCDS-ADRDA | MMSE | 15 | 10 | 7/8 | 3/7 | 80.9 (65–105) | 68.2 (52–79) | 15 (2–28) | 28.4 (28–30) | 3.893±2.506 | 0.594±0.364 |
| Slachevsky/2016 | Chile | NINCDS-ADRDA | MMSE | 53 | 37 | 21/32 | 13/24 | 73.62±6.64 | 71.3±5.18 | 21.7±5.57 | 28±2.19 | 2.17±1.21 | 1.4±0.43 |
| Guzm´an/2019 | Chile | NR | MMSE | 36 | 15 | 7/29 | 6/9 | 74.92±10.89 | 65.87±6.63 | 21.69±3.90 | 29.47±0.74 | 1.445±0.439 | 1.098±0.349 |

Table S5: Study calculating the APP of Platelet.

| study | country | diagnostic  criteria | Cognitive scores | P  (n) | C  (n) | Sex (M/F) (P) | Sex (M/F) (C) | Age(P) | Age(C) | MMSE  (P) | MMSE  (C) | APP  (130 kDa/106 ~ 110 kDa) (P) | APP  (130 kDa/106 ~ 110 kDa) (C) |
| --- | --- | --- | --- | --- | --- | --- | --- | --- | --- | --- | --- | --- | --- |
| Di Luca/1996 | Italy | NINCDS-ADRDA | MMSE | 10 | 15 | 3/7 | 7/8 | 67.3±12.9 | 61±14 | 19.6±1.8 | 29.4±0.6 | 0.160±0.048 | 0.760±0.28 |
| Rosenberg/1997 | America | NINCDS-ADRDA | NR | 15 | 19 | 4/11 | 5/14 | NR | NR | NR | NR | 5.61±0.41 | 7.72±0.57 |
| Di Luca/1998 | Italy | NINCDS-ADRDA | MMSE | 32 | 25 | 13/19 | 18/7 | 71.8±10.3 | 67.6±13.5 | 12.7±7.3 | 28.2±3.6 | 0.31±0.15 | 0.84±0.24 |
| Baskin/2000-1 | Dallas | NINCDS-ADRDA | MMSE | 10 | 11 | NR | NR | NR | NR | 8.3±2.13 | 28.91±0.20 | 3.57±0.47 | 8.31±0.27 |
| Baskin/2000-2 | Dallas | NINCDS-ADRDA | MMSE | 10 | 11 | NR | NR | NR | NR | 16.4±1.56 | 29.50±0.26 | 5.83±0.51 | 8.09±0.61 |
| Borroni/2001 | Italy | NINCDS-ADRDA | MMSE | 10 | 10 | 6/4 | 5/5 | 70.7±7.4 | 69.5±9.7 | 17.4±6.1 | 28.3±1.6 | 0.47±0.12 | 0.93±0.37 |
| Padovani/2001 | Italy | NINCDS-ADRDA | MMSE | 85 | 95 | 35/50 | 52/43 | 68.0±10.1 | 59.3±17.4 | 14.2±6.7 | 23.6±7.5 | 0.35±0.18 | 0.92±0.38 |
| Padovani/2002-1 | Italy | NINCDS-ADRDA | MMSE | 21 | 25 | 9/12 | 14/11 | 66.2±6.9 | 69.8±9.0 | 24.9±0.9 | 29.4±1.0 | 0.49±0.30 | 0.93±0.30 |
| Padovani/2002-2 | Italy | NINCDS-ADRDA | MMSE | 35 | 25 | 12/23 | 14/11 | 69.2±8.5 | 69.8±9.0 | 20.0±1.8 | 29.4±1.0 | 0.44±0.24 | 0.93±0.30 |
| Colciaghi/2002 | Italy | NINCDS-ADRDA | MMSE | 33 | 26 | 13/20 | 9/17 | 68.15±6.18 | 63.19±6.10 | 18.0±3.7 | 29.28±1.1 | 0.31±0.19 | 0.90±0.29 |
| Borroni/2002-1 | Italy | NINCDS-ADRDA | MMSE | 16 | 40 | 9/7 | 18/22 | 69.5±6.5 | 68.3±9 | 24.9±1.1 | 29.6±0.5 | 0.5±0.3 | 0.91±0.3 |
| Borroni/2002-2 | Italy | NINCDS-ADRDA | MMSE | 24 | 40 | 14/10 | 18/22 | 67.1±7.7 | 68.3±9 | 20.9±1.8 | 29.6±0.5 | 0.42±0.22 | 0.91±0.3 |
| Colciaghi/2004-1 | Italy | NINCDS-ADRDA | MMSE | 11 | 15 | 4/7 | 6/9 | 67.8±6.3 | 67.7±4.2 | 25.7±0.78 | 29.3±1.97 | 0.447±0.160 | 0.706±0.241 |
| Colciaghi/2004-2 | Italy | NINCDS-ADRDA | MMSE | 20 | 15 | 8/12 | 6/9 | 68.0±7.5 | 67.7±4.2 | 21.36±4.03 | 29.3±1.97 | 0.419±0.168 | 0.706±0.241 |
| Di Luca/2005 | Italy | NINCDS-ADRDA | MMSE | 37 | 25 | 17/20 | 11/14 | 67.3±6.8 | 66.5±3.8 | 24.9±4.4 | 29.6±1.0 | 0.44±0.18 | 0.61±0.22 |
| Sánchez/2006 | M´exico | NINCDS-ADRDA | MMSE | 26 | 46 | 17/9 | 15/31 | 60.96±12.85 | 67.73±9.46 | 14.64±5.31 | 28.33±1.96 | 0.3662±0.1891 | 0.6769±0.1021 |
| Liu/2007 | Taiwan | NINCDS-ADRDA | MMSE | 66 | 46 | 41/25 | 27/19 | 76.5±9.9 | 72.5±10.9 | 13.6±8.2 | 28.9±1.0 | 1.35±0.81 | 1.44±0.79 |
| Zainaghi/2007 | Brazil | NINCDS-ADRDA | MMSE | 23 | 29 | 8/15 | 7/22 | 74.4±9.0 | 70±5.8 | 18.6±5.1 | 29±1.1 | 1.01±0.21 | 1.24±0.21 |
| Hochstrasser/2012 | Austria | NINCDS-ADRDA | MMSE | 34 | 26 | 6/28 | 14/12 | 79±1.1 | 72±1.2 | 18.1±1.2 | 27.7±0.9 | 0.83±0.1 | 1.28±0.3 |
| Srisawat/2013 | Thailand | NINCDS-ADRDA | MMSE | 13 | 27 | 4/9 | 6/21 | 79.3±8.1 | 68.3±6.6 | 20.3±2.2 | 27.6±1.8 | 7.32±1.29 | 9.13±3.00 |
| Jelic/2013 | Sweden | NINCDS-ADRDA | MMSE | 30 | 23 | 12/19 | 9/14 | 67.0±9.9 | 68.2±9.6 | 25.4±2.77 | 28.65±1.37 | 5.9±1.37 | 10.3±2.82 |
| Sarno/2017 | Brazil | NINCDS-ADRDA | MMSE | 23 | 38 | 6/17 | 12/26 | 73.1±6.9 | 72.3±6.6 | 18±5.1 | 28.8±1.5 | 1.46±0.40 | 2.13±0.90 |
| Bermejo /2013 | Spain | NINCDS-ADRDA | MMSE | 45 | 28 | 16/29 | 11/17 | 79.7 ± 0.9 | 80.4 ± 2.1 | 18.8 ± 3.2 | 6.2 ± 2.5 | 1.09±0.21 | 1.8±0.37 |

Table S6: Study calculating the BACE-1 of Platelet.

| study | country | diagnostic  criteria | Cognitive scores | P  (n) | C  (n) | Sex (M/F) (P) | Sex (M/F) (C) | Age(P) | Age(C) | MMSE  (P) | MMSE  (C) | BACE-1 (P) | BACE-1 (C) |
| --- | --- | --- | --- | --- | --- | --- | --- | --- | --- | --- | --- | --- | --- |
| Di Luca/2005 | Italy | NINCDS-ADRDA | MMSE | 37 | 25 | 17/20 | 11/14 | 67.3±6.8 | 66.5±3.8 | 24.9±4.4 | 29.6±1.0 | 0.84±0.73 | 1.52±1.27 |
| Johnston/2008 | UK | NINCDS-ADRDA | MMSE | 86 | 115 | 25/61 | 42/73 | 80.0±6.6 | 79.1±8.2 | 17.7±5.0 | 29.0±1.0 | 0.178±0.053 | 0.152±0.04 |
| Gorham/2010 | Ireland | NINCDS-ADRDA | MMSE | 20 | 30 | 10/10 | 7/23 | 71.6±9.8 | 61.7±7.5 | 24±3 | NR | 285±52 | 279±57 |
| Decourt/2013 | USA | NINCDS-ADRDA | MMSE | 15 | 12 | 10/5 | 4/8 | 82.08±5.14 | 79.58±5.04 | 19.07±6.35 | 29.33±0.78 | 65.10±7.07 | 73.97±12.95 |
| Bermejo /2013 | Spain | NINCDS-ADRDA | MMSE | 45 | 28 | 16/29 | 11/17 | 79.7 ± 0.9 | 80.4 ± 2.1 | 18.8 ± 3.2 | 6.2 ± 2.5 | 0.92±0.13 | 0.43±0.02 |
| Marksteiner/2013 | Austria | NINCDS-ADRDA | MMSE | 68 | 33 | NR | NR | 79±1 | 71±1 | 21.0±0.5 | 28.2±9.4 | 6,054±1,307 | 2,283±273 |
| Sarno/2017 | Brazil | NINCDS-ADRDA | MMSE | 23 | 38 | 6/17 | 12/26 | 73.1±6.9 | 72.3±6.6 | 18±5.1 | 28.8±1.5 | 1.40±0.83 | 1.18±0.65 |
| Bram/2019 | Brazil | NINCDS-ADRDA | MMSE | 20 | 20 | 6/14 | 5/15 | 76.2±7.2 | 74.9±4.5 | 23.7±14.9 | 29.4±1.4 | 1.74±0.93 | 1.34±0.82 |

Table S7: Study calculating the PSEN-1 of Platelet.

| study | country | diagnostic  criteria | Cognitive scores | P  (n) | C  (n) | Sex (M/F) (P) | Sex (M/F) (C) | Age(P) | Age(C) | MMSE  (P) | MMSE  (C) | PSEN-1 (P) | PSEN-1 (C) |
| --- | --- | --- | --- | --- | --- | --- | --- | --- | --- | --- | --- | --- | --- |
| Sarno/2017 | Brazil | NINCDS-ADRDA | MMSE | 23 | 38 | 6/17 | 12/26 | 73.1±6.9 | 72.3±6.6 | 18±5.1 | 28.8±1.5 | 1.04±0.45 | 1.52±0.65 |
| Bram/2019 | Brazil | NINCDS-ADRDA | MMSE | 20 | 20 | 6/14 | 5/15 | 76.2±7.2 | 74.9±4.5 | 23.7±14.9 | 29.4±1.4 | 1.13±0.46 | 1.66±0.69 |
| Bermejo /2013 | Spain | NINCDS-ADRDA | MMSE | 45 | 28 | 16/29 | 11/17 | 79.7 ± 0.9 | 80.4 ± 2.1 | 18.8 ± 3.2 | 6.2 ± 2.5 | 1.10±0.07 | 0.43±0.02 |

Table S8: Study calculating the ADAM-10 of Platelet.

| study | country | diagnostic  criteria | Cognitive scores | P  (n) | C  (n) | Sex (M/F) (P) | Sex (M/F) (C) | Age(P) | Age(C) | MMSE  (P) | MMSE  (C) | ADAM-10/actin (P) | ADAM-10/actin (C) | ADAM-10 (P) | ADAM-10 (C) |
| --- | --- | --- | --- | --- | --- | --- | --- | --- | --- | --- | --- | --- | --- | --- | --- |
| Colciaghi/2004-1 | Italy | NINCDS-ADRDA | MMSE | 11 | 15 | 4/7 | 6/9 | 67.8±6.3 | 67.7±4.2 | 25.7±0.78 | 29.3±1.97 | 0.517±0.055 | 0.801±0.074 |  |  |
| Colciaghi/2004-2 | Italy | NINCDS-ADRDA | MMSE | 20 | 15 | 8/12 | 6/9 | 68.0±7.5 | 67.7±4.2 | 21.36±4.03 | 29.3±1.97 | 0.369±0.046 | 0.801±0.074 |  |  |
| Di Luca/2005 | Italy | NINCDS-ADRDA | MMSE | 37 | 25 | 17/20 | 11/14 | 67.3±6.8 | 66.5±3.8 | 24.9±4.4 | 29.6±1.0 | 0.43±0.23 | 0.79±0.49 |  |  |
| Zimmermann/2005 | Italy | NINCDS-ADRDA | MMSE | 25 | 25 | 18/7 | 15/10 | 75.0±5.0 | 73.5±5.6 | 21.6±5.7 | 29.2±0.8 | 0.39±0.05 | 0.82±0.06 |  |  |
| Gorham/2010 | Ireland | NINCDS-ADRDA | MMSE | 20 | 30 | 10/10 | 7/23 | 71.6±9.8 | 61.7±7.5 | 24±3 | NR |  |  | 36.2±5.8 | 37.6±6.9 |
| Manzine/2013-1 | Brazil | NINCDS-ADRDA | MMSE | 10 | 8 | 3/7 | 9/16 | 75.0±7.5 | 75.7±6.9 | 16.2±5.0 | 27.3±1.9 | 0.57±0.15 | 1.25±0.14 |  |  |
| Manzine/2013-2 | Brazil | NINCDS-ADRDA | MMSE | 11 | 8 | 4/7 | NR | 75.0±7.5 | NR | 16.2±5.0 | NR | 0.22±0.06 | 0.53±0.16 |  |  |
| Manzine/2013-3 | Brazil | NINCDS-ADRDA | MMSE | 9 | 9 | 3/6 | NR | 75.0±7.5 | NR | 16.2±5.0 | NR | 0.14±0.05 | 0.46±0.21 |  |  |
| Manzine/2013 | Brazil | NINCDS-ADRDA | MMSE | 30 | 25 | 30/0 | 25/0 | ≥70 | ≥70 | 13.5±9.6 | 28.5±1.75 | 0.31±0.20 | 0.66±0.35 |  |  |
| Sarno/2017 | Brazil | NINCDS-ADRDA | MMSE | 23 | 38 | 6/17 | 12/26 | 73.1±6.9 | 72.3±6.6 | 18±5.1 | 28.8±1.5 |  |  | 0.67±0.45 | 1.61±0.67 |
| Bram/2019 | Brazil | NINCDS-ADRDA | MMSE | 20 | 20 | 6/14 | 5/15 | 76.2±7.2 | 74.9±4.5 | 23.7±14.9 | 29.4±1.4 |  |  | 0.79±0.43 | 1.66±0.57 |
| Bermejo /2013 | Spain | NINCDS-ADRDA | MMSE | 45 | 28 | 16/29 | 11/17 | 79.7 ± 0.9 | 80.4 ± 2.1 | 18.8 ± 3.2 | 6.2 ± 2.5 | 1.11±0.42 | 0.46±0.16 |  |  |

Table S9: Study calculating the Ca^2+^ of Platelet.

| study | country | diagnostic  criteria | Cognitive scores | P  (n) | C  (n) | Sex (M/F) (P) | Sex (M/F) (C) | Age(P) | Age(C) | MMSE  (P) | MMSE  (C) | Ca^2+^ (P) | Ca^2+^ (C) |
| --- | --- | --- | --- | --- | --- | --- | --- | --- | --- | --- | --- | --- | --- |
| Le/1993-1 | France | DSM-Ⅲ | NR | 3 | 6 | 3/0 | 6/0 | 83.6±1.9 | 78.3±3.5 | NR | NR | 222±44 | 226±20 |
| Le/1993-2 | France | DSM-Ⅲ | NR | 19 | 8 | 0/19 | 0/8 | 85.4±1.1 | 81.6±2.7 | NR | NR | 224±14 | 76±21 |
| Davies/1993 | USA | DSM-Ⅲ | NR | 21 | 17 | NR | 10/7 | 70.5±5.4 | 60.8±8.2 | NR | NR | 75.5±5.7 | 76.7±2.5 |
| Davies/1997 | USA | DSM-Ⅲ | NR | 27 | 17 | 23/4 | 10/7 | 70.5±5.4 | 60.8±8.2 | NR | NR | 82.1±3.3 | 81.5±3.1 |
| Fernandes/1999 | Portugal | NINCDS-ADRDA | MMSE | 24 | 26 | 11/13 | 10/16 | 66.88+10.36 | 63.23+9.60 | 10.51+7.47 | 30 | 66.4±17.8 | 63.8±19.3 |
| Rˇ ı´pova´-2000 | Czech Republic | NINCDS-ADRDA | NR | 22 | 16 | 14/8 | 9/7 | 69.5±1.6 | 31.9±2.4 | NR | NR | 73.9±4.4 | 114.8±5.6 |
| Rˇ ı´pova´-2004 | Czech Republic | NINCDS-ADRDA | MMSE | 21 | 17 | 13/8 | 7/10 | 68.3±1.7 | 70.4±1.1 | NR | NR | 81±5.9 | 122±4.7 |
| Vignini/2007 | Italy | NINCDS-ADRDA | NR | 100 | 50 | NR | NR | 68±7 | 65±9 | NR | NR | 214.93±32.65 | 157.97±14.73 |
| Vignini/2013-1 | Italy | NINCDS-ADRDA | MMSE | 40 | 25 | 40/0 | 25/0 | 66±5 | 63±4 | 17.7±3.2 | 29.1±1.7 | 222.56±10.14 | 203.55±16.21 |
| Vignini/2013-2 | Italy | NINCDS-ADRDA | MMSE | 60 | 25 | 0/60 | 0/25 | 72±7 | 70±8 | 18.2±1.9 | 28.9±1.4 | 169.07±14.10 | 148.97±8.00 |

Table S10: Study calculating the PLA2 of Platelet.

| study | country | diagnostic  criteria | Cognitive scores | P  (n) | C  (n) | Sex (M/F) (P) | Sex (M/F) (C) | Age(P) | Age(C) | MMSE  (P) | MMSE  (C) | PLA_2_(pmol/ mg Protein/min) (P) | PLA_2_(pmol/ mg Protein/min) (C) |
| --- | --- | --- | --- | --- | --- | --- | --- | --- | --- | --- | --- | --- | --- |
| Gattaz/1996 | Germany | NINCDS-ADRDA | MMSE | 16 | 13 | 4/8 | 9/4 | 70.2+11.3 | 62.6+9.7 | NR | NR | 14.3±4.6 | 19.4±6.4 |
| Gattaz/2004 | Brazil | NINCDS-ADRDA | MMSE | 21 | 17 | 6/15 | 4/13 | 75.1±6.9 | 72.5±4.6 | 14.29±8.96 | 27.94±2.07 | 18.8±4.0 | 28.3±7.8 |
| Krzystanek/2007 | Poland | NINCDS-ADRDA | MMSE | 37 | 27 | 10/27 | 15/12 | 73±6.45 | 71.85±4.91 | 18.6±4.6 | NR | 998±386 | 387±20 |
| Gattaz/2013 | Brazil | NINCDS-ADRDA | MMSE | 44 | 66 | 11/33 | 12/54 | 74.8±6.5 | 67.4±5.4 | 18.6±5.1 | 28.2±3.8 | 26.35±8.95 | 25.27±8.73 |

Table S11: Study calculating the NO of Platelet.

| study | country | diagnostic  criteria | Cognitive scores | P  (n) | C  (n) | Sex (M/F) (P) | Sex (M/F) (C) | Age(P) | Age(C) | MMSE  (P) | MMSE  (C) | NO (nmol/mg protein) (P) | NO (nmol/mg protein) (C) | ONOO^-^ (P) | ONOO^-^ (C) |
| --- | --- | --- | --- | --- | --- | --- | --- | --- | --- | --- | --- | --- | --- | --- | --- |
| Vignini/2007 | Italy | NINCDS-ADRDA | NR | 100 | 50 | NR | NR | 68±7 | 65±9 | NR | NR | 15.40±1.41 | 7.73±0.61 | 24.93±2.53 | 10.04±1.23 |
| Vignini/2013-1 | Italy | NINCDS-ADRDA | MMSE | 60 | 25 | 0/60 | 0/25 | 72±7 | 70±8 | 18.2±1.9 | 28.9±1.4 | 15.38±1.29 | 9.71±1.12 | 18.53±1.38 | 8.67±0.80 |
| Vignini/2013-2 | Italy | NINCDS-ADRDA | MMSE | 40 | 25 | 40/0 | 25/0 | 66±5 | 63±4 | 17.7±3.2 | 29.1±1.7 | 20.11±2.71 | 5.55±0.97 | 29.05±2.36 | 12.84±1.15 |
| Yu/2009 | China | NINCDS-ADRDA | MMSE | 40 | 42 | 20/20 | 13/29 | 72±8 | 69±4 | 14±4 | 27±2 | 0.743±0.164 | 0.874±0.378 |  |  |

Table S12: Study calculating the Platelet membrane fluidity

| study | country | Diagnostic  criteria | Cognitive scores | P  (n) | C (n) | Sex  (P) (M/F) | Sex  (C) (M/F) | Age (P) | Age (C) | MMSE (P) | MMSE (C) | DPH  (Steady-State Anisotropy) | | DPH (Fluorescence Lifetime | | TMA-DPH (Steady-State Anisotropy) | | | | TMA-DPH (Fluorescence Lifetime) | | |  |
| --- | --- | --- | --- | --- | --- | --- | --- | --- | --- | --- | --- | --- | --- | --- | --- | --- | --- | --- | --- | --- | --- | --- | --- |
|  |  |  |  |  |  |  |  |  |  |  |  | (P) | (C) | (P) | (C) | (P) | | (C) | | (P) | | (C) |  |
| Vignini/2007 | Italy | NINCDS-ADRDA | NR | 100 | 50 | NR | NR | 68±7 | 65±9 | NR | NR | 0.24±0.04 | 0.21±0.02 |  |  |  | |  | |  | |  |  |
| Vignini/2013-1 | Italy | NINCDS-ADRDA | MMSE | 40 | 25 | 40/0 | 25/0 | 66±5 | 63±4 | 17.7±3.2 | 29.1±1.7 | 0.26±0.01 | 0.21±0.01 |  |  |  | |  | |  | |  |  |
| Vignini/2013-2 | Italy | NINCDS-ADRDA | MMSE | 60 | 25 | 0/60 | 0/25 | 72±7 | 70±8 | 18.2±1.9 | 28.9±1.4 | 0.23±0.01 | 0.23±0.02 |  |  |  | |  | |  | |  |  |
| Cohen/1987 | USA | NINCDS-ADRDA | NR | 10 | 10 | 2/8 | 2/8 | 67.5±7.3 | 67.6±7.9 | NR | NR | 0.19±0.01 | 0.20±0.00 |  |  |  | |  | |  | |  |  |
| Zubenko/1987 | USA | NINCDS-ADRDA | NR | 24 | 36 | 9/15 | 20/16 | 47-87 | 45-85 | NR | NR | 0.19±0.01 | 0.20±0.01 | 8.5±0.2 | 8.5±0.3 | 0.25±0.00 | | 0.26±0.00 | | 4.7±0.2 | | 4.7±0.3 |  |
| Zubenko/1987 | USA | NINCDS-ADRDA | MMSE | 51 | 50 | 17/34 | 18/32 | 68.6±8.0 | 66.7±9.4 | ≥27 | 16.8±4.7 | 0.19±0.01 | 0.20±0.01 | 8.5±0.3 | 8.5±0.3 | 0.25±0.00 | | 0.25±0.00 | | 4.7±0.3 | | 4.7±0.3 |  |
| Zubenko/1987 | USA | NINCDS-ADRDA | MMSE | 5 | 5 | 3/2 | 3/2 | 68.0±7.1 | 70.0±8.0 | 17.6±5.0 | 28.8±2.2 | 0.19±0.00 | 0.20±0.01 | 8.4±0.2 | 8.4±0.1 | 0.25±0.00 | | 0.25±0.00 | | 4.6±0.2 | | 4.5±0.3 |  |
| Zubenko/1987 | USA | NINCDS-ADRDA | MMSE | 38 | 34 | 27/11 | 13/21 | 49.6±10.9 | 51.0±7.5 | NR | NR | 0.20±0.01 | 0.20±0.00 |  |  |  | |  | |  | |  |  |
| Zubenko/1987 | USA | NINCDS-ADRDA | MMSE | 6 | 6 | 2/4 | 3/3 | 63.5±5.9 | 63.5±5.9 | NR | NR | 0.18±0.01 | 0.20±0.01 | 8.6±0.2 | 8.6±0.2 |  |  | |  | |  | | |

Table S13: Study calculating the adenosine A_2_ receptor of Platelet.

| study | country | diagnostic  criteria | Cognitive scores | P  (n) | C  (n) | Sex (M/F) (P) | Sex (M/F) (C) | Age(P) | Age(C) | MMSE  (P) | MMSE  (C) | adenosine A_2_ receptor (P) | adenosine A_2_ receptor (C) |
| --- | --- | --- | --- | --- | --- | --- | --- | --- | --- | --- | --- | --- | --- |
| Adunsky/1989 | Israel | NINCDS-ADRDA | NR | 75 | 51 | 34/41 | 28/31 | 73.1±0.8 | 72.4±0.8 | NR | NR | 2.18±0.15 | 1.73±0.13 |
| Merighi/2021 | Italy | DSM-5 | MMSE | 26 | 26 | NR | NR | NR | NR | NR | NR | 214±17 | 95±4 |
| Mukaetova /2012 | UK | NINCDS-ADRDA | MMSE | 25 | 26 | 15/10 | 8/18 | 78.08±1.00 | 70.81±1.98 | 20.68±1.28 | 28.15±0.36 | 55.40±3.54 | 53.12±3.32 |

Table S14: Study calculating the Na^+^-K^+^ -ATPase of Platelet.

| study | country | diagnostic  criteria | Cognitive scores | P  (n) | C  (n) | Sex (M/F) (P) | Sex (M/F) (C) | Age(P) | Age(C) | MMSE  (P) | MMSE  (C) | Na^+^-K^+^-ATPase (P) | Na^+^-K^+^-ATPase (C) |
| --- | --- | --- | --- | --- | --- | --- | --- | --- | --- | --- | --- | --- | --- |
| Vignini/2007 | Italy | NINCDS-ADRDA |  | 100 | 50 |  |  | 68±7 | 65±9 |  |  | 5.42±0.54 | 9.17±0.76 |
| Vignini/2013-1 | Italy | NINCDS-ADRDA | MMSE | 40 | 25 | 40/0 | 25/0 | 66±5 | 63±4 | 17.7±3.2 | 29.1±1.7 | 3.33±0.25 | 6.33±0.58 |
| Vignini/2013-2 | Italy | NINCDS-ADRDA | MMSE | 60 | 25 | 0/60 | 0/25 | 72±7 | 70±8 | 18.2±1.9 | 28.9±1.4 | 7.70±0.68 | 16.08±1.49 |

Table S15: Study calculating the MAO-B of Platelet.

| study | country | diagnostic  criteria | Cognitive scores | P  (n) | C  (n) | Sex (M/F) (P) | Sex (M/F) (C) | Age(P) | Age(C) | MMSE  (P) | MMSE  (C) | MAO-B (P) | MAO-B (C) |
| --- | --- | --- | --- | --- | --- | --- | --- | --- | --- | --- | --- | --- | --- |
| Adolfsson/1980 | Sweden |  |  | 11 | 11 | 6/5 | 6/5 | 65±0.5 | 65±0.5 |  |  | 0.74±0.07 | 0.53±0.05 |
| Smith/1982 | USA |  |  | 12 | 16 |  |  |  |  |  |  | 61.2±4.8 | 59.9±6.6 |
| Danielczyk/1988 | Austria |  | MMSE | 21 | 21 | 4/17 | 4/17 | 76±10.4 | 73±10.6 | 8.4+9.0 |  | 0.269±0.266 | 0.206±0.202 |
| Schneider/1988 | USA | NINCDS-ADRDA | MMSE | 15 | 14 | 4/11 | 5/9 | 75.2±12.3 | 73.4±10.1 | 13.6±8.9 | 29.8±0.7 | 7.27±3.85 | 4.98±1.64 |
| Bonuccelli/1990 | Italy | NINCDS-ADRDA | CDR | 32 | 20 | 14/18 | 9/11 | 67.0+7.5 | 67.4±5.9 |  |  | 40.1±10.3 | 21.0±6.9 |
| Regland/1991 | Sweden |  |  | 14 | 47 | 5/9 |  | 80±4 | 72±5 |  |  | 1.5±0.6 | 1.02±0.43 |
| Parnetti/1992 | Italy | NINCDS-ADRDA | MMSE | 12 | 26 | 3/9 | 14/12 | 62.7±1.2 | 72.1±1.4 | 16.5+3.8 |  | 257±34 | 312±23 |
| Ahlskog/1996 | USA | NINCDS-ADRDA |  | 12 | 15 | 4/8 | 3/12 | 73.8(57-88) | 61.4(46-85) |  |  | 1.78±0.87 | 2.01±0.73 |
| Fitzgerald/1996 | Ireland |  |  | 16 | 9 |  |  |  |  |  |  | 0.310±0.056 | 0.266±0.013 |
| Bongioanni/1996 | Italy | NINCDS-ADRDA | CDR | 35 | 35 | 20/15 | 19/16 |  |  |  |  | 43.40±11.37 | 27.57±7.77 |
| Bongioanni/1997 | Italy | NINCDS-ADRDA |  | 50 | 50 | 28/22 | 26/24 | 67.6+14.1 | 64.1±12.9 |  |  | 44.42±1.49 | 24.53±1.11 |
| Soto/1999 | Spain | NINCDS-ADRDA | MMSE | 20 | 17 | 3/17 | 4/13 | 77±2.0 | 79±1.3 | 5±1 | 31±1 | 7.73±0.48 | 6.35±0.47 |
| Muck-Seler/2009 | Croatia | NINCDS-ADRDA | MMSE | 74 | 49 | 0/74 | 0/49 | 76.9±9.9 | 73.7±8.8 | 11.7±8.1 | 28.56±1.94 | 29.11±11.69 | 30.64±12.18 |
| Adolfsson/1980 | Sweden |  |  | 11 | 11 | 6/5 | 6/5 | 65±0.5 | 65±0.5 |  |  | 0.74±0.07 | 0.53±0.05 |
| Zainaghi/2007 | Brazil | NINCDS-ADRDA | MMSE | 23 | 29 | 8/15 | 7/22 | 74.4±9.0 | 70±5.8 | 18.6±5.1 | 29±1.1 | 1.01±0.21 | 1.24±0.21 |
| Hochstrasser/2012 | Austria | NINCDS-ADRDA | MMSE | 34 | 26 | 6/28 | 14/12 | 79±1.1 | 72±1.2 | 18.1±1.2 | 27.7±0.9 | 0.83±0.1 | 1.28±0.3 |
