# Supplementary material for: Meta-analysis and systematic review of peripheral platelet-associated biomarkers to explore the pathophysiology of alzheimer's disease
Source: BMC Neurol. 2023 Feb 11;23:66. doi: 10.1186/s12883-023-03099-5 (PMC9921402; doi:10.1186/s12883-023-03099-5)
Supplement: Supplementary file 4 — Additional file 4: Table S16. Study calculating the coated-platelet. Table S17. Study calculating the Platelet membrane drug receptor (5-HT). Table S18. Study calculating the PAF of Platelet. Table S19. Study calculating the immunoglobulin of Platelet. Table S20. Study calculating the BACE (36 kDa/BACE 57 kDa) of Platelet. Table S21. Study calculating the APP of Platelet. Table S22. Study calculating the MMP-9 of Platelet. Table S23. Study calculating the MMP-2 of Platelet. Table S24. Study calculating the PKC of Platelet. Table S25. Study calculating the Lipid composition of platelet membrane. Table S26. Study calculating the PDGF of Platelet. Table S27. Study calculating the PECAM-1 of Platelet. Table S28. Study calculating the PST of Platelet. Table S29. Study calculating the CLEC-2 of Platelet. Table S30. Study calculating the EVs of Platelet. Table S31. Study calculating the GSKβ of Platelet. [file 12883_2023_3099_MOESM4_ESM.docx]

Table S16: Study calculating the coated-platelet.

| study | country | diagnostic criteria | Cognitive scores | P (n) | C (n) | Sex (P) (M/F) | Sex (C) (M/F) | Age (P) | Age (C) | MMSE (P) | MMSE (C) | coated-platelet (%) (P) | coated-platelet (%) (C) |
| --- | --- | --- | --- | --- | --- | --- | --- | --- | --- | --- | --- | --- | --- |
| Prodan/2006 | USA | NINCDS-ADRDA | MMSE | 10 | 19 | NR | NR | NR | NR | NR | NR | 41.0±9.9 | 28.7±10.7 |
| Prodan/2009 | USA | NINCDS-ADRDA | MMSE | 40 | 40 | NR | NR | 74.5±7.5 | 72.1±9.9 | 19.2±4.9 | 29.9±0.3 | 34.3±12.9 | 32.9±13.2 |

Table S17: Study calculating the Platelet membrane drug receptor (5-HT)

| study | country | diagnostic  criteria | Cognitive scores | Drug | P(n) | C(n) | Sex (P) (M/F) | Sex (C)  (M/F) | Age (P) | Age (C) | MMSE (P) | MMSE (C) | Bmax (P) (fmol/mg prot) | Bmax (C)  (fmol/mg prot) | Kd (P)  (nM) | Kd (C)  (nM) |
| --- | --- | --- | --- | --- | --- | --- | --- | --- | --- | --- | --- | --- | --- | --- | --- | --- |
| Bongioanni/1997 | Italy | NINCDS-ADRDA | CDR | BDZ | 45 | 45 | 25/20 | 24/21 | 67.9±14.6 | 63.2±12.7 |  |  | 2427±678 | 3267±872 | 3.2±0.8 | 3.7±0.9 |
| Nemeroff/1988 | England | DSM-Ⅲ |  | Imipramine | 13 | 18 | 4/9 | 11/7 | 72.9 ±1.8 | 66.8±1.1 |  |  | 885 ±52 | 943 ±46 | 0.86±0.05 | 0.99±0.06 |
| Galzin/1989 | France | NINCDS-ADRDA | MMSE | Imipramine | 14 | 15 | 3/11 | 3/12 | 82.1±6.6 | 81.5±2.0 | 7.1±6.3 | 25.2±2.3 | 810±208 | 702±162 | 0.77±0.55 | 0.57±0.21 |
| Andersson/1991 | Sweden | NINCDS-ADRDA |  | Paroxetine | 10 | 17 | 10/0 | 17/0 | 64 (48 - 79) | 67 (48 - 92) |  |  | 1340±40 | 1330±80 | 0.041(0.028-0.061) | 0.048(0.024-0.052) |
| Andersson/1991 | Sweden | NINCDS-ADRDA |  | Paroxetine | 30 | 13 | 0/30 | 0/13 |  |  |  |  | 1290±50 | 1150±80 | 0.040(0.027-0.058) | 0.044(0.031-0.062) |
| Spigset/2000 | Norway | DSMIV | MMSE | 5-HT2A | 38 | 40 | 9/29 | 9/31 | 79.6±5.2 | 72.6±6.1 | 13.4±5.9 |  | 37.5±14.1 | 32.6±8.8 | 1.81(1.39-2.16) | 1.30(0.94-1.59) |

Table S18: Study calculating the PAF of Platelet.

| study | country | diagnostic criteria | Cognitive scores | P (n) | C (n) | Sex (P) (M/F) | Sex (C) (M/F) | Age (P) | Age(C) | MMSE (P) | MMSE (C) | PAF (P) | PAF (C) |
| --- | --- | --- | --- | --- | --- | --- | --- | --- | --- | --- | --- | --- | --- |
| Hershkowitz/1996 | Israel | NINCDS-ADRDA | MMSE | 22 | 22 | 11/11 | 10/12 | 77.1±1.0 | 74.9±1.4 | 11.5±1.1 | 27.2±0.4 | 133.3±8.4 | 202.3±11.7 |
| Bacchetti/2015 | Italy | NINCDS-ADRDA | MMSE | 49 | 34 | 20/29 | 15/19 | 73.3±6.4 | 74.4±9.2 | 21.1±5.4 | NR | 21.8±5.1 | 15.2±4.3 |

Table S19: Study calculating the immunoglobulin of Platelet

| study | country | Diagnostic  criteria | Cognitive scores | P (n) | C (n) | Sex (P) (M/F) | Sex (C) (M/F) | Age (P) | Age (C) | MMSE (P) | MMSE (C) | immunoglobulin (P) | immunoglobulin (C) |
| --- | --- | --- | --- | --- | --- | --- | --- | --- | --- | --- | --- | --- | --- |
| Mukaetova-Ladinska/2012 | UK | NINCDS-ADRDA | MMSE | 27 | 26 | 15/12 | 8/18 | 78.08±1.00 | 70.81±1.98 | 20.68±1.28 | 28.15±0.36 | 5.50±0.31 | 4.80±0.18 |
| Mukaetova-Ladinska/2012 | UK | NINCDS-ADRDA | MMSE | 25 | 24 | 15/10 | 8/16 | 77 (66–85) | 72.5 (52–87) | 23 (4–28) | 29 (24–30) | 5.61 (2.75–10.71) | 5.03 (2.98–5.98) |

Table S20: Study calculating the BACE (36 kDa/BACE 57 kDa) of Platelet

| study | country | Diagnostic  criteria | Cognitive scores | P (n) | C (n) | Sex (P) (M/F) | Sex (C) (M/F) | Age (P) | Age (C) | MMSE (P) | MMSE (C) | BACE (36 kDa/  BACE 57 kDa) (P) | BACE (36 kDa/  BACE 57 kDa) (C) |
| --- | --- | --- | --- | --- | --- | --- | --- | --- | --- | --- | --- | --- | --- |
| Colciaghi/2004-1 | Italy | NINCDS-ADRDA | MMSE | 11 | 15 | 4/7 | 6/9 | 67.8±6.3 | 67.7±4.2 | 25.7±0.78 | 29.3±1.97 | 0.667±0.165 | 1.469±0.201 |
| Colciaghi/2004-2 | Italy | NINCDS-ADRDA | MMSE | 20 | 15 | 8/12 | 6/9 | 68.0±7.5 | 67.7±4.2 | 21.36±4.03 | 29.3±1.97 | 0.735±0.104 | 1.469±0.201 |

Table S21: Study calculating the APP of Platelet

| study | country | Diagnostic  criteria | Cognitive scores | P (n) | C (n) | Sex (P) (M/F) | Sex (C) (M/F) | Age (P) | Age (C) | MMSE (P) | MMSE (C) | APP-C  (P) | APP-C  (C) | APP-N  (P) | APP-N  (C) |
| --- | --- | --- | --- | --- | --- | --- | --- | --- | --- | --- | --- | --- | --- | --- | --- |
| Mukaetova-Ladinska/2012 | UK | NINCDS-ADRDA | MMSE | 25 | 26 | 15/10 | 8/18 | 78.08 ± 1.00 | 70.81 ± 1.98 | 20.68 ± 1.28 | 28.15 ± 0.36 | 24.68±1.40 | 24.02±1.38 | 51.12±4.84 | 45.42±3.25 |

Table S22: Study calculating the MMP-9 of Platelet

| study | country | Diagnostic  criteria | Cognitive scores | P (n) | C (n) | Sex (P) (M/F) | Sex (C) (M/F) | Age (P) | Age (C) | MMSE (P) | MMSE (C) | MMP-9 (ng/mg)  (P) | MMP-9 (ng/mg)  (C) |
| --- | --- | --- | --- | --- | --- | --- | --- | --- | --- | --- | --- | --- | --- |
| Hochstrasser/2012 | Austria | NINCDS-ADRDA | MMSE | 34 | 26 | 6/28 | 14/12 | 79±1.1 | 72±1.2 | 18.1±1.2 | 27.7±0.9 | 95.6±18.2 | 68.4±20.1 |

Table S23: Study calculating the MMP-2 of Platelet

| study | country | Diagnostic  criteria | Cognitive scores | P (n) | C (n) | Sex (P) (M/F) | Sex (C) (M/F) | Age (P) | Age (C) | MMSE (P) | MMSE (C) | MMP-2 (ng/mg)  (P) | MMP-2 (ng/mg)  (C) |
| --- | --- | --- | --- | --- | --- | --- | --- | --- | --- | --- | --- | --- | --- |
| Hochstrasser/2012 | Austria | NINCDS-ADRDA | MMSE | 34 | 26 | 6/28 | 14/12 | 79±1.1 | 72±1.2 | 18.1±1.2 | 27.7±0.9 | 991±162 | 1705±252 |

Table S24: Study calculating the PKC of Platelet

| study | country | Diagnostic  criteria | Cognitive scores | P (n) | C (n) | Sex (P) (M/F) | Sex (C) (M/F) | Age (P) | Age (C) | MMSE (P) | MMSE (C) | PKC (P)  (pmol phosphate/mg/min) | PKC (C)  (pmol phosphate/mg/min) |
| --- | --- | --- | --- | --- | --- | --- | --- | --- | --- | --- | --- | --- | --- |
| BOSMAN/1992 | Netherlands | DSM-III | NR | 11 | 12 | NR | NR | 80±6 | 81±5 | NR | NR | 69±13 | 132±21 |
| Lanius/1997 | Canada | NINCDS-ADRDA | MMSE | 9 | 7 | NR | NR | 74.9±8.4 | 71.3±8.4 | NR | NR | 264±23 | 254±19 |

Table S25: Study calculating the Lipid composition of platelet membrane

| study | country | Diagnostic  criteria | Cognitive scores | P (n) | C (n) | Sex (P) (M/F) | Sex (C) (M/F) | Age (P) | Age (C) | TC (P) | TC (C) | FA (P) | FA (C) | Lipid  (P) | Lipid  (C) | | C/P  (P) | | C/P  (C) | | PLC  (P) | | PLC  (C) |
| --- | --- | --- | --- | --- | --- | --- | --- | --- | --- | --- | --- | --- | --- | --- | --- | --- | --- | --- | --- | --- | --- | --- | --- |
| BOSMAN/1992 | Netherlands | DSM-III | NR | 11 | 12 |  |  | 80±6 | 81±5 | 69±13 | 132±21 | 258±47 | 317±34 | 421±86 | 356±38 | | 0.61±0.16 | | 0.89±0.15 | |  | |  |
| Cohen/1987 | USA | NINCDS-ADRDA | NR | 10 | 10 | 2/8 | 2/8 | 67.5±7.3 | 67.6±7.9 |  |  | 134±24.5 | 140±26.3 |  |  | |  | |  | | 14.40±3.37 | | 14.00±2.83 |
| Matsushima/1995 | Japan | NINCDS-ADRDA | NR | 10 | 10 |  |  | 82.2+1.5 | 78.0+2.7 | 264±23 | 254±19 |  |  |  |  |  | |  | |  | |  | |

Table S26: Study calculating the PDGF of Platelet

| study | country | Diagnostic  criteria | Cognitive scores | P (n) | C (n) | Sex (P) (M/F) | Sex (C) (M/F) | Age (P) | Age (C) | MMSE (P) | MMSE (C) | PDGF (pg/mg)  (P) | PDGF (pg/mg)  (C) |
| --- | --- | --- | --- | --- | --- | --- | --- | --- | --- | --- | --- | --- | --- |
| Hochstrasser/2012 | Austria | NINCDS-ADRDA | MMSE | 34 | 26 | 6/28 | 14/12 | 79±1.1 | 72±1.2 | 18.1±1.2 | 27.7±0.9 | 2093±267 | 1508±147 |

Table S27: Study calculating the PECAM-1 of Platelet

| study | country | Diagnostic  criteria | Cognitive scores | P (n) | C (n) | Sex (P) (M/F) | Sex (C) (M/F) | Age (P) | Age (C) | MMSE (P) | MMSE (C) | PECAM-1(ng/mg)  (P) | PECAM-1(ng/mg) (C) |
| --- | --- | --- | --- | --- | --- | --- | --- | --- | --- | --- | --- | --- | --- |
| Hochstrasser/2012 | Austria | NINCDS-ADRDA | MMSE | 34 | 26 | 6/28 | 14/12 | 79±1.1 | 72±1.2 | 18.1±1.2 | 27.7±0.9 | 345±33 | 337±39 |

Table S28: Study calculating the PST of Platelet

| study | country | Diagnostic  criteria | Cognitive scores | P (n) | C (n) | Sex (P) (M/F) | Sex (C) (M/F) | Age (P) | Age (C) | MMSE (P) | MMSE (C) | PST (pmols/mg prot./10min) (P) | PST (pmols/mg prot./10min) (C) |
| --- | --- | --- | --- | --- | --- | --- | --- | --- | --- | --- | --- | --- | --- |
| Bongioanni/1996 | Italy | NINCDS-ADRDA | CDR | 35 | 35 | 20/15 | 19/16 | NR | NR | NR | NR | 181.75+29.02 | 93.77+30.28 |

Table S29: Study calculating the CLEC-2 of Platelet

| study | country | Diagnostic  criteria | Cognitive scores | P (n) | C (n) | Sex (P) (M/F) | Sex (C) (M/F) | Age (P) | Age (C) | MMSE (P) | MMSE (C) | CLEC-2 (pg/mL) (P) | CLEC-2 (pg/mL) (C) |
| --- | --- | --- | --- | --- | --- | --- | --- | --- | --- | --- | --- | --- | --- |
| Wang/2019 | China | NINCDS-ADRDA | MMSE | 110 | 110 | 40/70 | 38/72 | 76.1±3.9 | 75.8±3.8 | 14.4±2.2 | 27.8±1.5 | 0.184±0.024 | 0.327±0.138 |

Table S30: Study calculating the EVs of Platelet

| study | country | Diagnostic  criteria | Cognitive scores | P (n) | C (n) | Sex (P) (M/F) | Sex (C) (M/F) | Age (P) | Age (C) | EVs (nm) (P) | EVs (nm) (C) |
| --- | --- | --- | --- | --- | --- | --- | --- | --- | --- | --- | --- |
| Odaka/2021 | Japan | NINCDS-ADRDA | NR | 20 | 29 | 14/16 | 19/10 | 62.00 (48.00, 84.00) | 34.00 (18.00, 69.00) | 197±10.2 | 159±1.11 |

Table S31: Study calculating the GSKβ of Platelet

| study | country | Diagnostic  criteria | Cognitive scores | P (n) | C (n) | Sex (P) (M/F) | Sex (C) (M/F) | Age (P) | Age (C) | MMSE (P) | MMSE (C) | Total GSK3β (P) | Total  GSK3β(C) | p-GSK3β  (P) | p-GSK3β  (C) | GSK3β  ratio(P) | | GSK3β ratio(C) | |
| --- | --- | --- | --- | --- | --- | --- | --- | --- | --- | --- | --- | --- | --- | --- | --- | --- | --- | --- | --- |
| Forlenza/2011 | Brazil | NINCDS-ADRDA | MMSE | 24 | 23 | 10/14 | 7/16 | 76.1±4.2 | 71.2±5.3 | 18.5±4.1 | 28.0±3.3 | 0.97±0.59 | 0.94±0.45 | 0.46±0.24 | 0.62±0.36 | 0.53±0.19 | | 0.67±0.22 | |
| Pláteník/2014 | Czech Republic | NINCDS-ADRDA | MMSE | 85 | 96 | 34/51 | 66/30 | 75.6±7.7 | 47.8±16.1 | 18.8±6.9 | 29.8±0.6 |  |  | 2.635±1.725 | 2.805±1.395 |  |  | |  |
