# Supplementary material for: Meta-analysis and systematic review of peripheral platelet-associated biomarkers to explore the pathophysiology of alzheimer's disease
Source: BMC Neurol. 2023 Feb 11;23:66. doi: 10.1186/s12883-023-03099-5 (PMC9921402; doi:10.1186/s12883-023-03099-5)
Supplement: Supplementary file 5 — Additional file 5: Table 32. quality assessment of NOS. [file 12883_2023_3099_MOESM5_ESM.docx]

| Table 32: quality assessment of NOS | | | | | |
| --- | --- | --- | --- | --- | --- |
| study | Country | selection☆ | comparation☆ | exposure☆ | total☆ |
| Andersson/1991 | Sweden | 3 | 2 | 3 | 8 |
| Koren/1993 | Israel | 3 | 2 | 3 | 8 |
| Inestrosa/1993 | Chile | 2 | 0 | 3 | 5 |
| Kumar/1995 | USA | 3 | 2 | 3 | 8 |
| Spigset/2000 | Norway | 4 | 2 | 3 | 9 |
| kumar/2008 | USA | 4 | 2 | 3 | 9 |
| Muck-Seler/2009 | Croatia | 3 | 2 | 3 | 8 |
| Hochstrasser/2012 | Austria | 2 | 0 | 3 | 5 |
| Neumann/2011 | Chile | 4 | 2 | 3 | 9 |
| Slachevsky/2016 | Chile | 3 | 2 | 3 | 8 |
| Guzm´an-Mart´ınez/2019 | Chile | 2 | 0 | 3 | 5 |
| Di Luca/1996 | Italy | 3 | 2 | 3 | 8 |
| Rosenberg/1997 | America | 2 | 0 | 3 | 5 |
| Di Luca/1998 | Italy | 3 | 2 | 3 | 8 |
| Baskin/2000 | Dallas | 2 | 1 | 3 | 6 |
| Borroni/2001 | Italy | 2 | 2 | 3 | 7 |
| Padovani/2001 | Italy | 3 | 2 | 3 | 8 |
| Padovani/2002 | Italy | 3 | 2 | 3 | 8 |
| Colciaghi/2002 | Italy | 3 | 2 | 3 | 8 |
| Borroni/2002 | Italy | 3 | 2 | 3 | 8 |
| Colciaghi/2004 | Italy | 2 | 2 | 3 | 7 |
| Di Luca/2005 | Italy | 2 | 2 | 3 | 7 |
| Sánchez-González/2006 | M´exico | 2 | 0 | 3 | 5 |
| Liu/2007 | Taiwan | 2 | 2 | 3 | 7 |
| Zainaghi/2007 | Brazil | 2 | 0 | 3 | 5 |
| Srisawat/2013 | Thailand | 3 | 2 | 3 | 8 |
| Jelic/2013 | Sweden | 2 | 2 | 3 | 7 |
| Sarno/2017 | Brazil | 3 | 2 | 3 | 8 |
| Bermejo-Besco´s/2013 | Spain | 2 | 1 | 3 | 6 |
| Johnston/2008 | UK | 2 | 2 | 3 | 7 |
| Gorham/2010 | Ireland | 2 | 0 | 3 | 5 |
| Decourt/2013 | USA | 3 | 2 | 3 | 8 |
| Marksteiner/2013 | Austria | 2 | 2 | 3 | 7 |
| Bram/2019 | Brazil | 3 | 2 | 3 | 8 |
| Zimmermann/2005 | Italy | 2 | 2 | 3 | 7 |
| Manzine/2013 | Brazil | 2 | 2 | 3 | 7 |
| Manzine/2013 | Brazil | 3 | 2 | 3 | 7 |
| Le/1993 | France | 3 | 2 | 3 | 8 |
| Davies/1993 | USA | 2 | 0 | 3 | 5 |
| Davies/1997 | USA | 2 | 0 | 3 | 5 |
| FERNANDES/1999 | Portugal | 2 | 0 | 3 | 5 |
| Rˇ ı´pova´-2000 | Czech Republic | 3 | 2 | 2 | 7 |
| Rˇ ı´pova´-2004 | Czech Republic | 3 | 2 | 3 | 8 |
| Vignini/2007 | Italy | 2 | 0 | 3 | 5 |
| Vignini/2013 | Italy | 2 | 2 | 3 | 7 |
| Gattaz/1996 | Germany | 2 | 0 | 3 | 5 |
| Gattaz/2004 | Brazil | 2 | 1 | 3 | 6 |
| Krzystanek/2007 | Poland | 3 | 2 | 3 | 8 |
| Gattaz/2013 | Brazil | 3 | 2 | 3 | 8 |
| Yu/2009 | China | 3 | 2 | 3 | 8 |
| Cohen/1987 | USA | 3 | 2 | 3 | 8 |
| Zubenko/1987 | USA | 3 | 2 | 3 | 8 |
| Zubenko/1987 | USA | 3 | 2 | 3 | 8 |
| Zubenko/1987 | USA | 3 | 2 | 3 | 8 |
| Zubenko/1987 | USA | 3 | 2 | 3 | 8 |
| Zubenko/1987 | USA | 3 | 2 | 3 | 8 |
| Zubenko/1988 | USA | 3 | 2 | 3 | 8 |
| Hajimohammadreza/1990 | UK | 2 | 2 | 3 | 7 |
| Adunsky/1989 | Israel | 3 | 1 | 3 | 7 |
| Merighi/2021 | Italy | 2 | 2 | 3 | 7 |
| Mukaetova-Ladinska/2012 | UK | 3 | 1 | 3 | 7 |
| Adolfsson/1980 | Sweden | 2 | 2 | 3 | 7 |
| Smith/1982 | USA | 2 | 2 | 4 | 8 |
| Danielczyk/1988 | Austria | 2 | 0 | 3 | 5 |
| Schneider/1988 | USA | 2 | 2 | 3 | 7 |
| Bonuccelli/1990 | Italy | 2 | 1 | 3 | 6 |
| Regland/1991 | Sweden | 2 | 0 | 3 | 5 |
| Parnetti/1992 | Italy | 3 | 1 | 3 | 7 |
| Ahlskog/1996 | USA | 3 | 2 | 3 | 8 |
| Fitzgerald/1996 | Ireland | 2 | 0 | 3 | 5 |
| Bongioanni/1996 | Italy | 3 | 1 | 3 | 7 |
| Bongioanni/1997 | Italy | 3 | 2 | 3 | 8 |
| Soto/1999 | Spain | 3 | 2 | 3 | 8 |
| PRODAN/2006 | USA | 2 | 1 | 3 | 6 |
| Prodan/2009 | USA | 3 | 2 | 3 | 8 |
| Bongioanni/1997 | Italy | 3 | 1 | 3 | 7 |
| Nemeroff/1988 | England | 2 | 2 | 3 | 7 |
| Galzin/1989 | France | 3 | 1 | 3 | 7 |
| HERSHKOWITZ/1996 | Israel | 3 | 2 | 3 | 8 |
| Bacchetti/2015 | Italy | 3 | 2 | 3 | 8 |
| Mukaetova-Ladinska/2012 | UK | 2 | 1 | 3 | 6 |
| BOSMAN/1992 | Netherlands | 2 | 0 | 3 | 5 |
| Lanius/1997 | Canada | 3 | 0 | 3 | 6 |
| MATSUSHIMA/1995 | Japan | 2 | 1 | 3 | 6 |
| Wang/2019 | China | 3 | 2 | 3 | 8 |
| Odaka/2021 | Japan | 2 | 0 | 3 | 5 |
| Forlenza/2011 | Brazil | 2 | 1 | 3 | 6 |
| Pláteník/2014 | Czech Republic | 2 | 2 | 3 | 7 |
