# Supplementary material for: Meta-analysis and systematic review of peripheral platelet-associated biomarkers to explore the pathophysiology of alzheimer's disease
Source: BMC Neurol. 2023 Feb 11;23:66. doi: 10.1186/s12883-023-03099-5 (PMC9921402; doi:10.1186/s12883-023-03099-5)
Supplement: Supplementary file 6 — Additional file 6: Figure S1. Forest plot for APP(130kDa106-110kDa. Figure S2. Forest plot for ADAM10. Figure S3. Forest plot for ADAM10/actin. Figure S4. Forest plot for BCEA. Figure S5. Forest plot for PSEN. Figure S6. HMWtau/LMWtau. Figure S7. Forest plot for NO production. Figure S8. Forest plot for ONOO- production. Figure S9. Forest plot for Ca2+. Figure S10. Forest plot for Na+-K+-ATPase. Figure S11. Forest plot for MAO-B. Figure S12. Forest plot for 5-HT. Figure S13. Forest plot for 5-HT(Vmax). Figure S14. Forest plot for 5-HT(Km). Figure S15. Forest plot for A2 Receptor. Figure S16. Forest plot for PLA2. Figure S17. Forest plot for DPH(Fluorescence Lifetime). Figure S18. Forest plot for DPH(Steady-State Anisotropy). Figure S19. Forest plot for TMA-DPH(Fluorescence Lifetime). Figure S20. Forest plot for TMA-DPH(Steady-State Anisotropy). [file 12883_2023_3099_MOESM6_ESM.pdf]

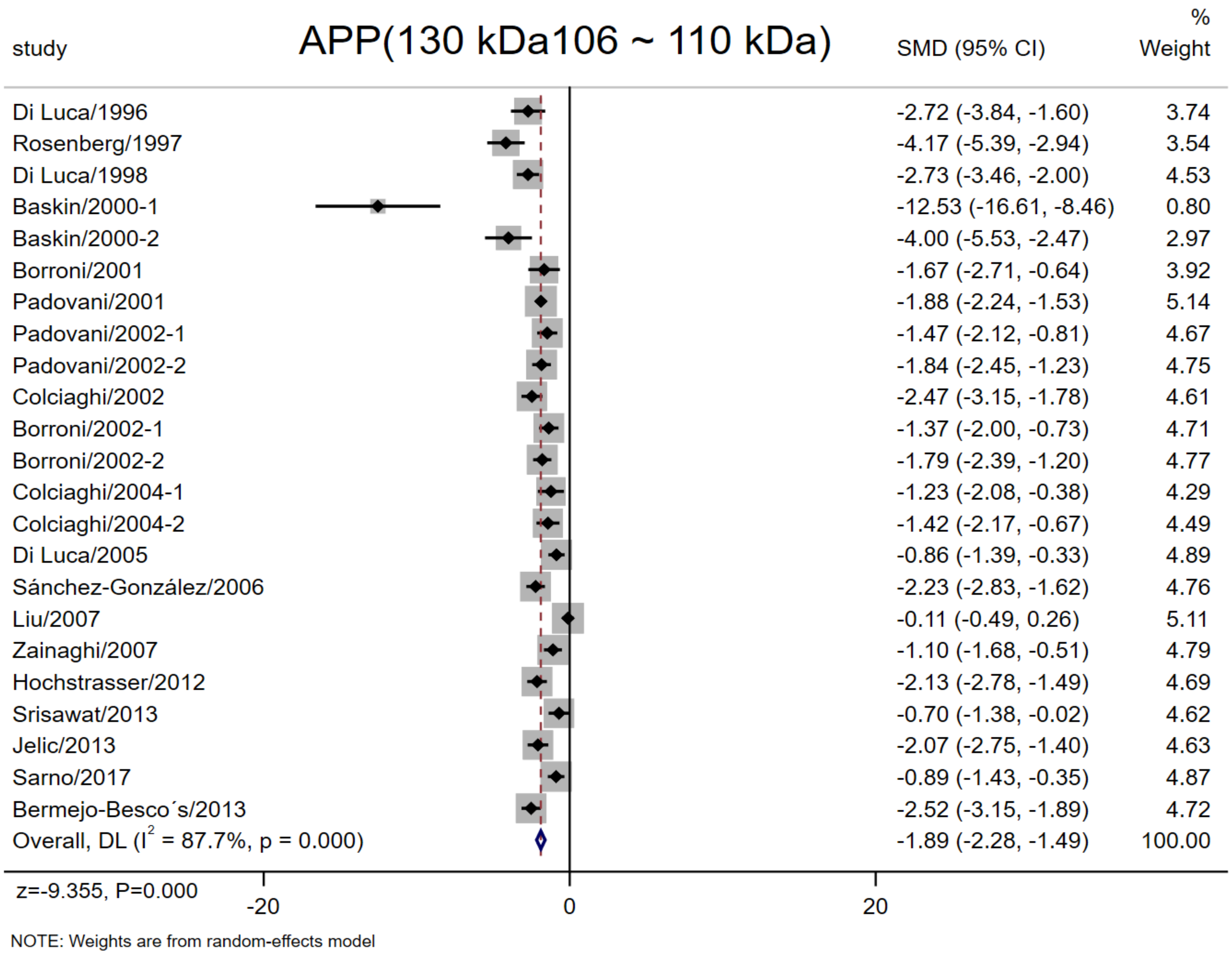

Figure S1: Forest plot for APP(130kDa106-110kDa)

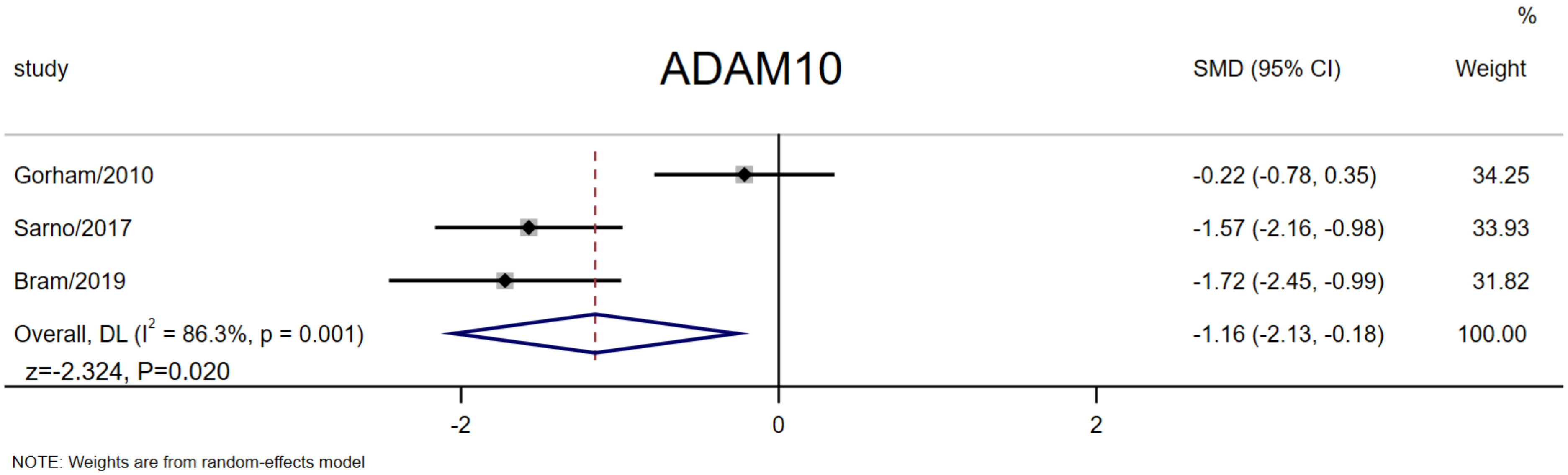

Figure S2: Forest plot for ADAM10

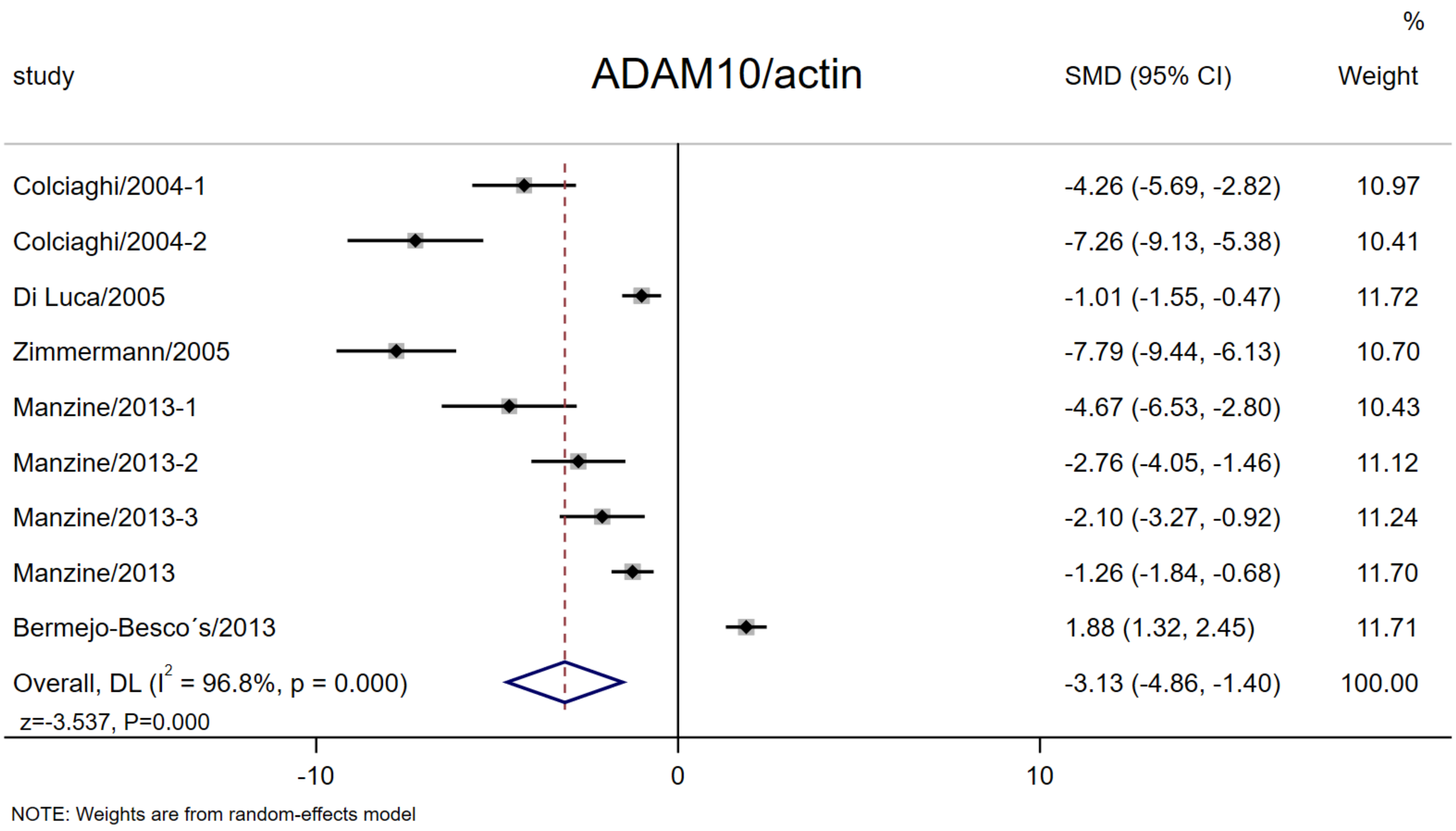

Figure S3: Forest plot for ADAM10/actin

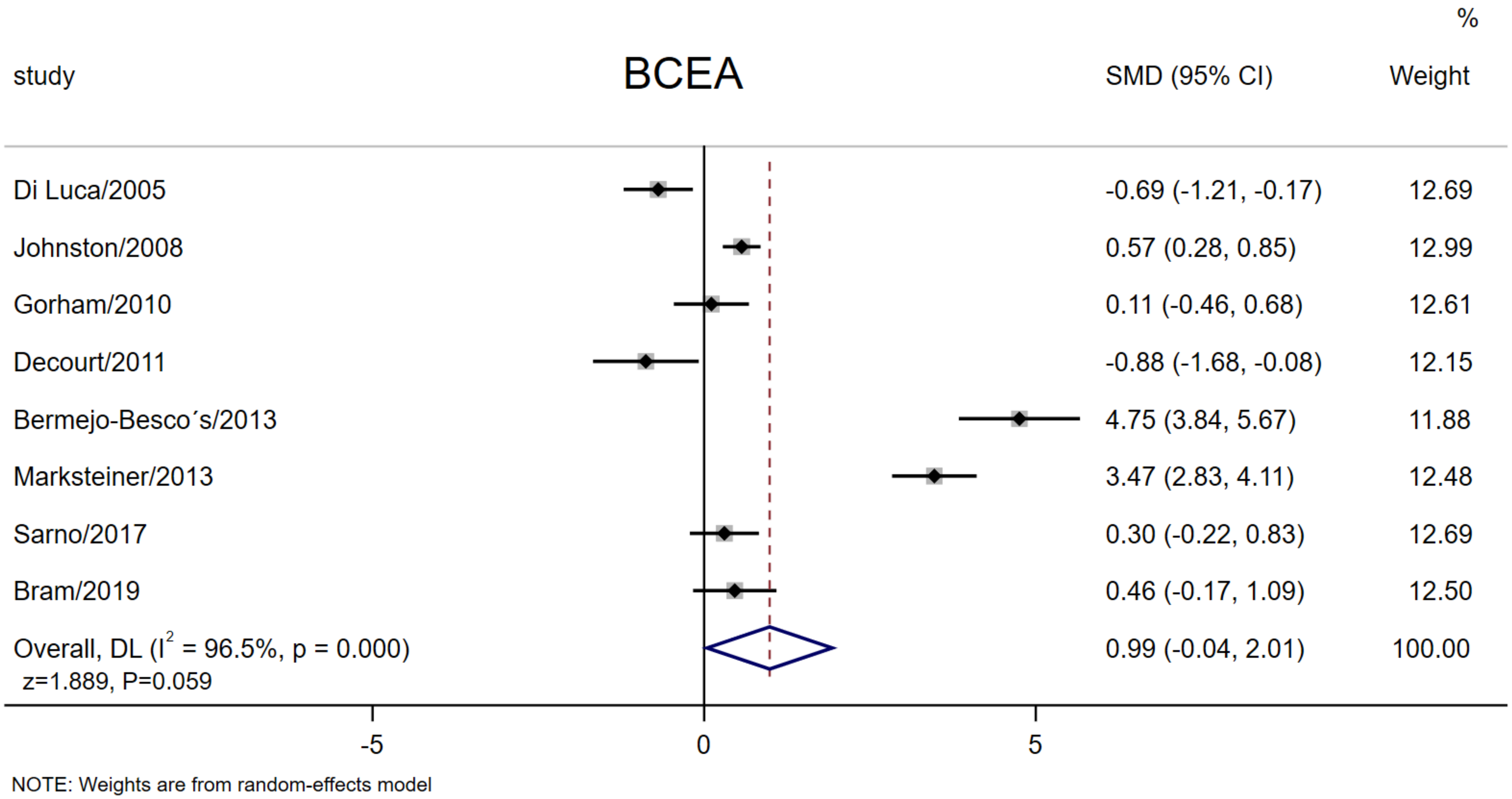

Figure S4: Forest plot for BCEA

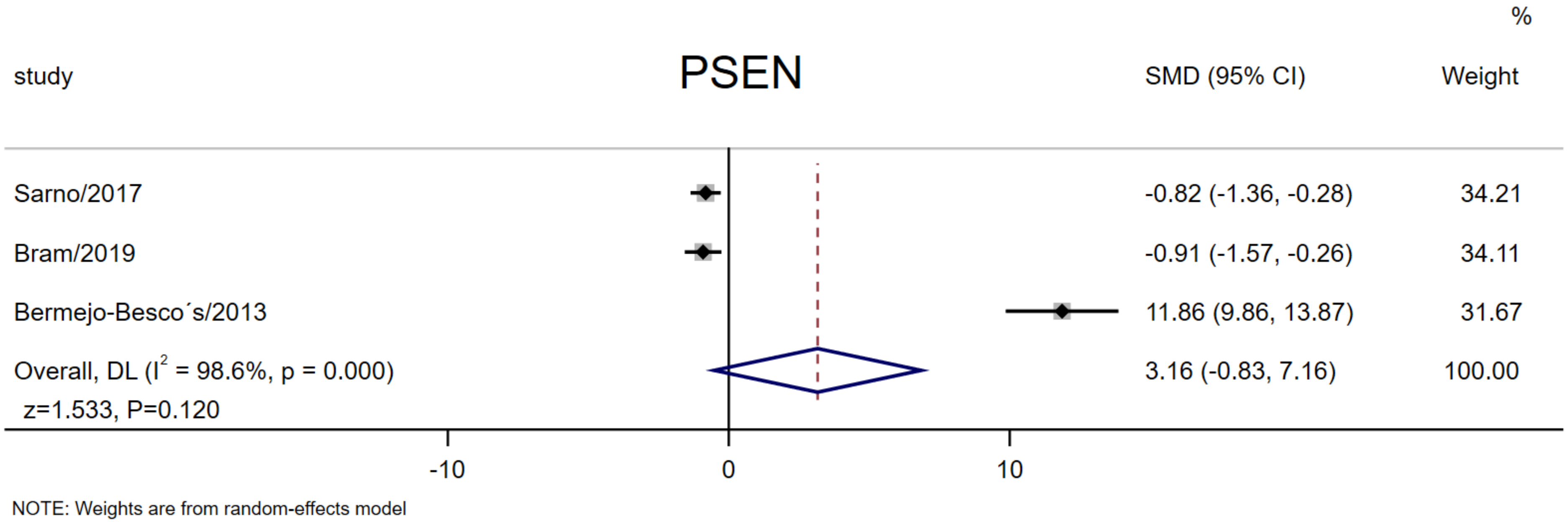

Figure S5: Forest plot for PSEN

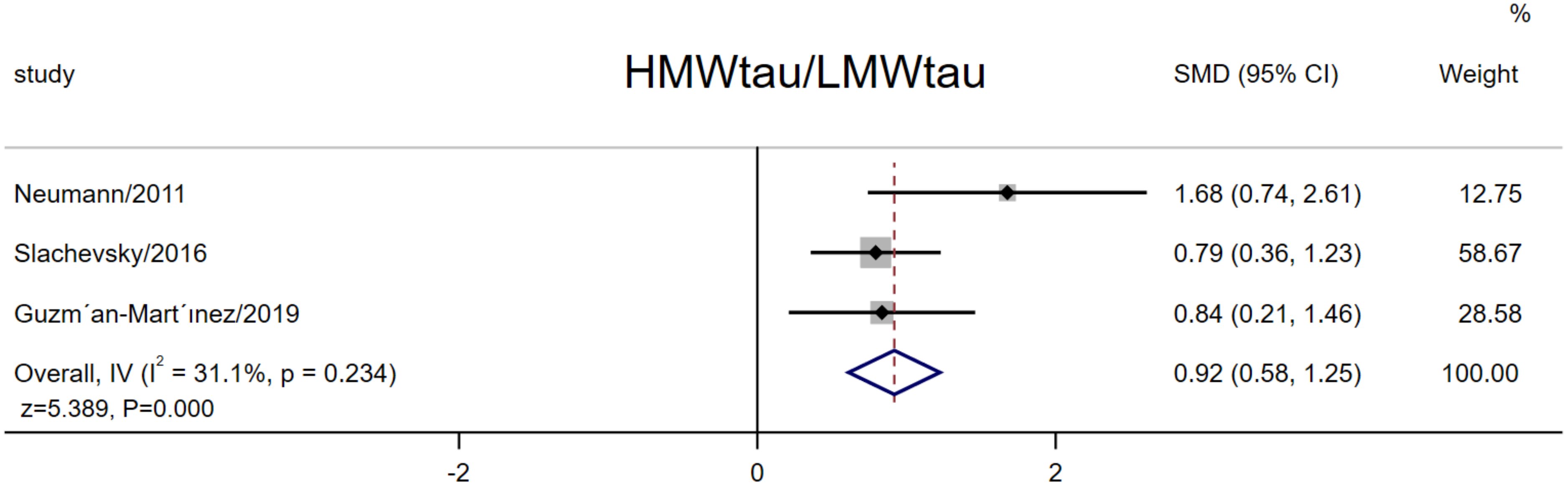

Figure S6: HMWtau/LMWtau

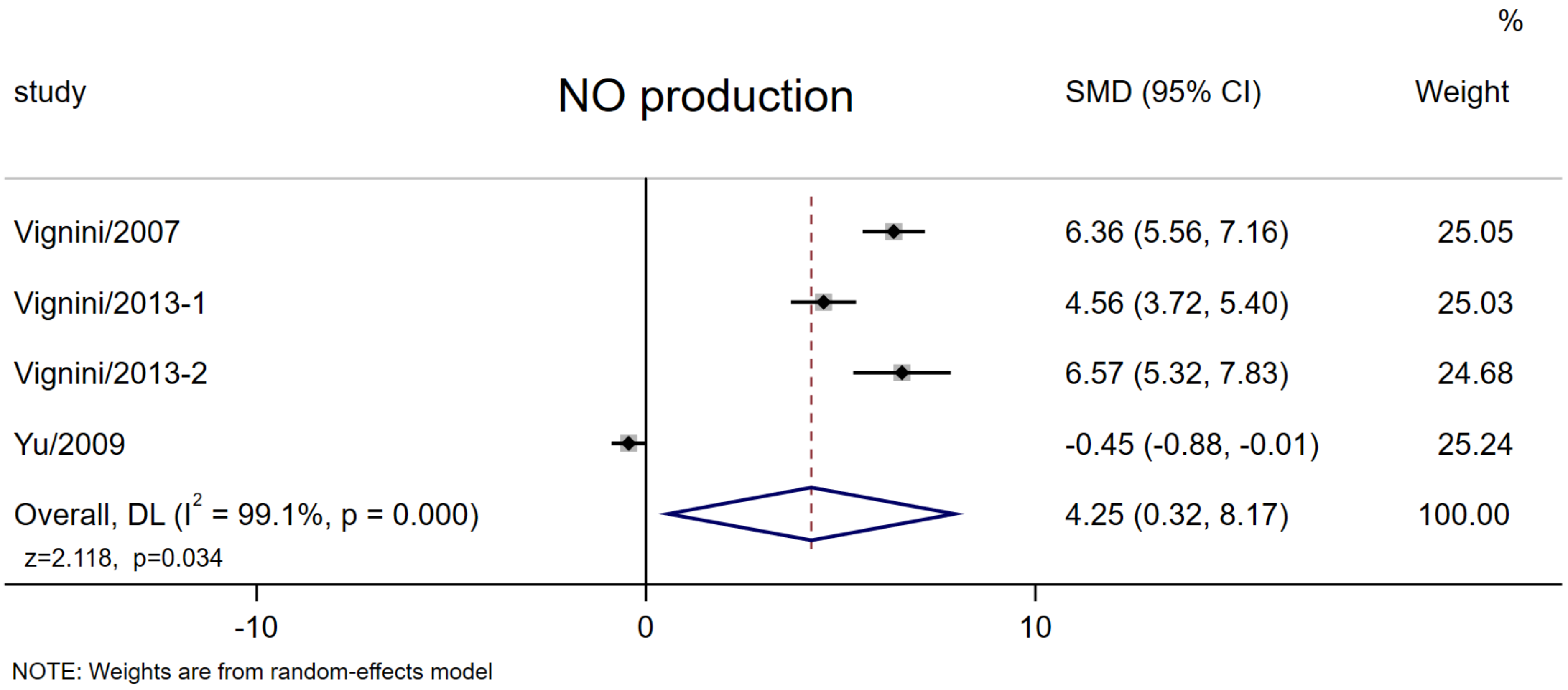

Figure S7: Forest plot for NO production

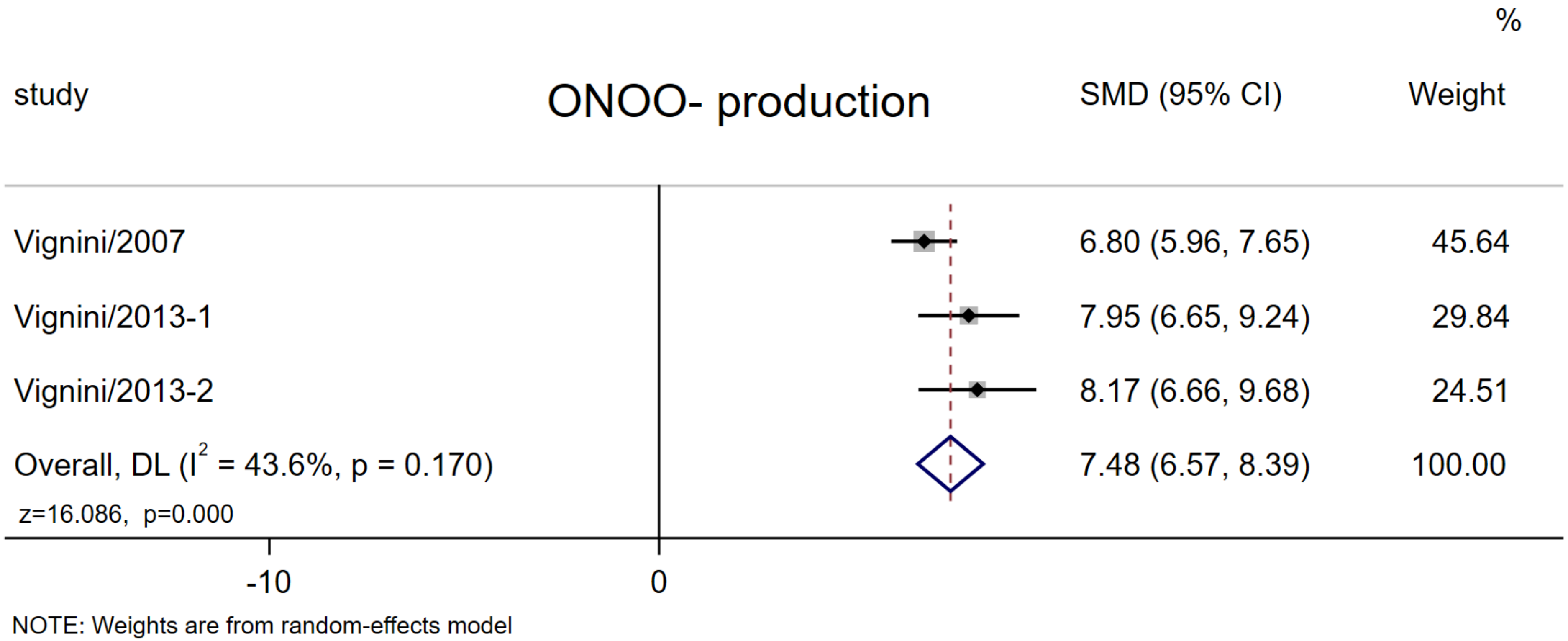

Figure S8: Forest plot for ONOO- production

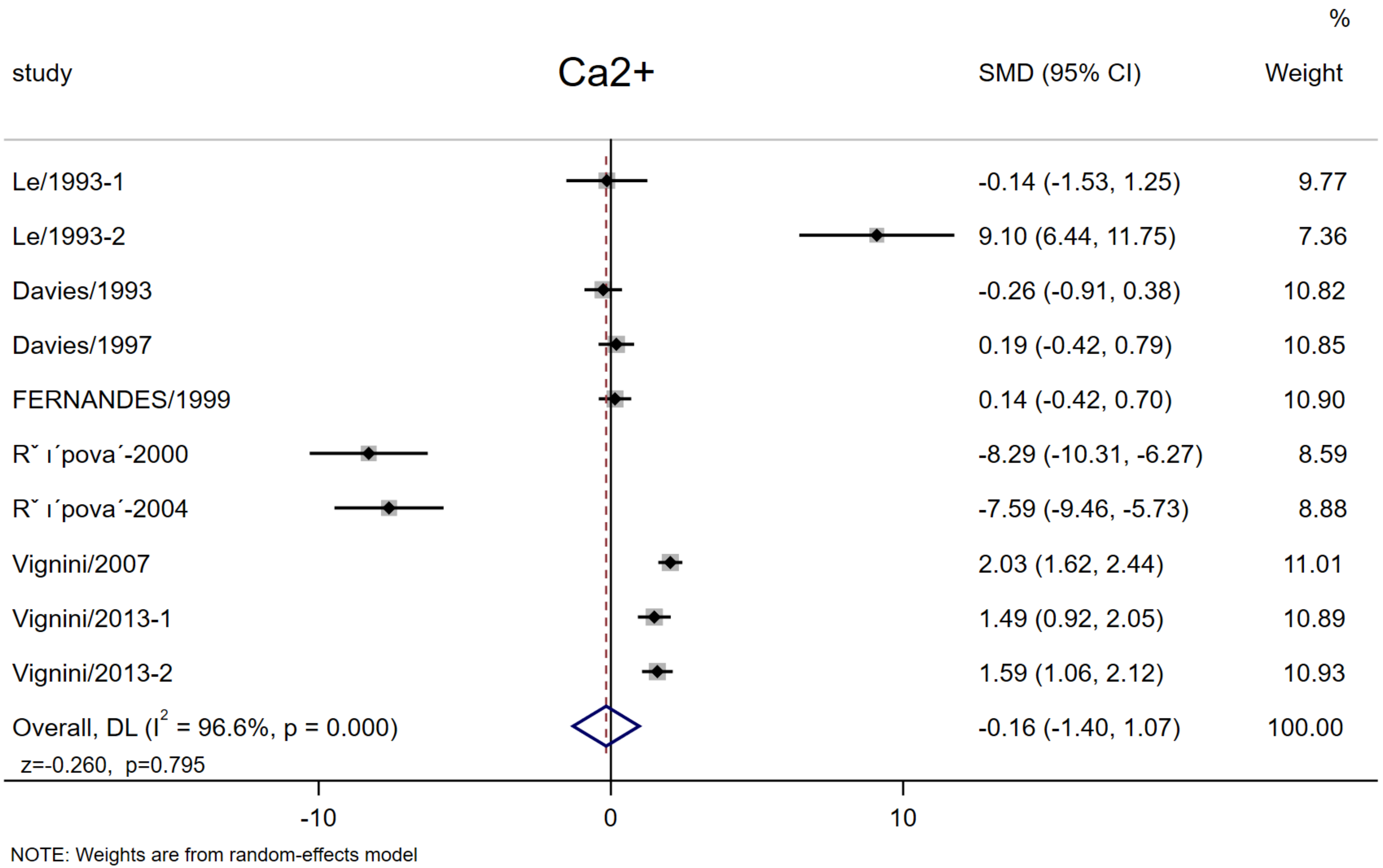

Figure S9: Forest plot for Ca2+

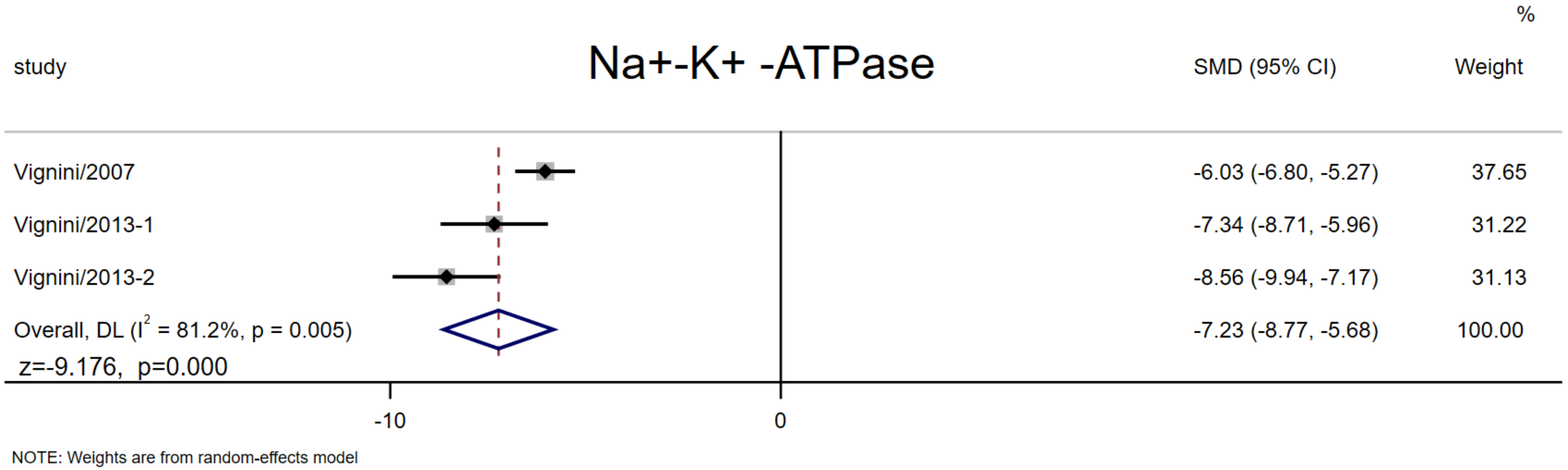

Figure S10: Forest plot for Na+-K+-ATPase

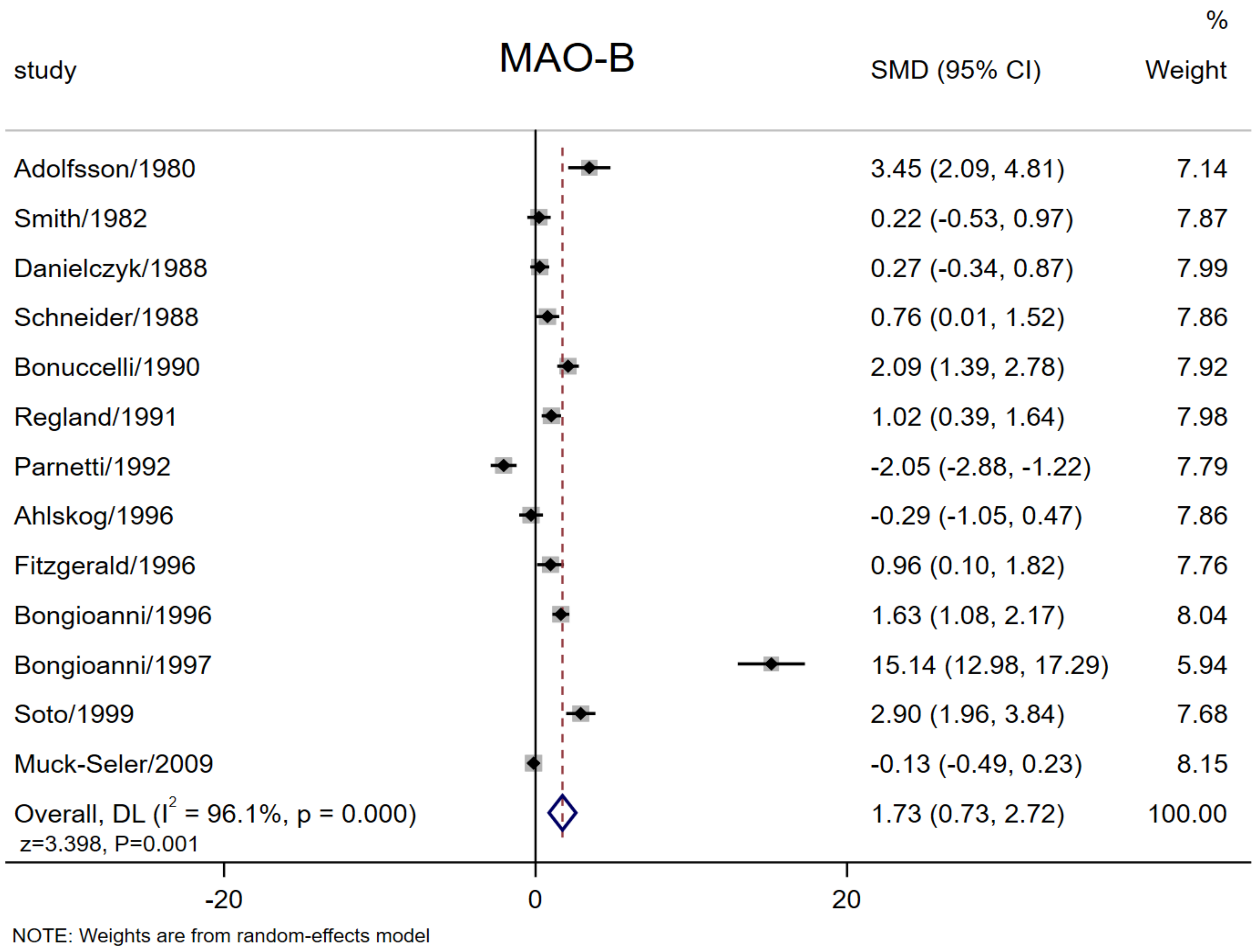

Figure S11: Forest plot for MAO-B

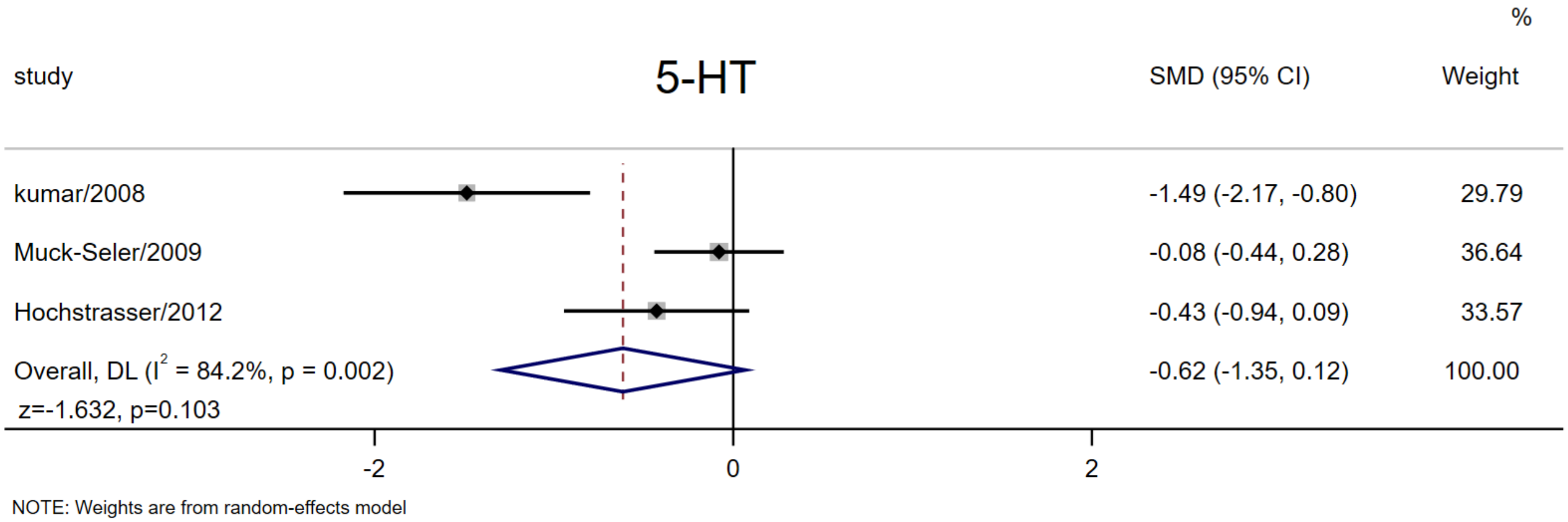

Figure S12: Forest plot for 5-HT

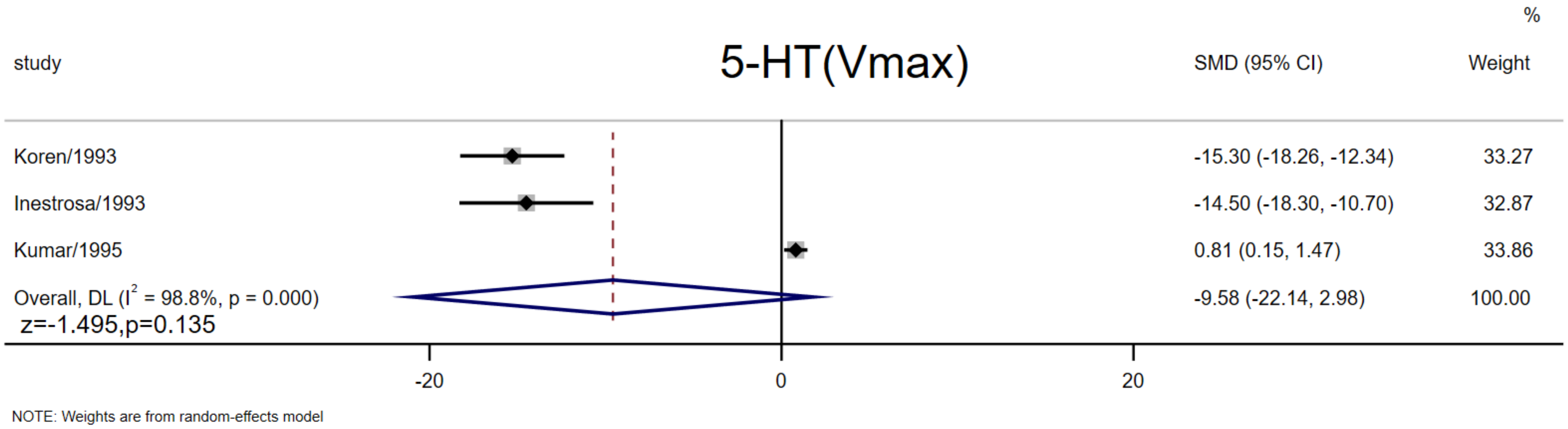

Figure S13: Forest plot for 5-HT(Vmax)

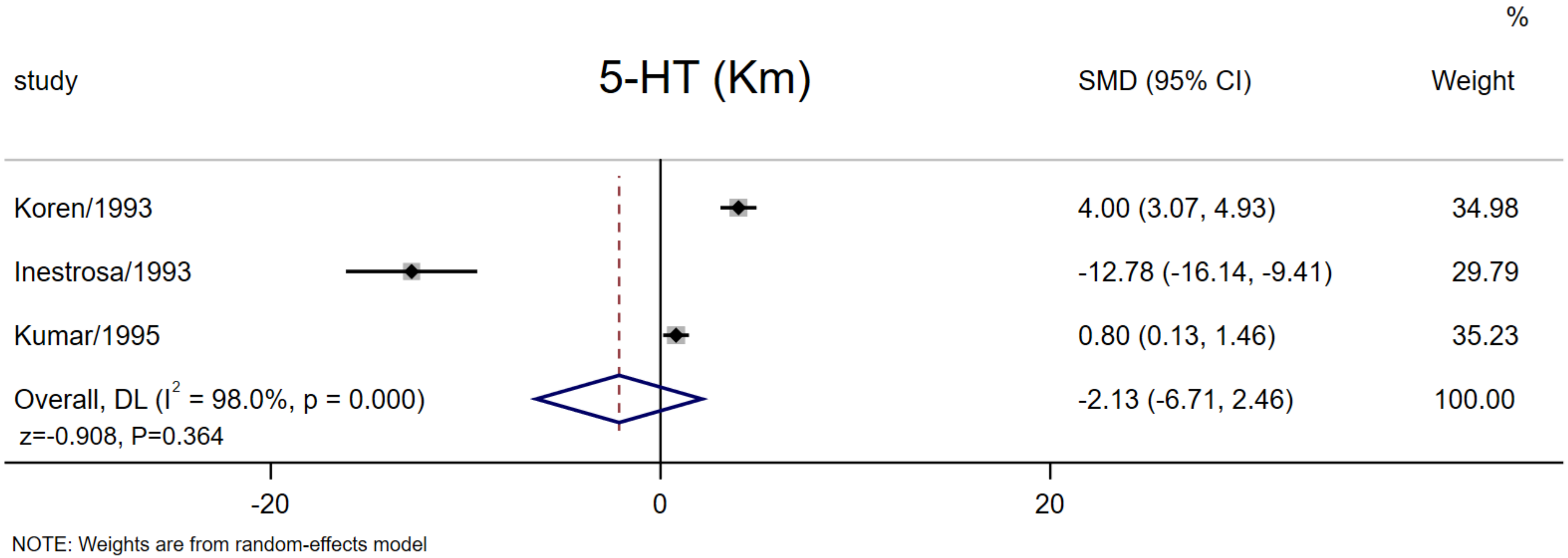

Figure S14: Forest plot for 5-HT(Km)

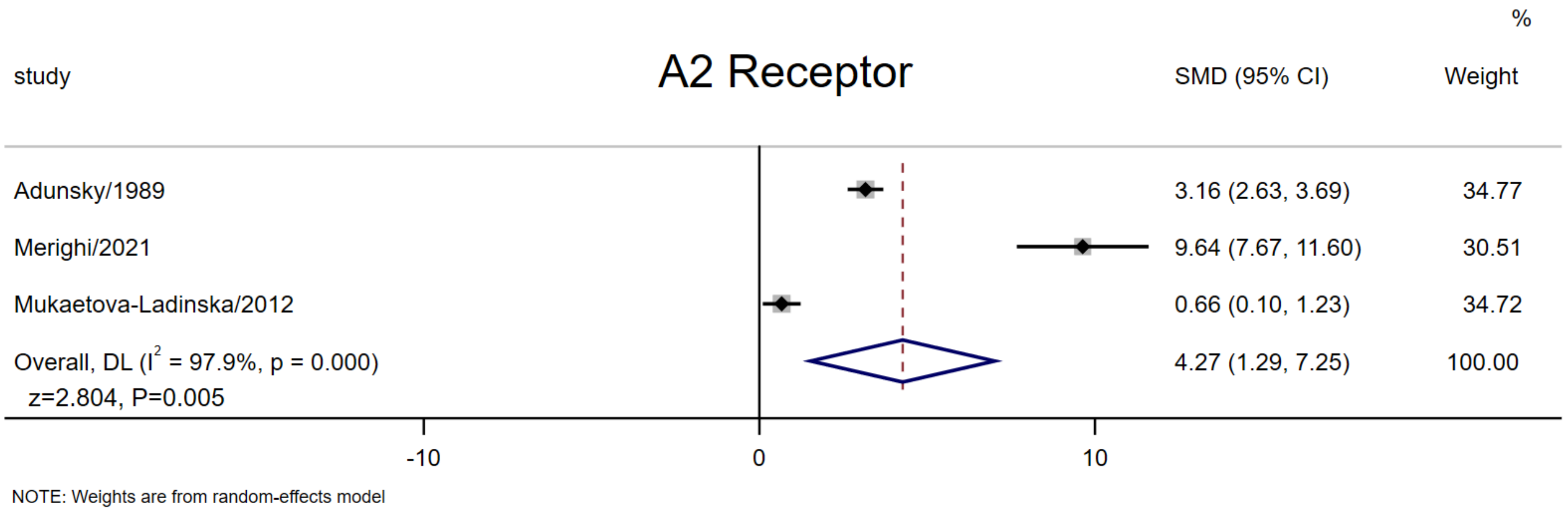

Figure S15: Forest plot for A2 Receptor

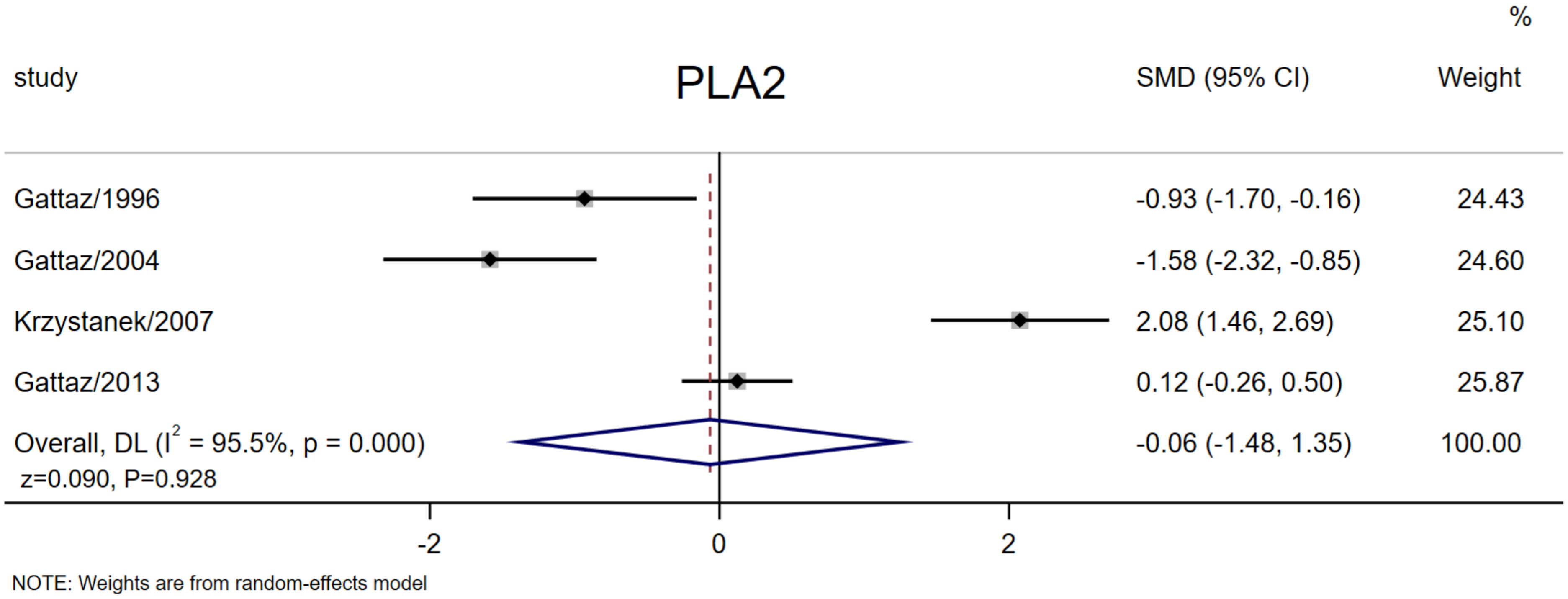

Figure S16: Forest plot for PLA2

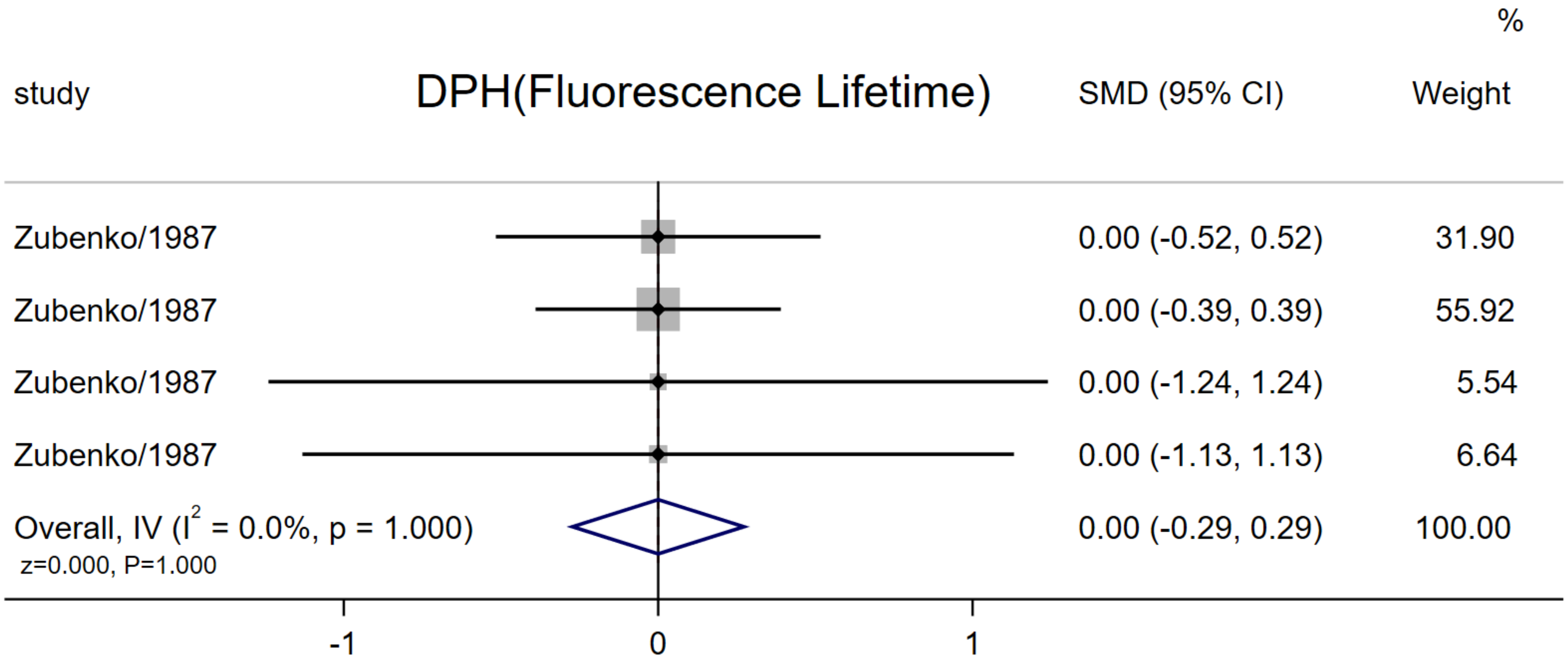

Figure S17: Forest plot for DPH(Fluorescence Lifetime)

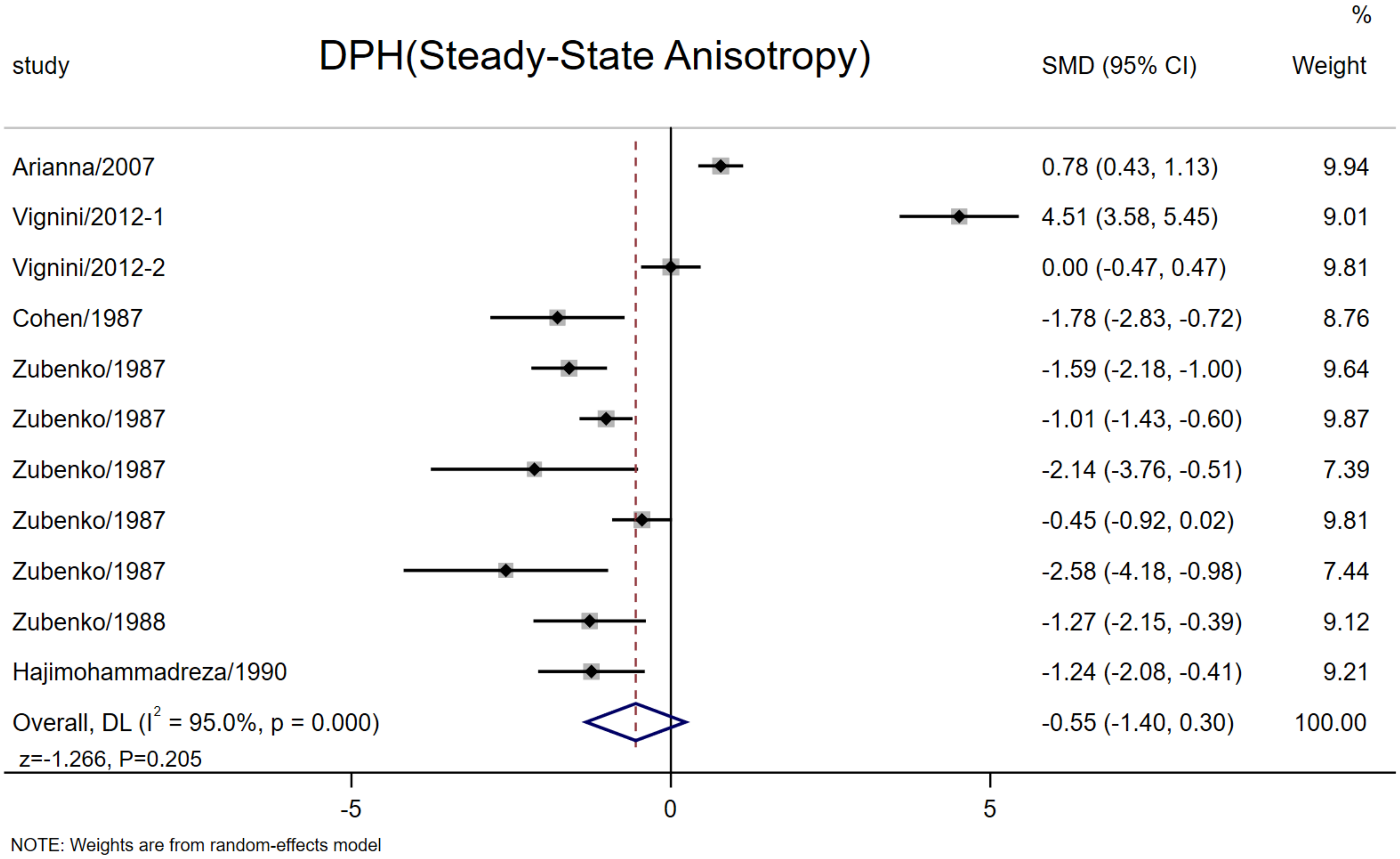

Figure S18: Forest plot for DPH(Steady-State Anisotropy)

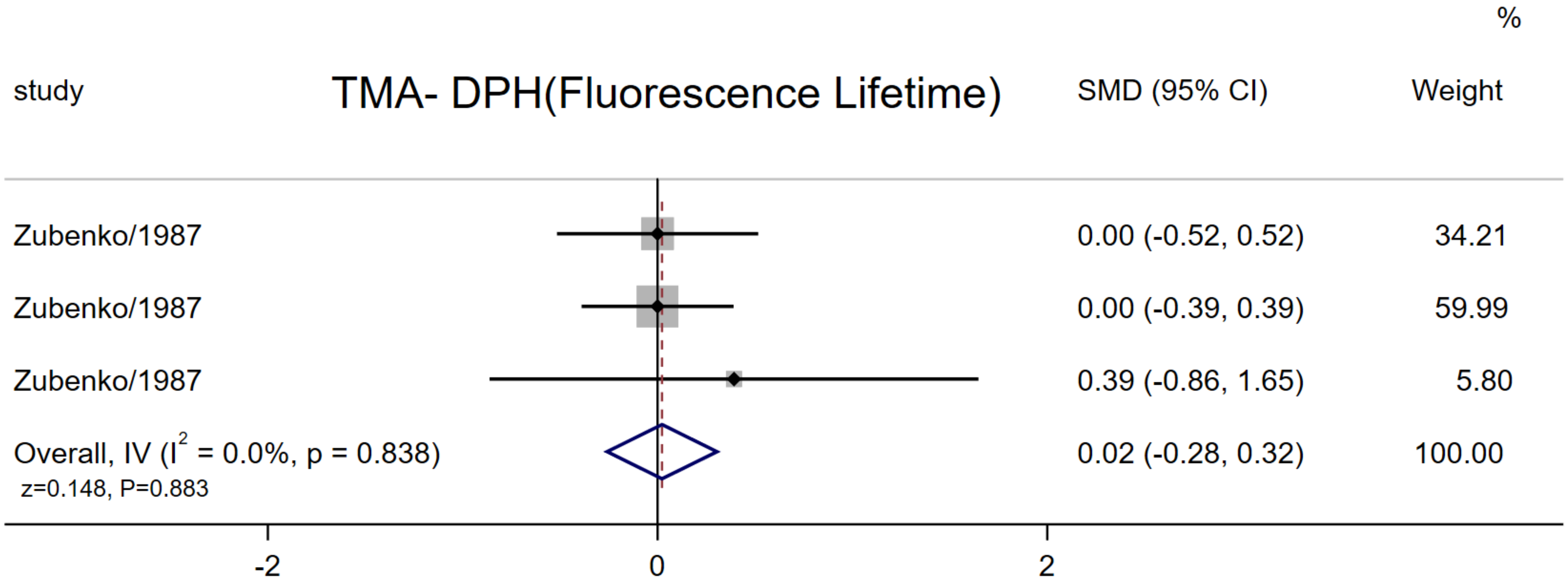

Figure S19: Forest plot for TMA-DPH(Fluorescence Lifetime)

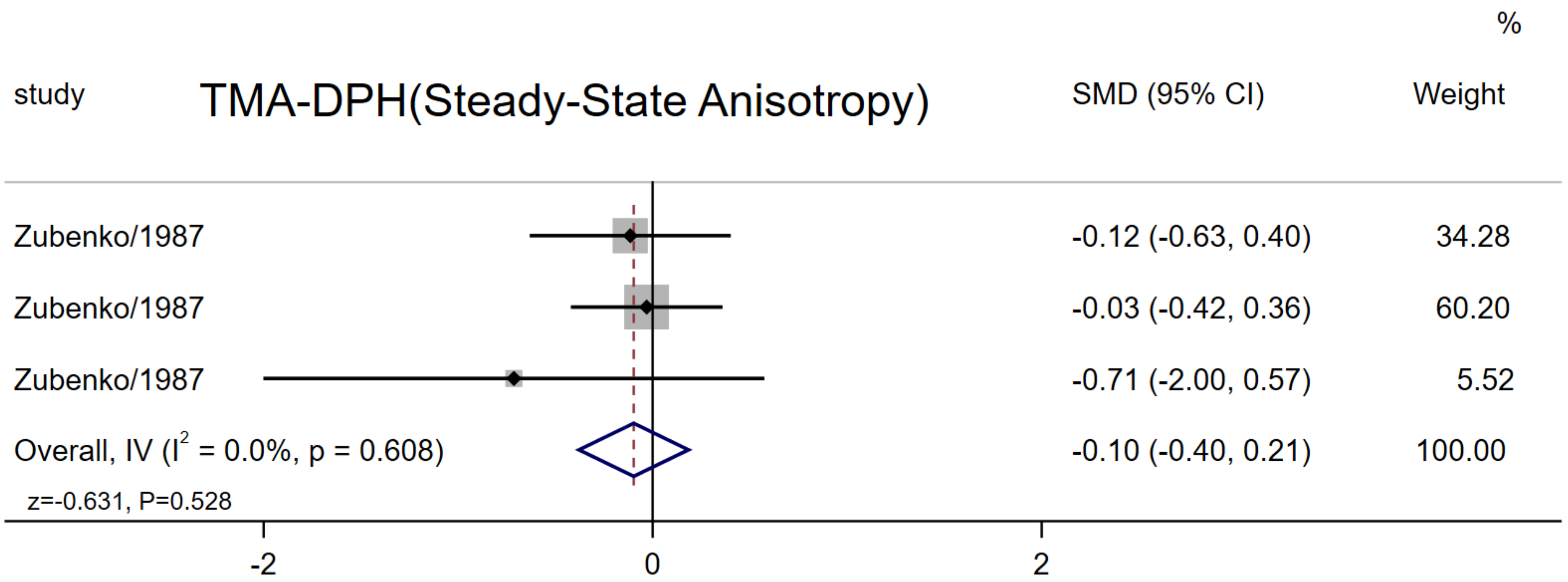

Figure S20: Forest plot for TMA-DPH(Steady-State Anisotropy)
