# Supplementary material for: Meta-analysis and systematic review of peripheral platelet-associated biomarkers to explore the pathophysiology of alzheimer's disease
Source: BMC Neurol. 2023 Feb 11;23:66. doi: 10.1186/s12883-023-03099-5 (PMC9921402; doi:10.1186/s12883-023-03099-5)
Supplement: Supplementary file 7 — Additional file 7: Figure S21. Sensitivity analysis for APP(130 kDa/106-110kDa). Figure S22. funnel plot of APP(130 kDa/106-110kDa); Egger’s test: p=0.001. Figure S23. filled funnel plot of APP(130 kDa/106-110kDa). Figure S24. Sensitivity analysis for ADAM-10. Figure S25. funnel plot of ADAM-10; Egger’s test: p＞0.05. Figure S26. Sensitivity analysis for ADAM-10/actin. Figure S27. funnel plot of ADAM-10/actin; Egger’s test: p=0.010. Figure S28. filled funnel plot of ADAM-10/actin. Figure S29. Sensitivity analysis for BACE-1. Figure S30. funnel plot of BCEA-1; Egger’s test: p＞0.05. Figure S31. Sensitivity analysis for PSEN-1. Figure S32. funnel plot of PSEN-1; Egger’s test: p＞0.05. Figure S33. Sensitivity analysis for HMWtau/LMWtau. Figure S34. funnel plot of HMWtau/LMWtau; Egger’s test: p＞0.05. Figure S35. Sensitivity analysis for NO production. Figure S36. funnel plot of NO production; Egger’s test: p＞0.05. Figure S37. Sensitivity analysis for ONOO- production. Figure S38. funnel plot of ONOO- production; Egger’s test: p＞0.05. Figure S39. Sensitivity analysis for Ca2+. Figure S40. funnel plot of Ca2+; Egger’s test: p＞0.05. Figure S41. Sensitivity analysis for Na+-K+ -ATPase. Figure S42. funnel plot of Na+ -K+ -ATPase; Egger’s test: p＞0.05. Figure S43. Sensitivity analysis for MAO-B. Figure S44. funnel plot of MAO-B; Egger’s test: p=0.025. Figure S45. filled funnel plot of MAO-B. Figure S46. Sensitivity analysis for 5-HT. Figure S47. funnel plot of 5-HT; Egger’s test: P＞0.05. Figure S48. Sensitivity analysis for 5-HT(Bmax). Figure S49. funnel plot of 5-HT(Bmax); Egger’s test: P＞0.05. Figure S50. Sensitivity analysis for 5-HT(Vmax). Figure S51. funnel plot of 5-HT(Vmax); Egger’s test: P＞0.05. Figure S52. Sensitivity analysis for 5-HT(Km). Figure S53. funnel plot of 5-HT(Km); Egger’s test: P＞0.05. Figure S54. Sensitivity analysis for A2Receptor. Figure S55. funnel plot of A2Receptor; Egger’s test: P＞0.05. Figure S56. Sensitivity analysis for PLA2. Figure S57. funnel pl [file 12883_2023_3099_MOESM7_ESM.pdf]

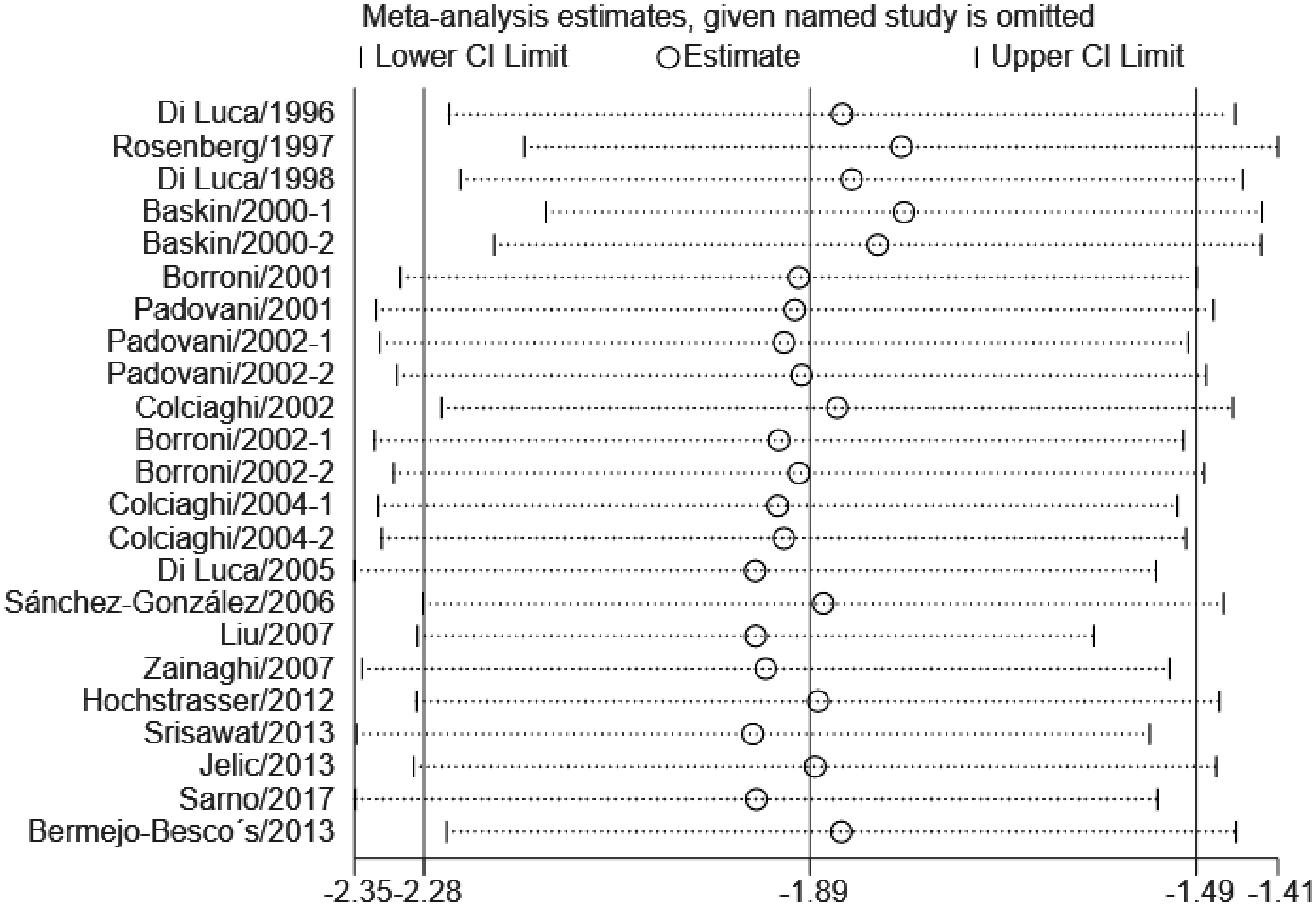

Figure S21: Sensitivity analysis for APP(130 kDa/106-110kDa)

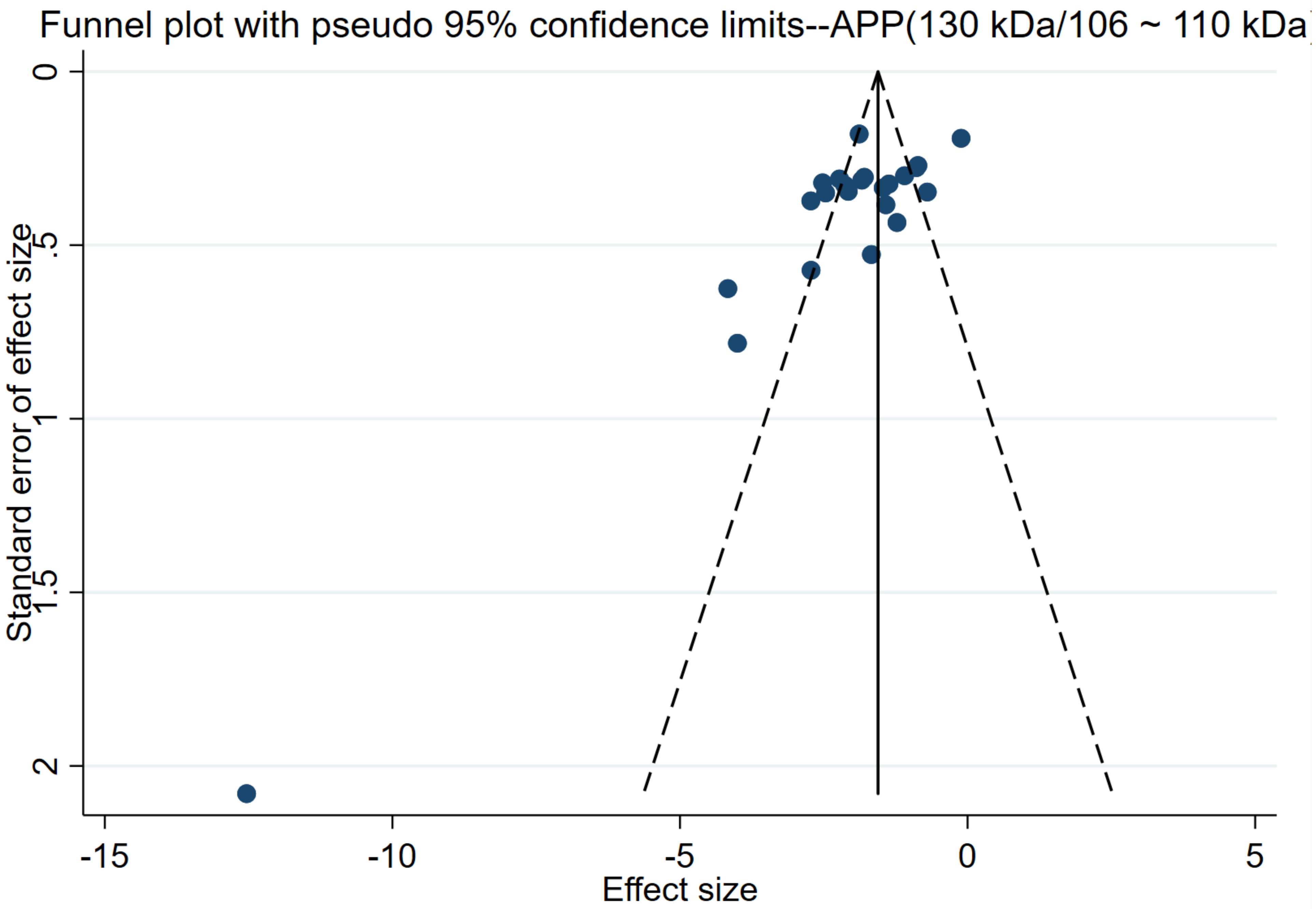

Figure S22: funnel plot of APP(130 kDa/106-110kDa); Egger's test: p=0.001

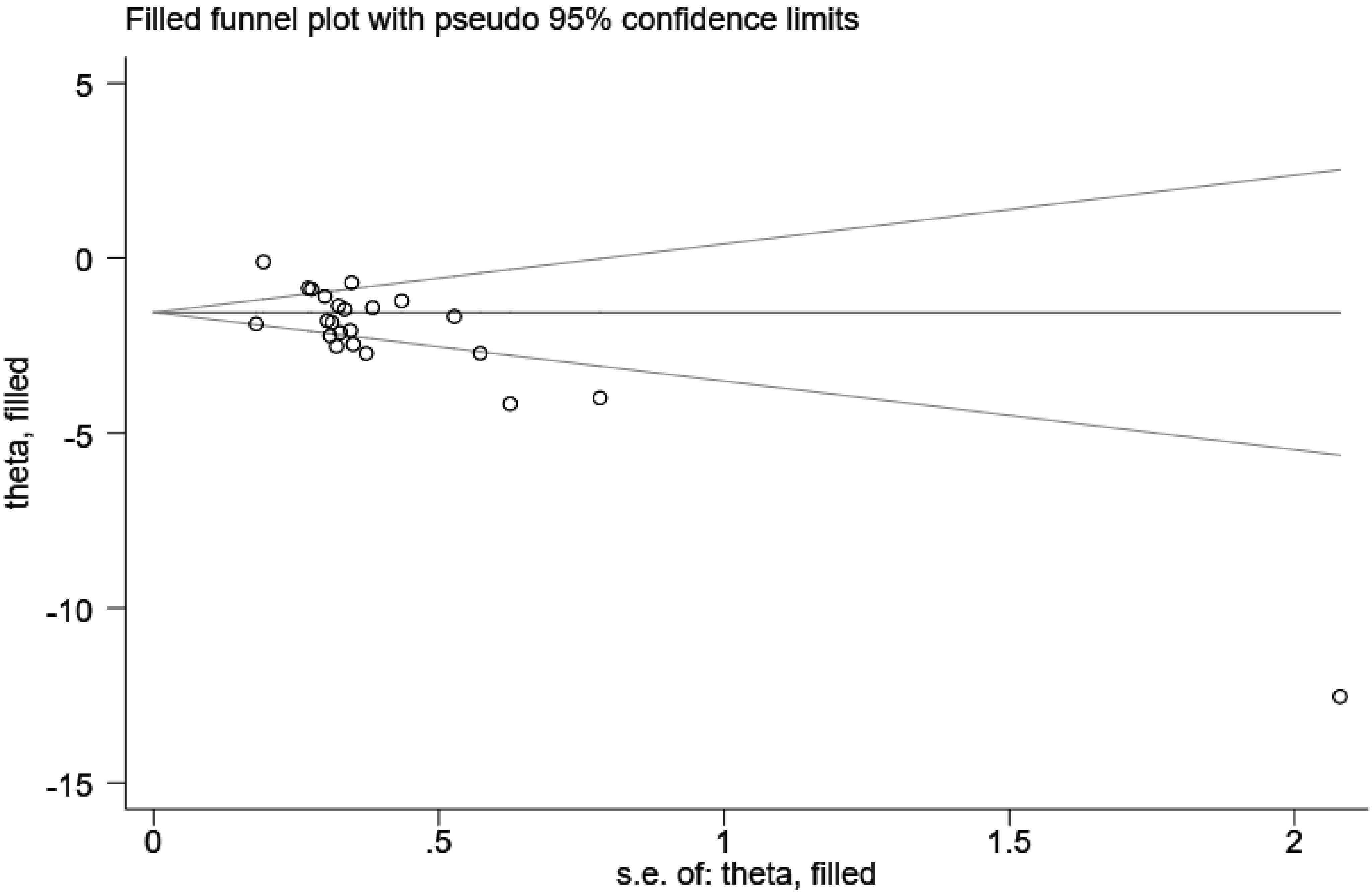

Figure S23: filled funnel plot of APP(130 kDa/106-110kDa)

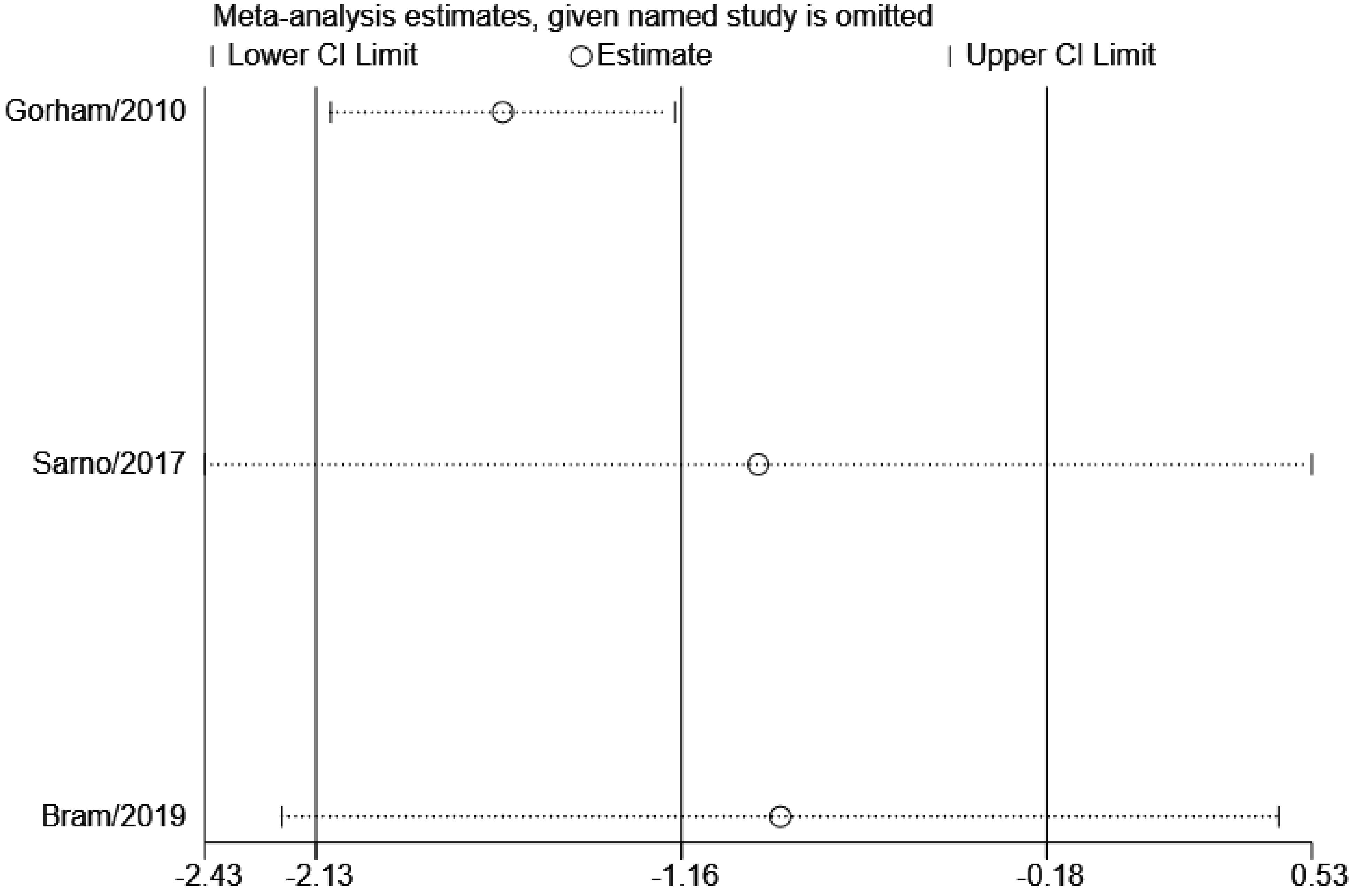

Figure S24: Sensitivity analysis for ADAM-10

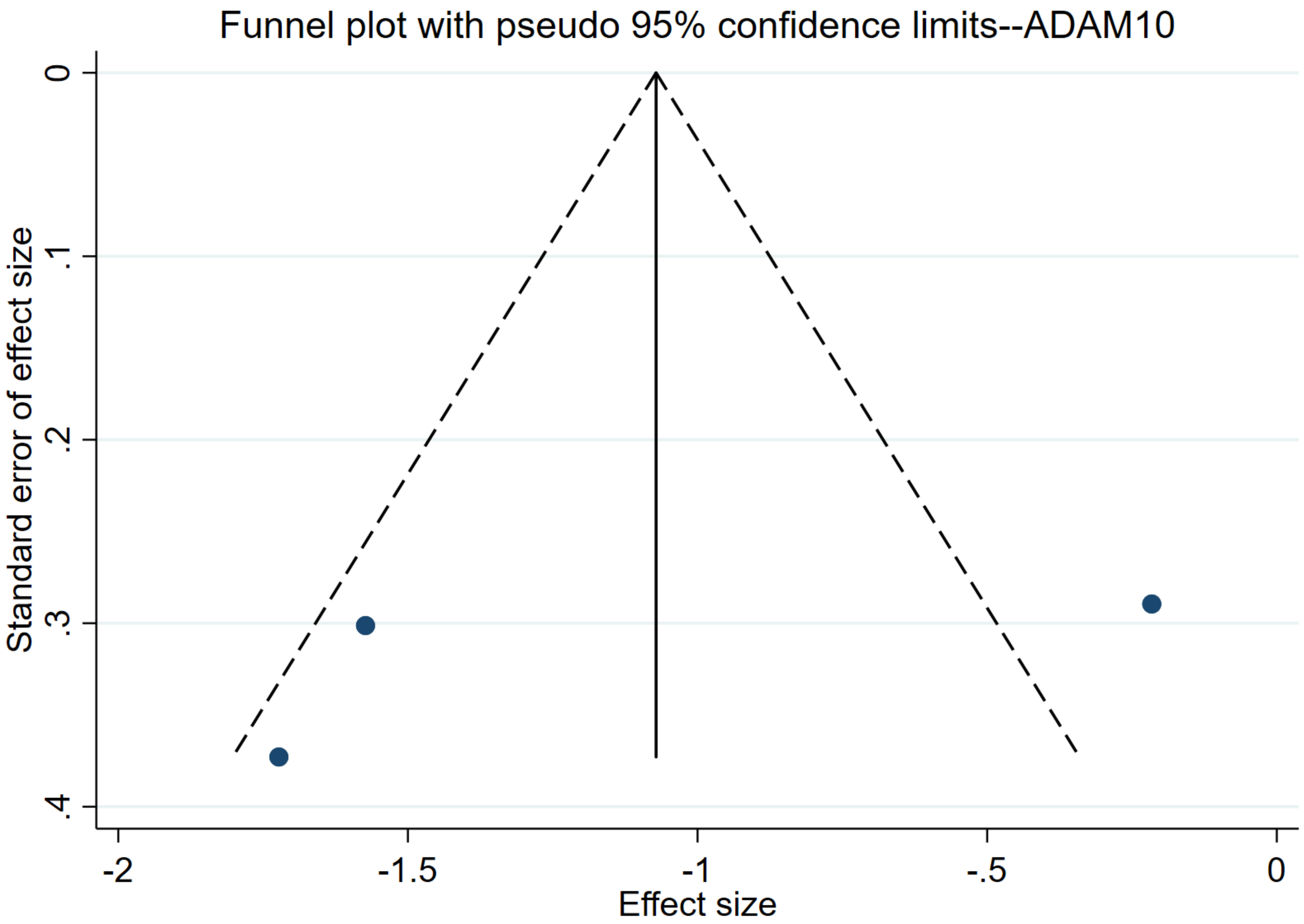

Figure S25: funnel plot of ADAM-10; Egger's test: $p > 0.05$

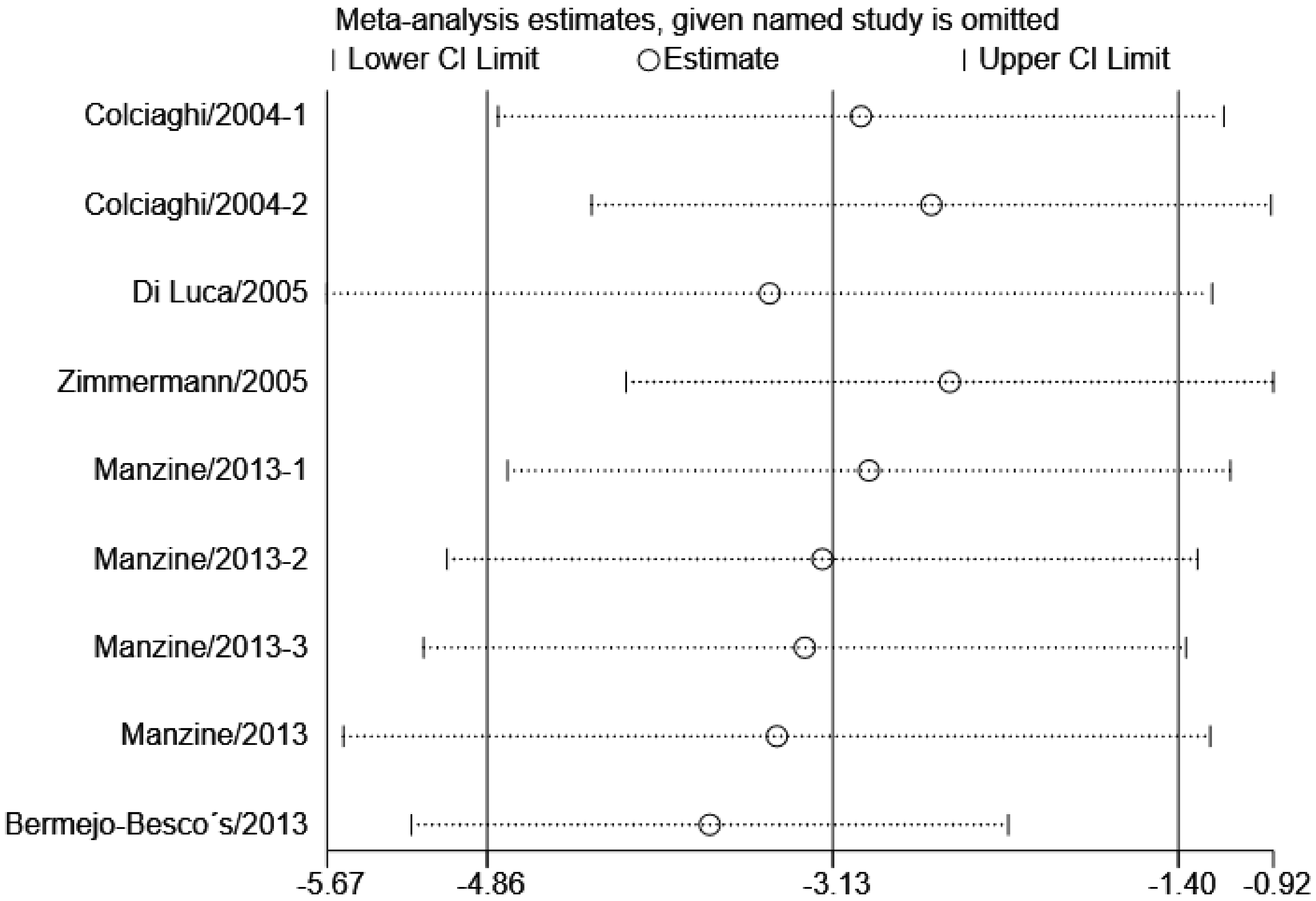

Figure S26: Sensitivity analysis for ADAM-10/actin

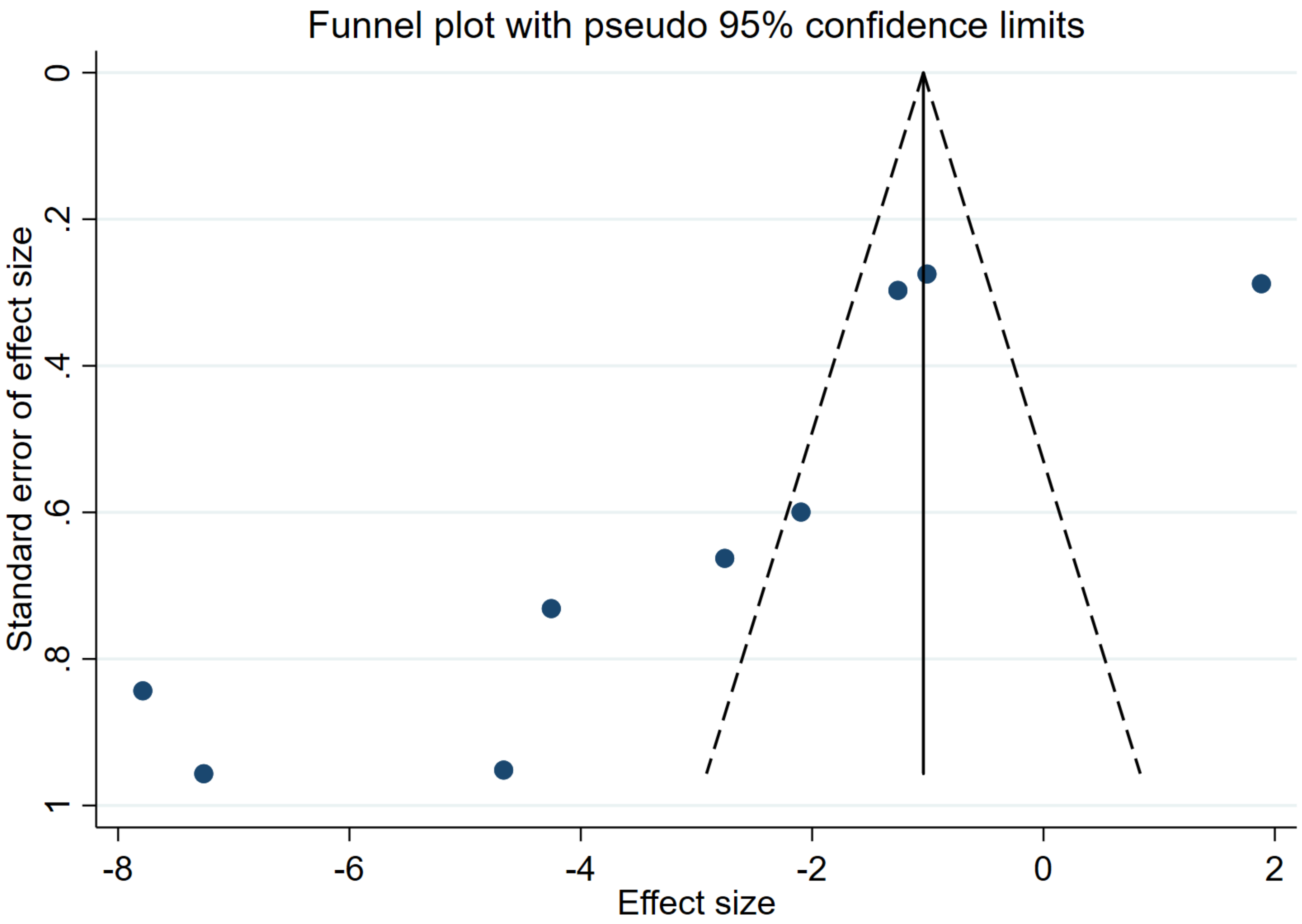

Figure S27: funnel plot of ADAM-10/actin; Egger's test: p=0.010

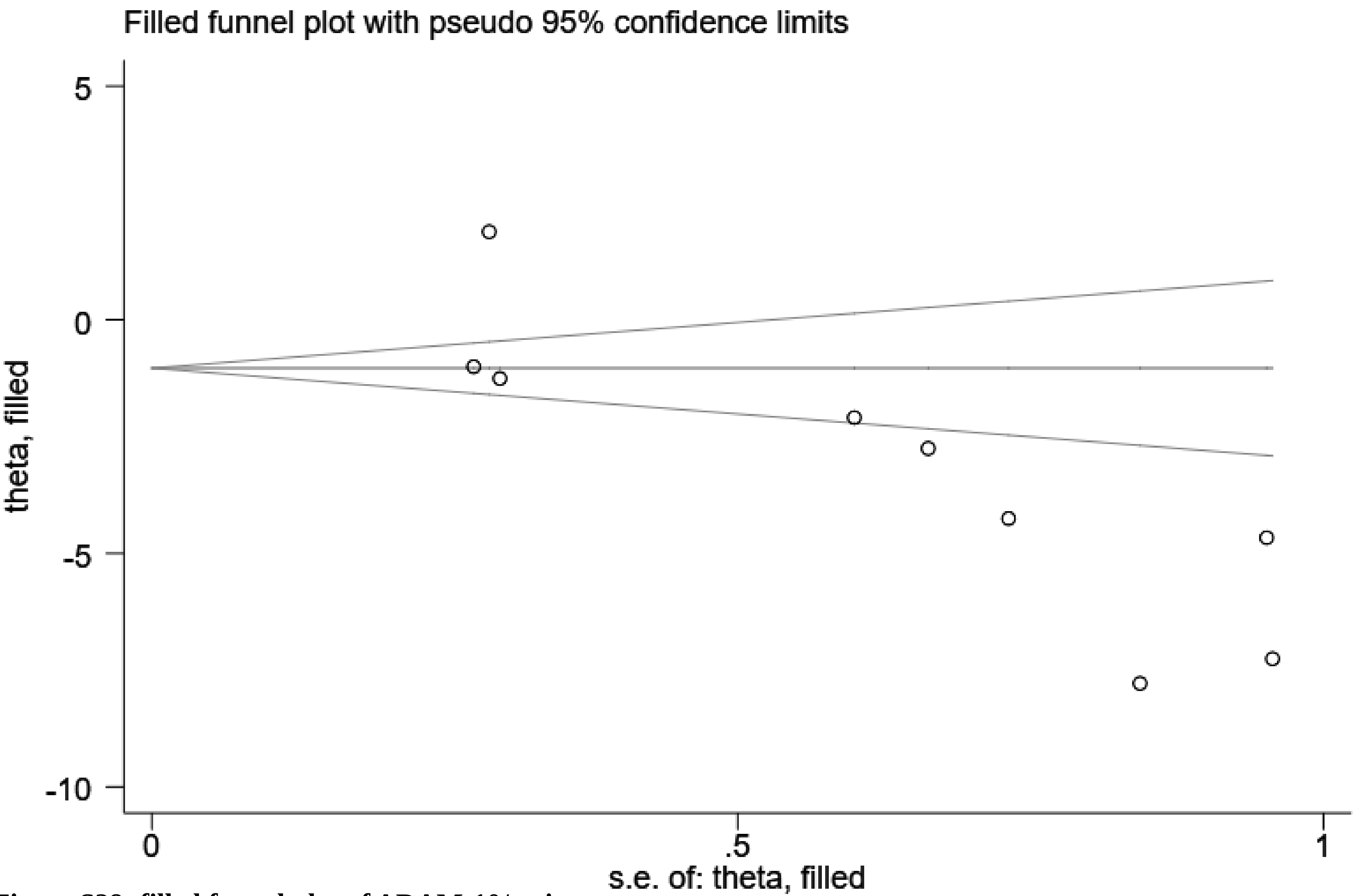

Figure S28: filled funnel plot of ADAM-10/actin

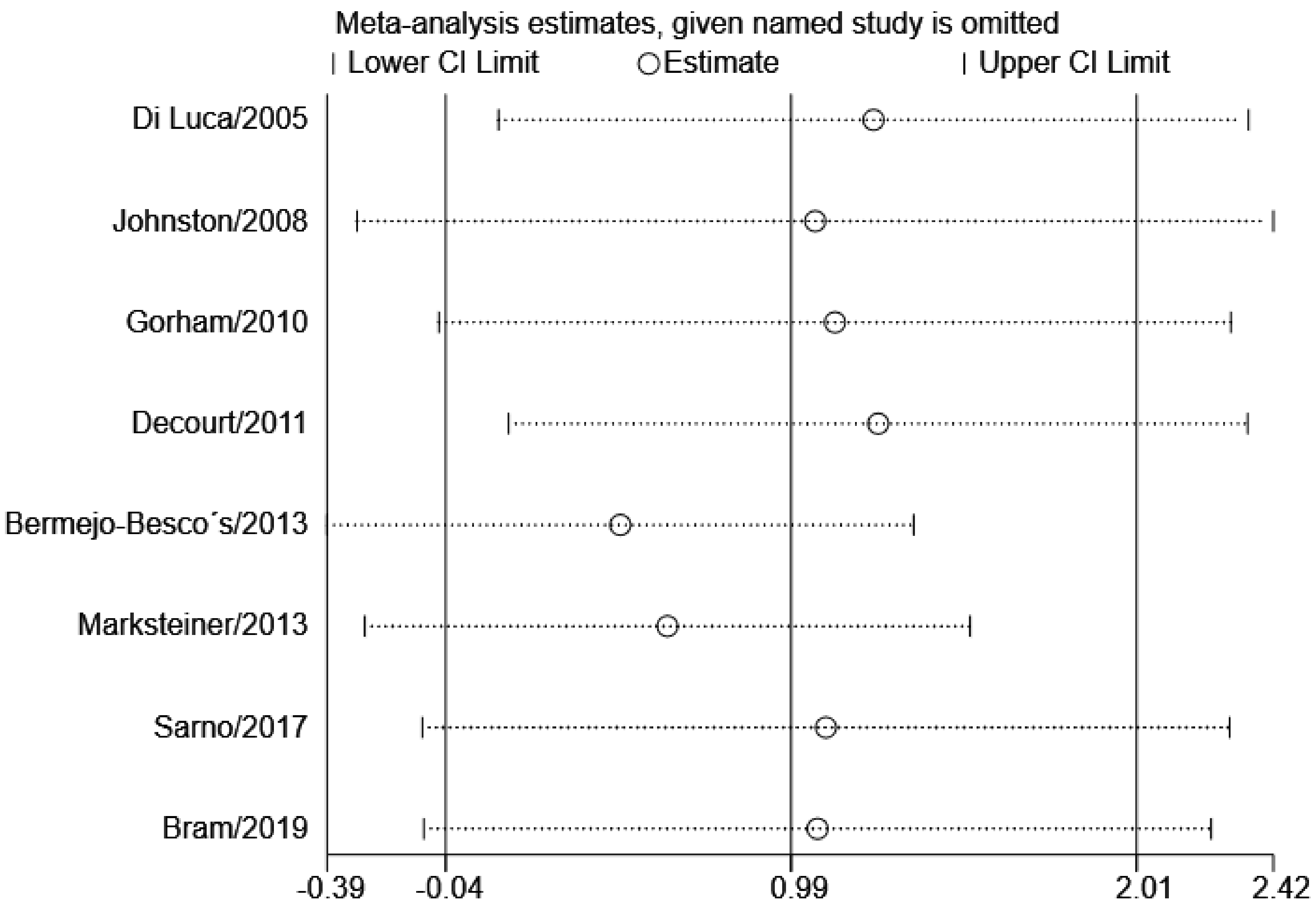

Figure S29: Sensitivity analysis for BACE-1

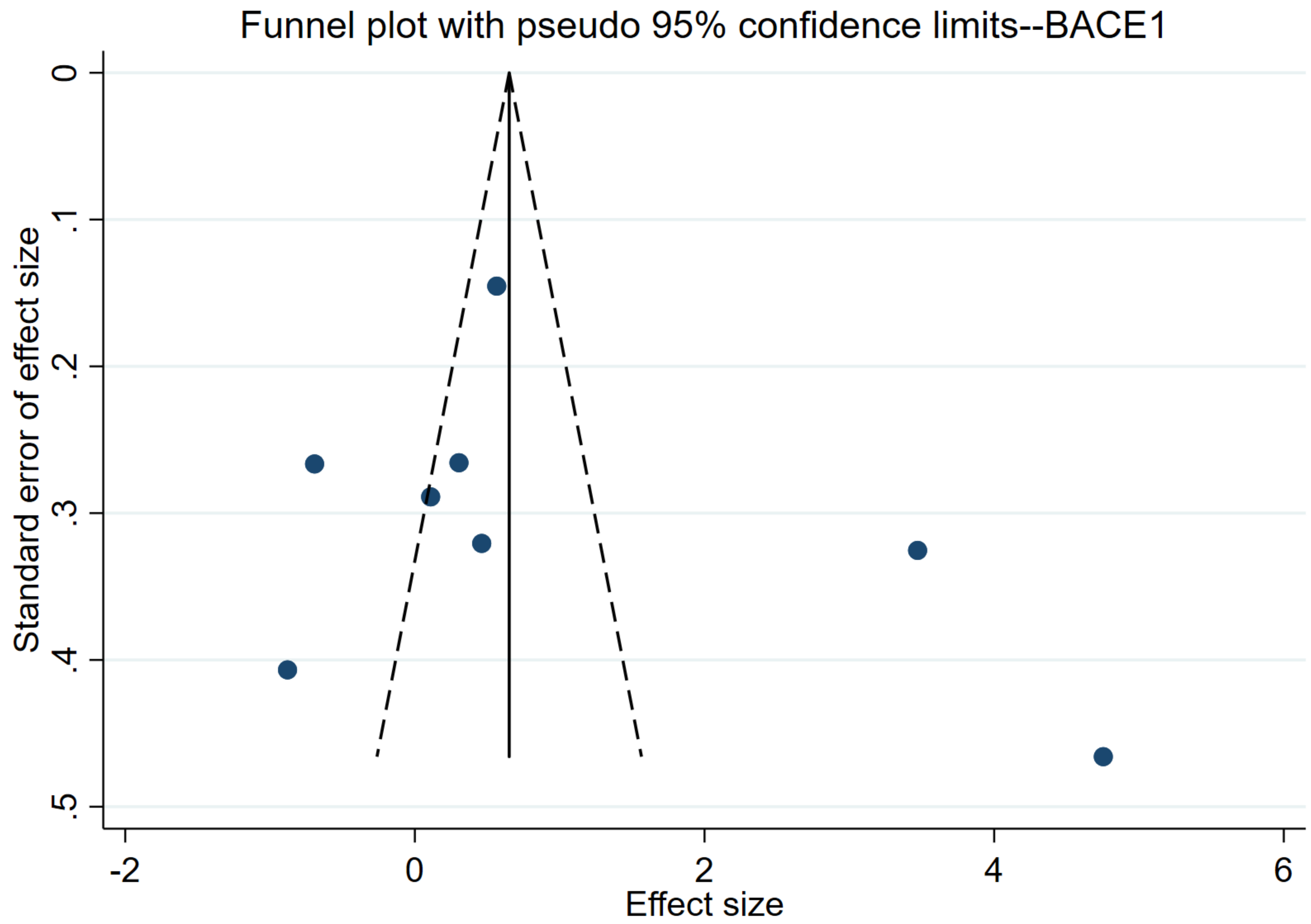

Figure S30: funnel plot of BCEA-1; Egger's test: p > 0.05

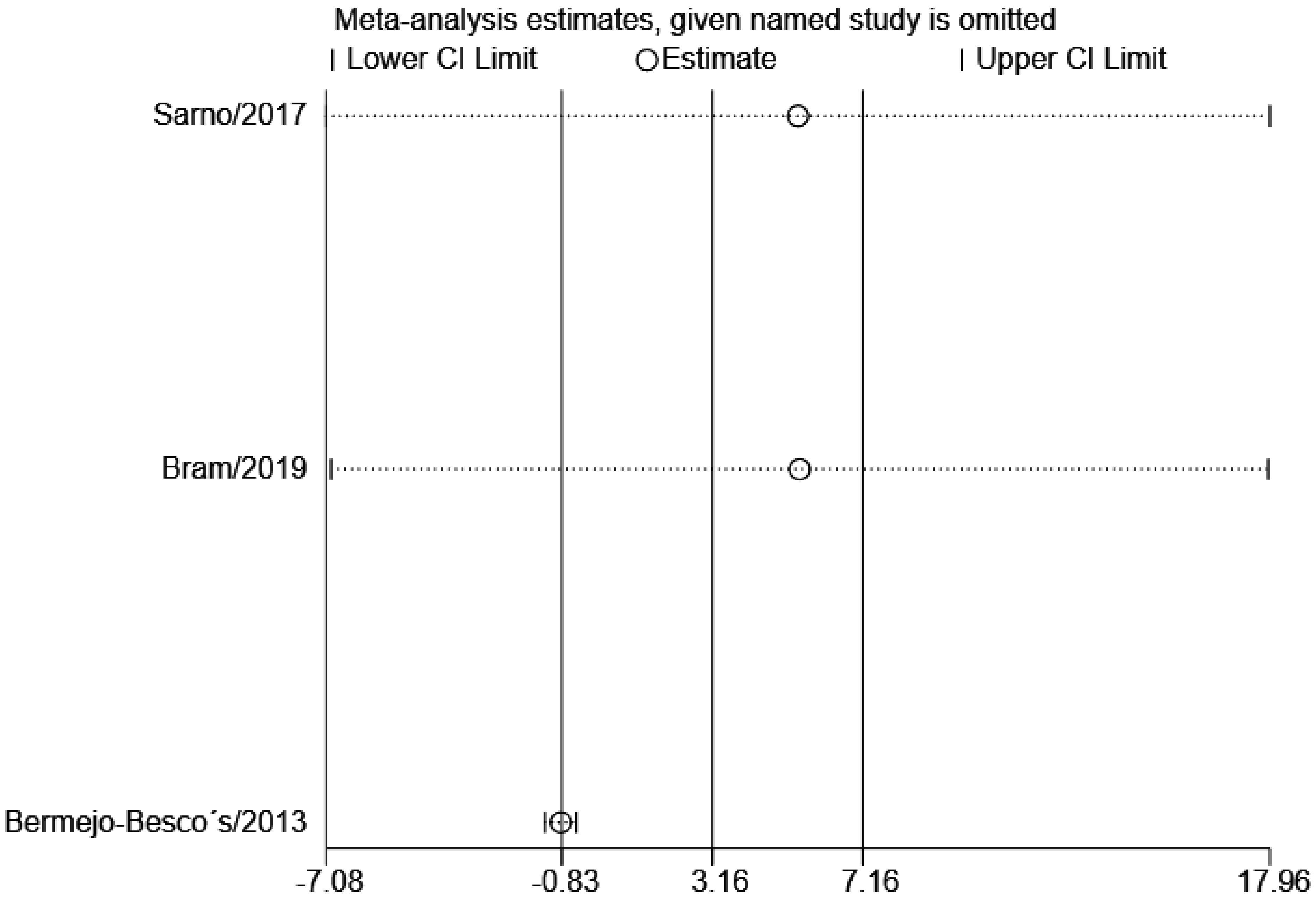

Figure S31: Sensitivity analysis for PSEN-1

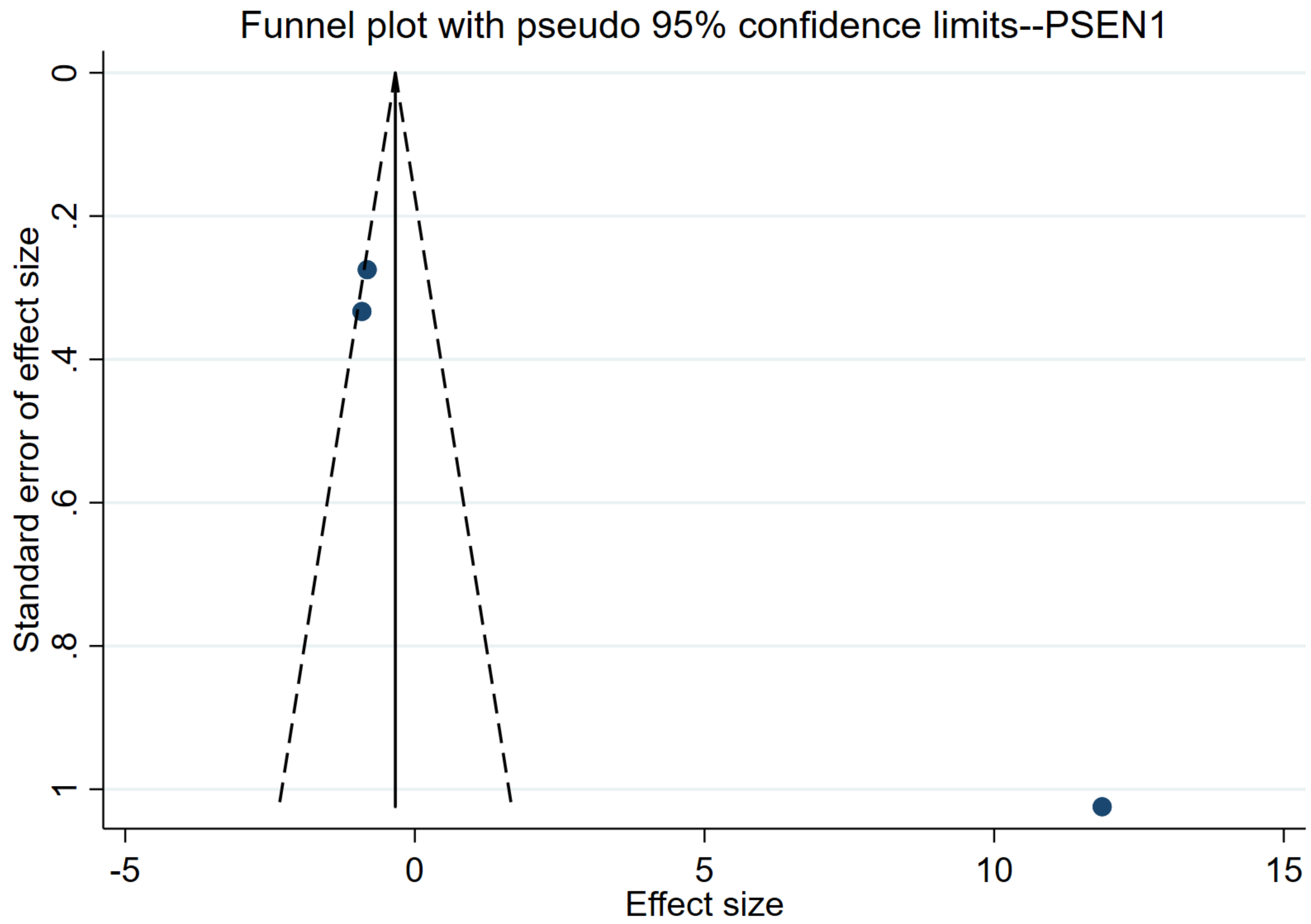

Figure S32: funnel plot of PSEN-1; Egger's test: $p > 0.05$

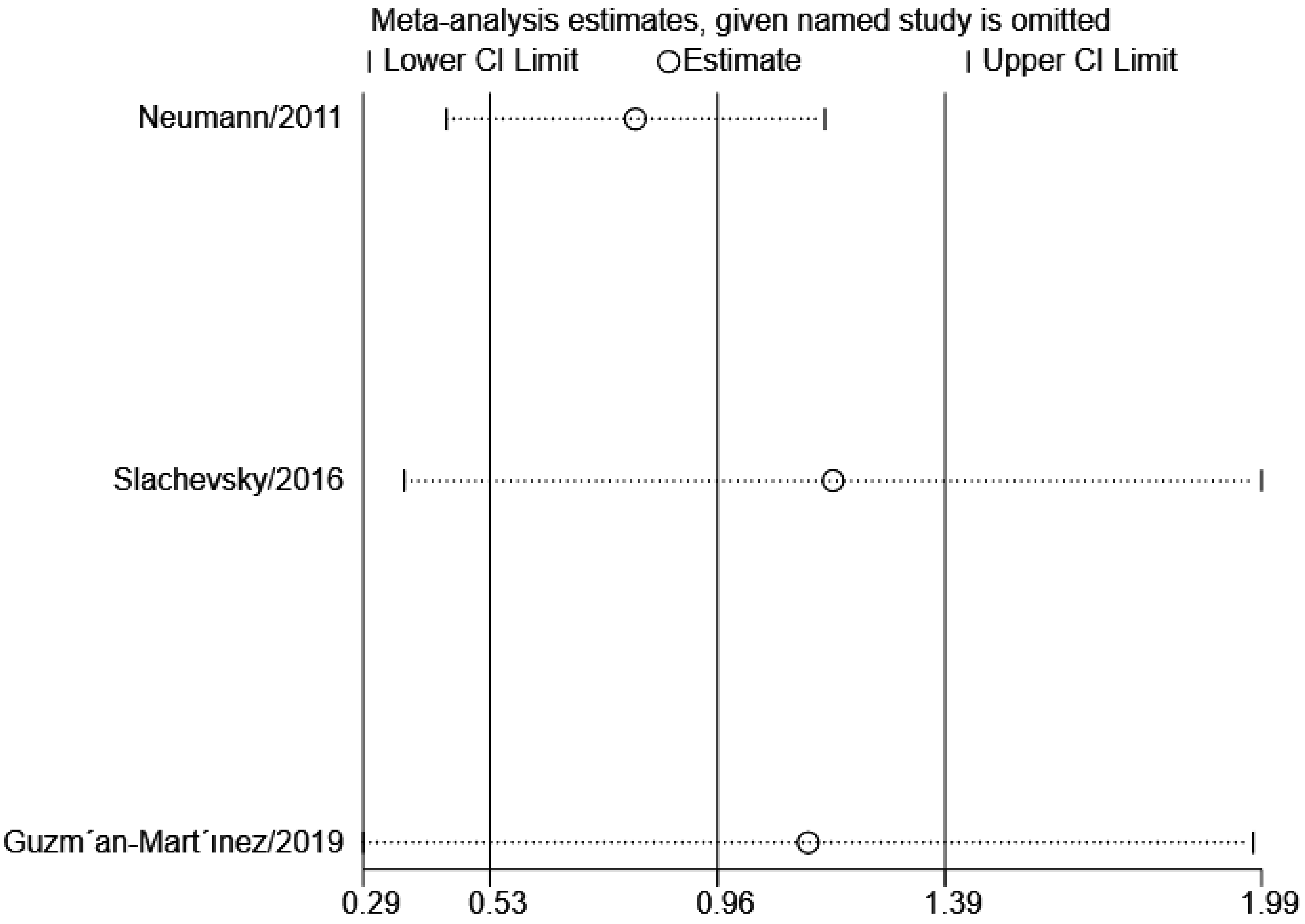

Figure S33: Sensitivity analysis for HMWtau/LMWtau

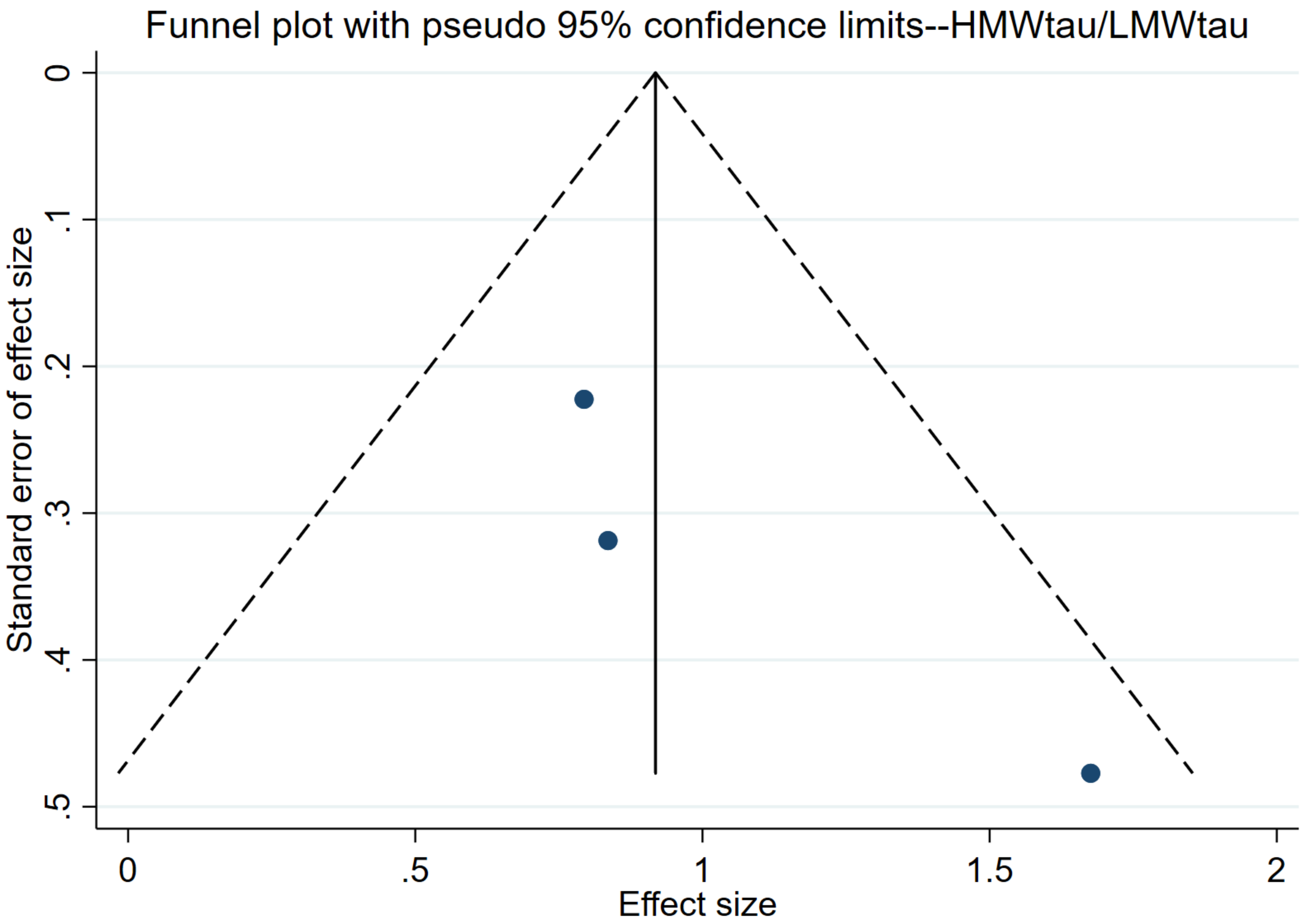

Figure S34: funnel plot of HMWtau/LMWtau; Egger's test: $p > 0.05$

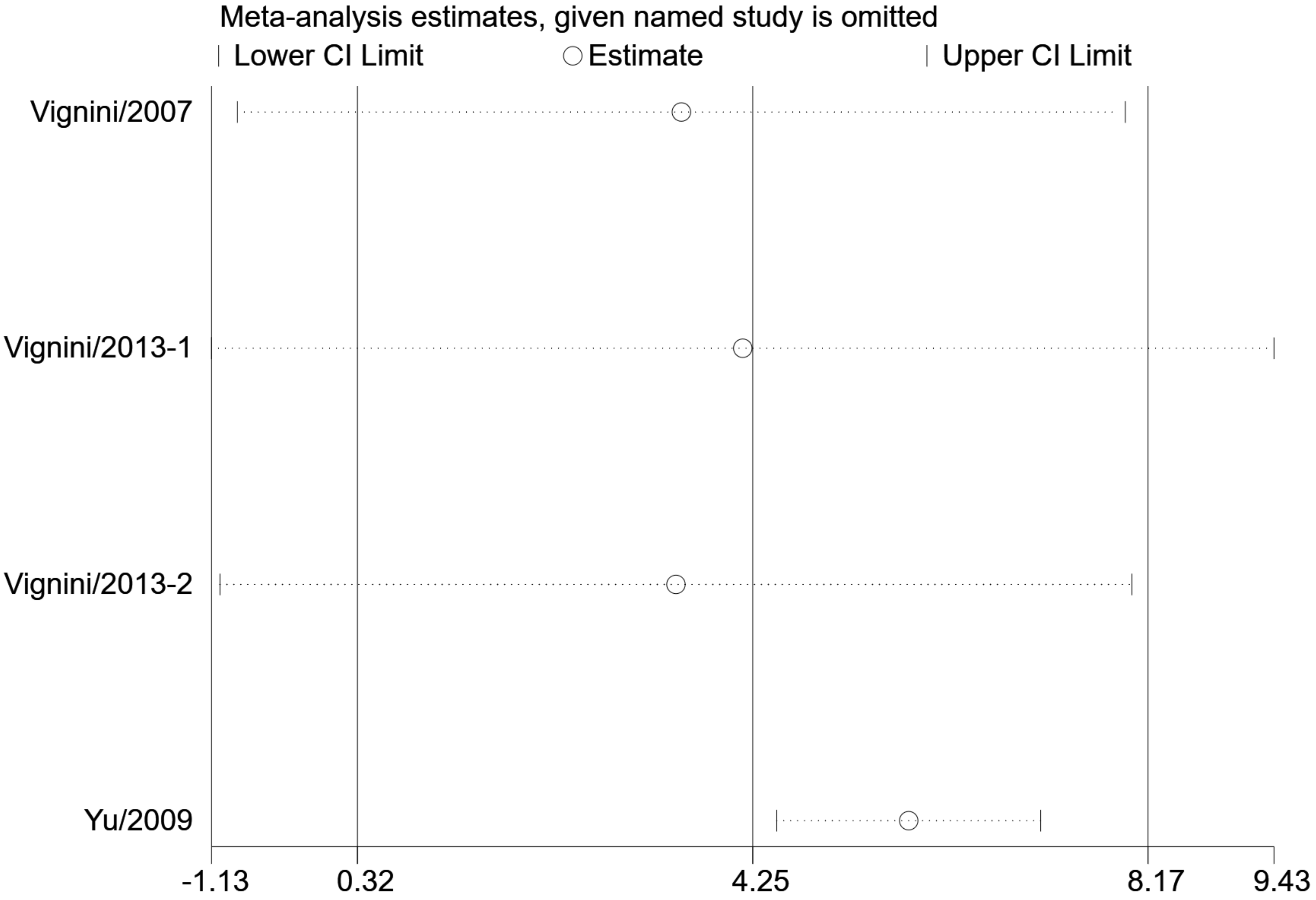

Figure S35: Sensitivity analysis for NO production

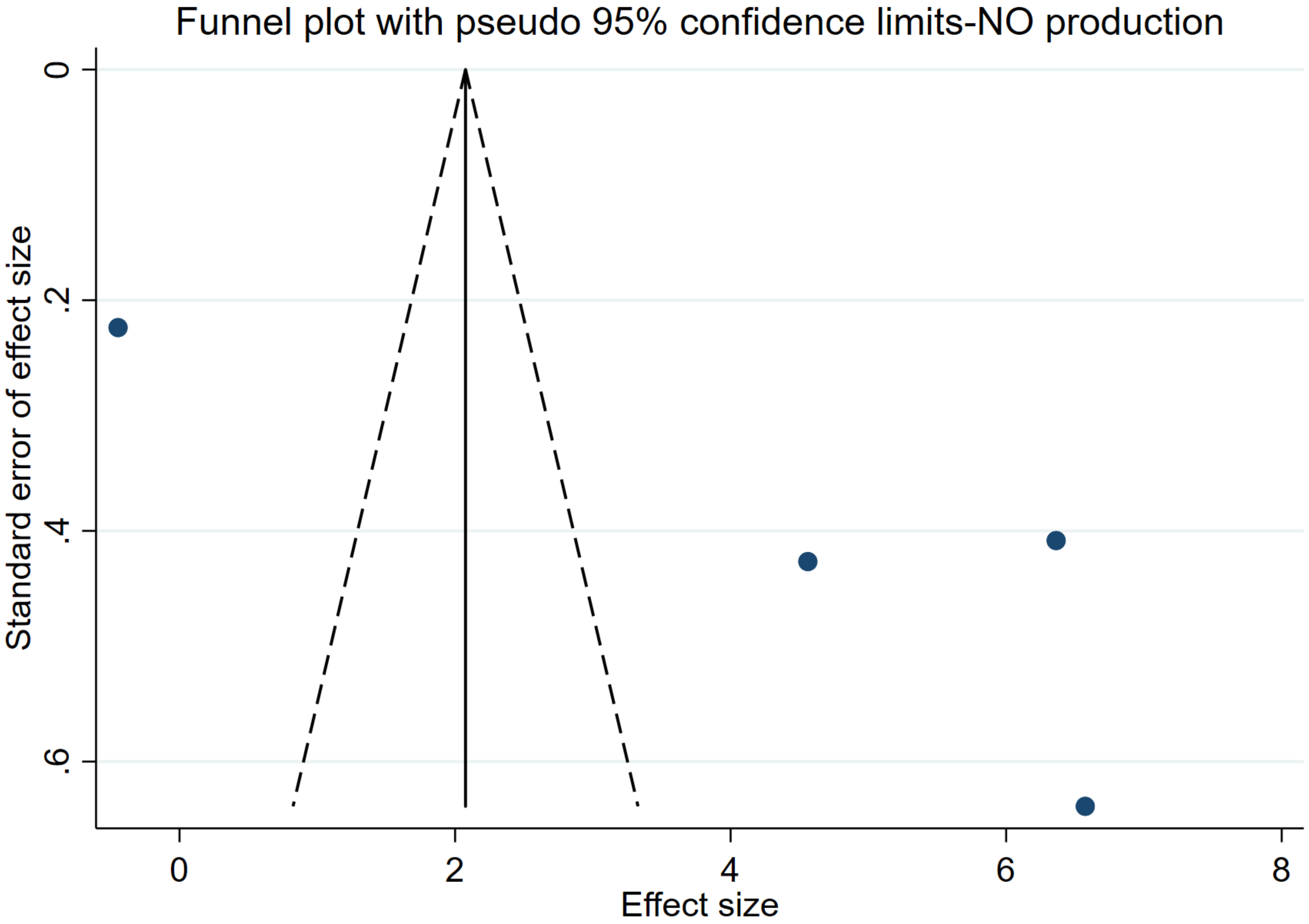

Figure S36: funnel plot of NO production; Egger's test: $p > 0.05$

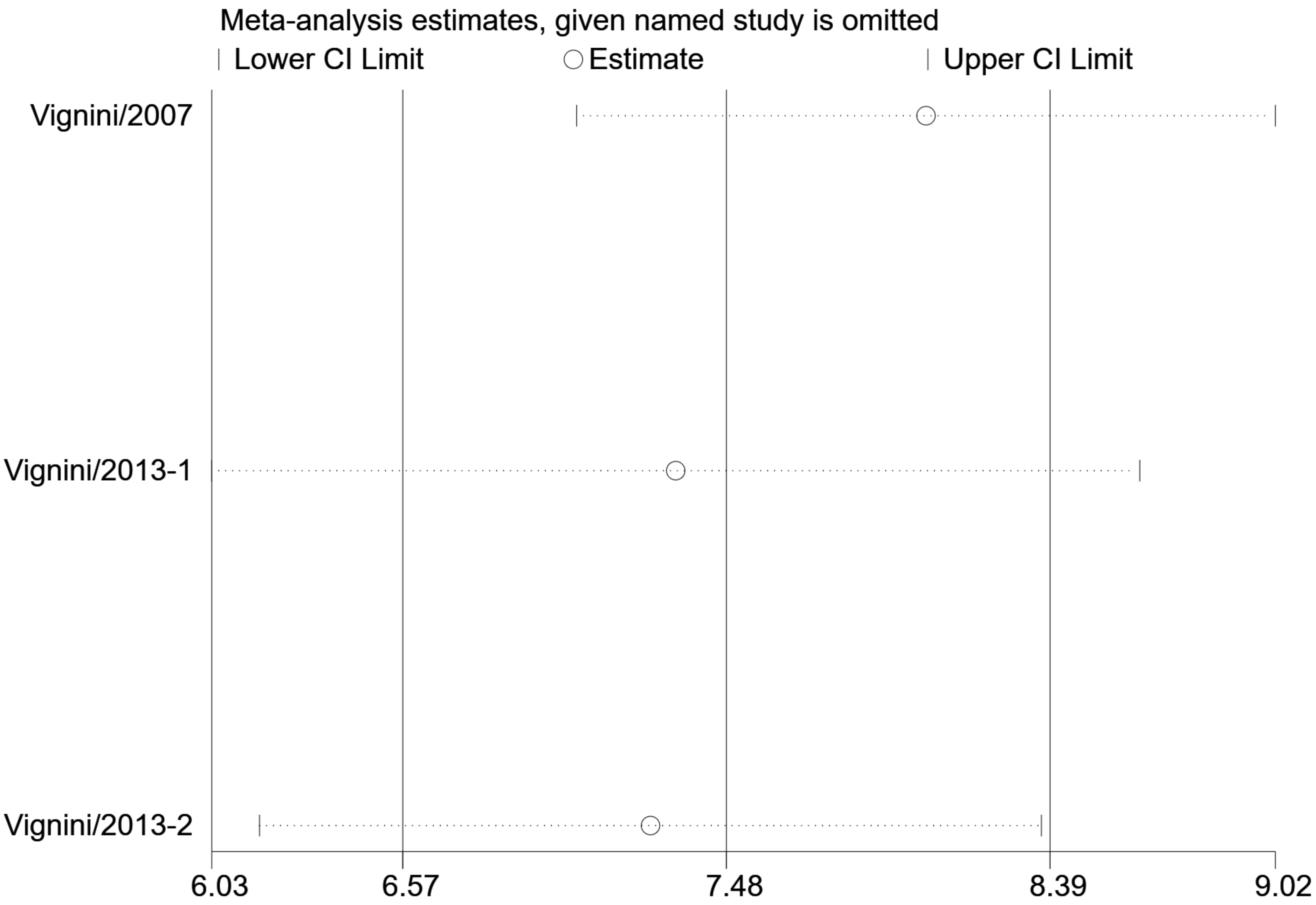

Figure S37: Sensitivity analysis for ONOO- production

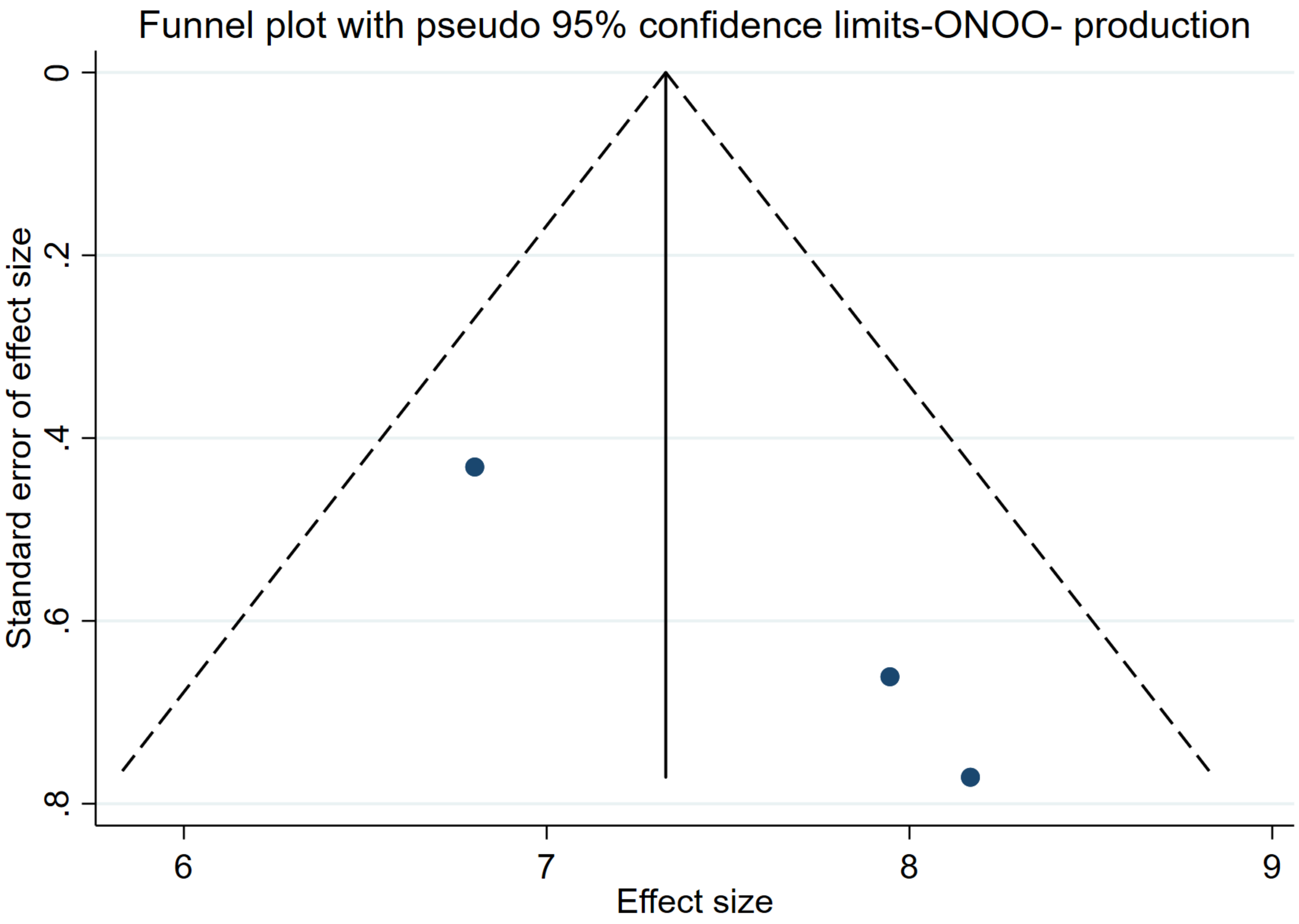

Figure S38: funnel plot of ONOO- production; Egger's test: $p > 0.05$

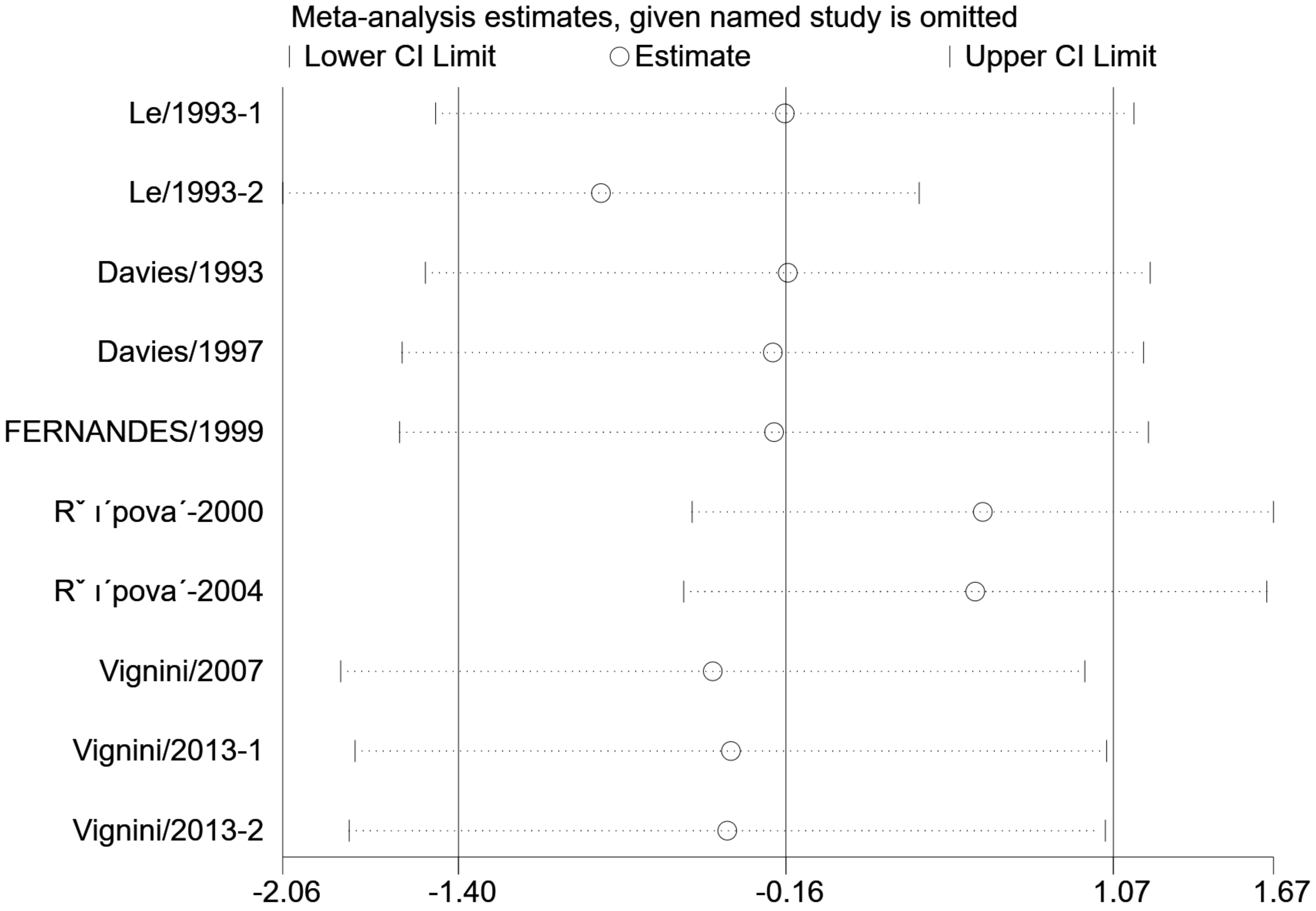

Figure S39: Sensitivity analysis for Ca^{2+}

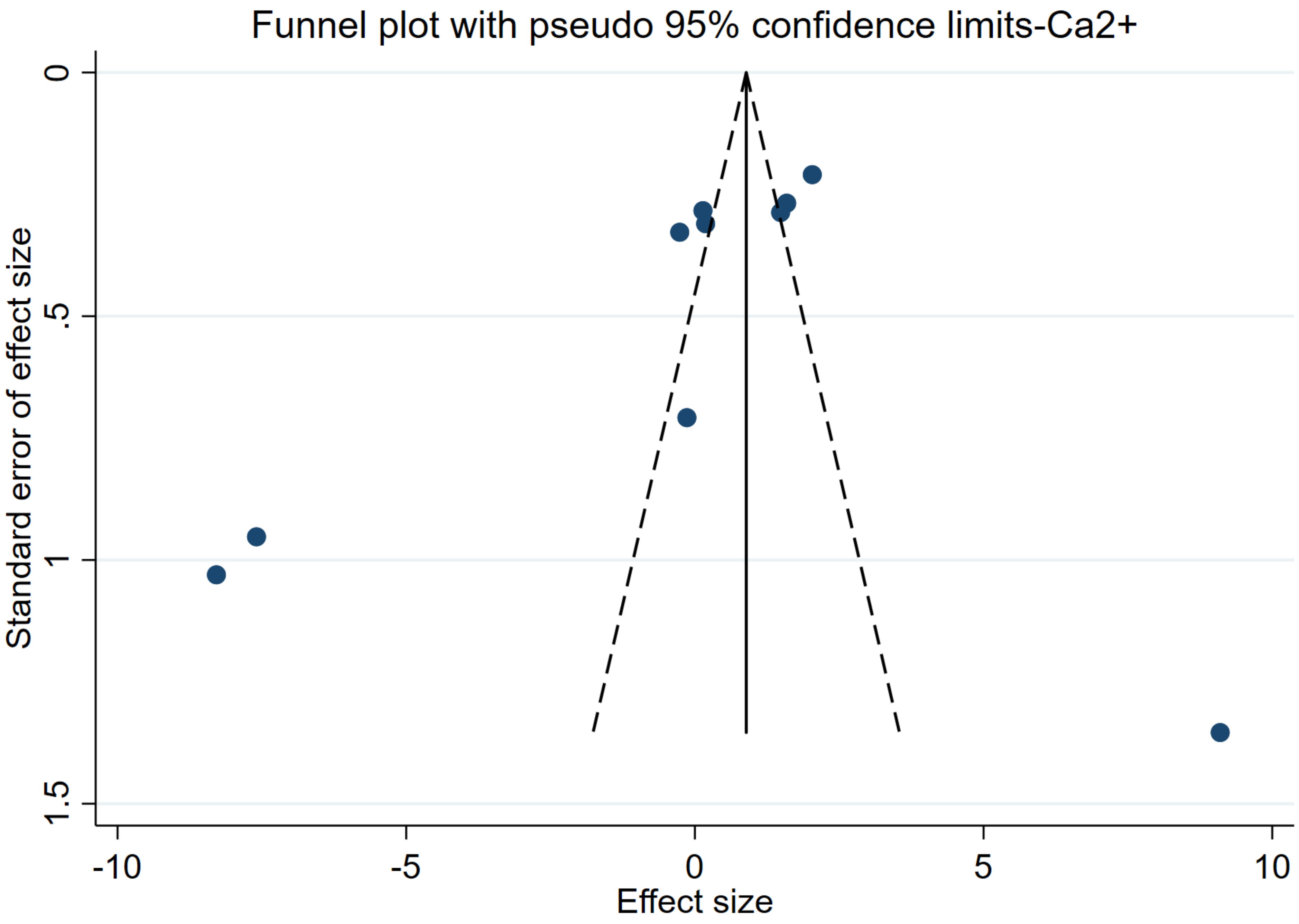

Figure S40: funnel plot of Ca^{2+}; Egger's test: $p > 0.05$

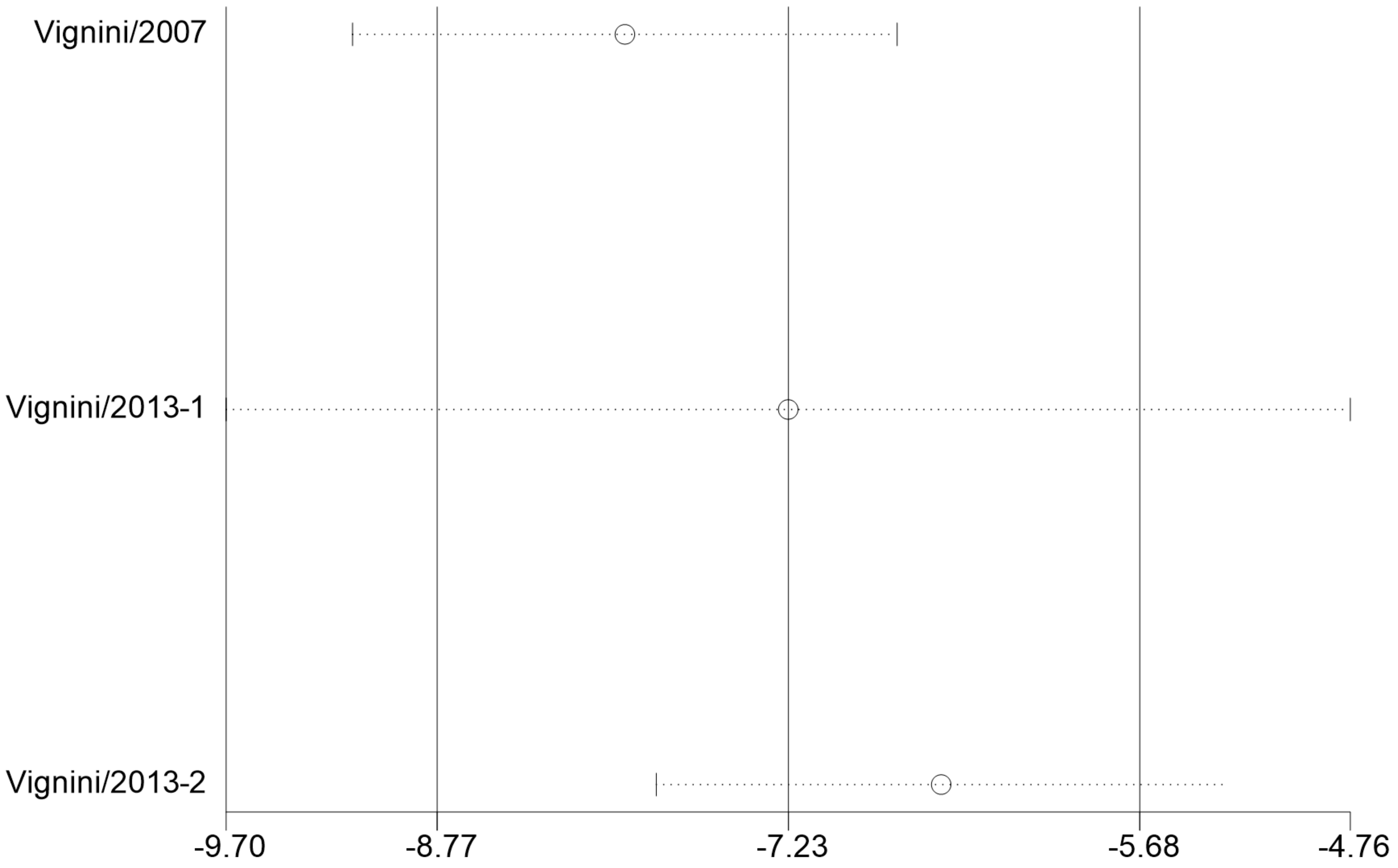

Figure S41: Sensitivity analysis for Na+-K+ -ATPase

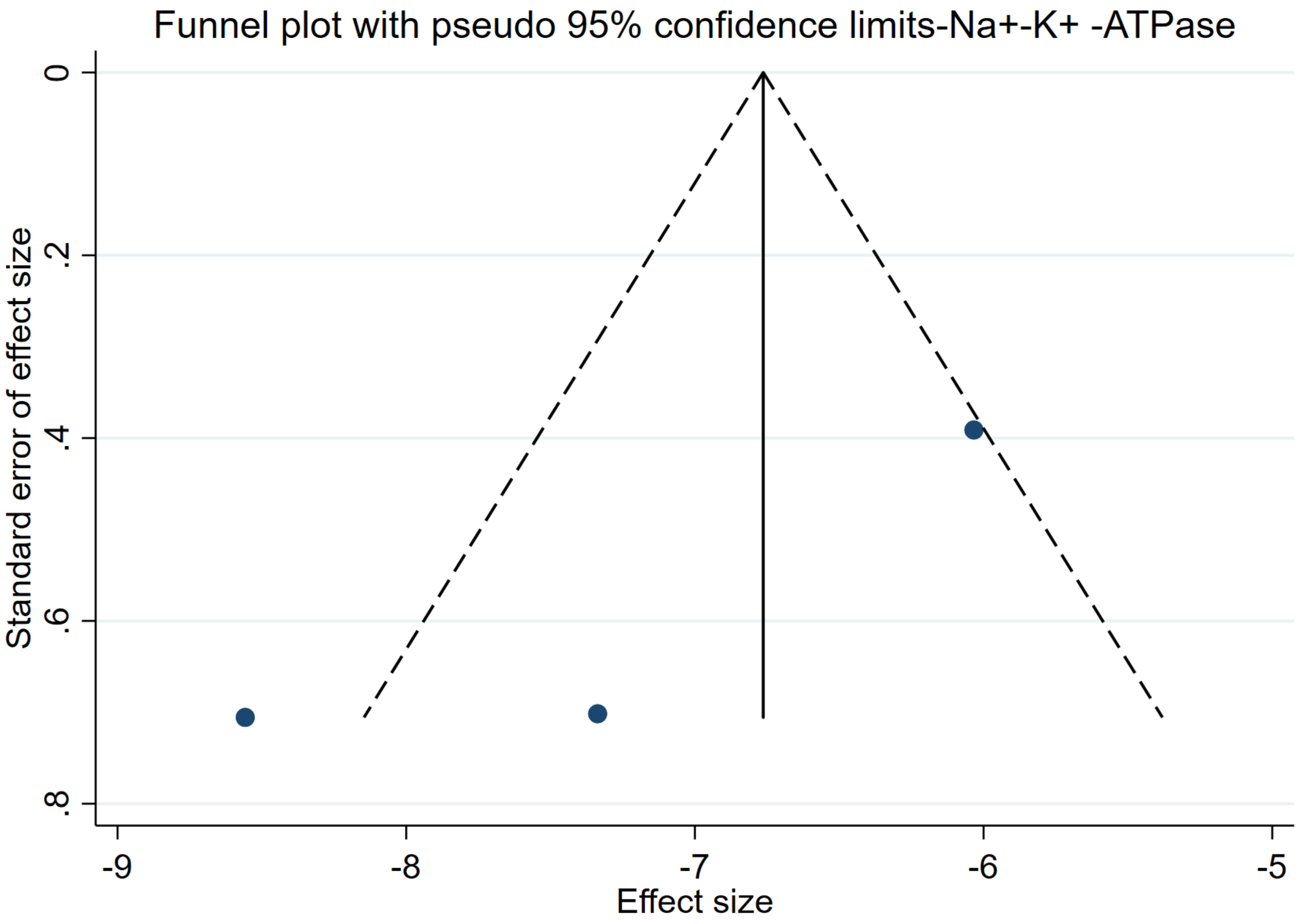

Figure S42: funnel plot of Na^{+}-K^{+} -ATPase; Egger's test: $p > 0.05$

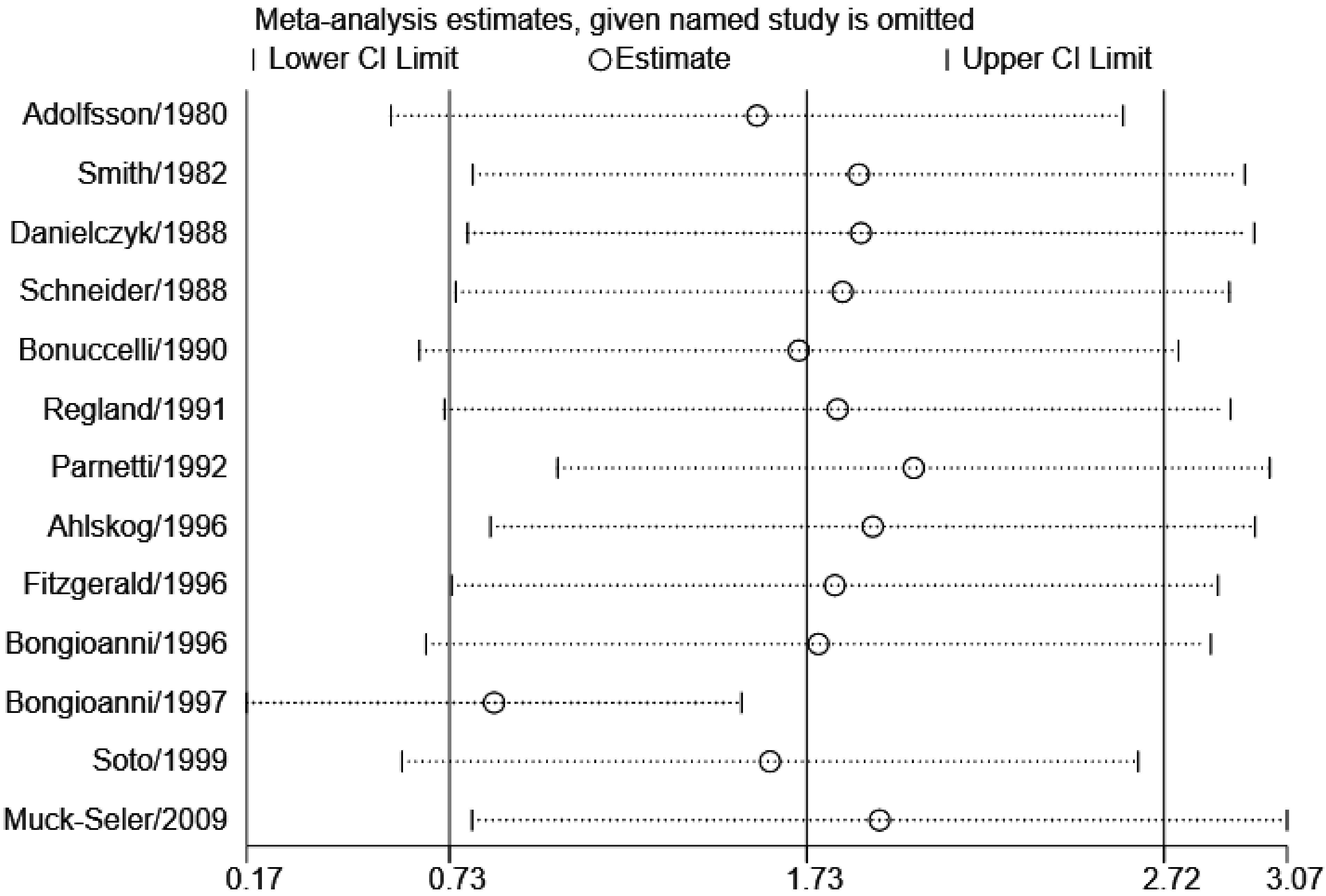

Figure S43: Sensitivity analysis for MAO-B

MAO-B

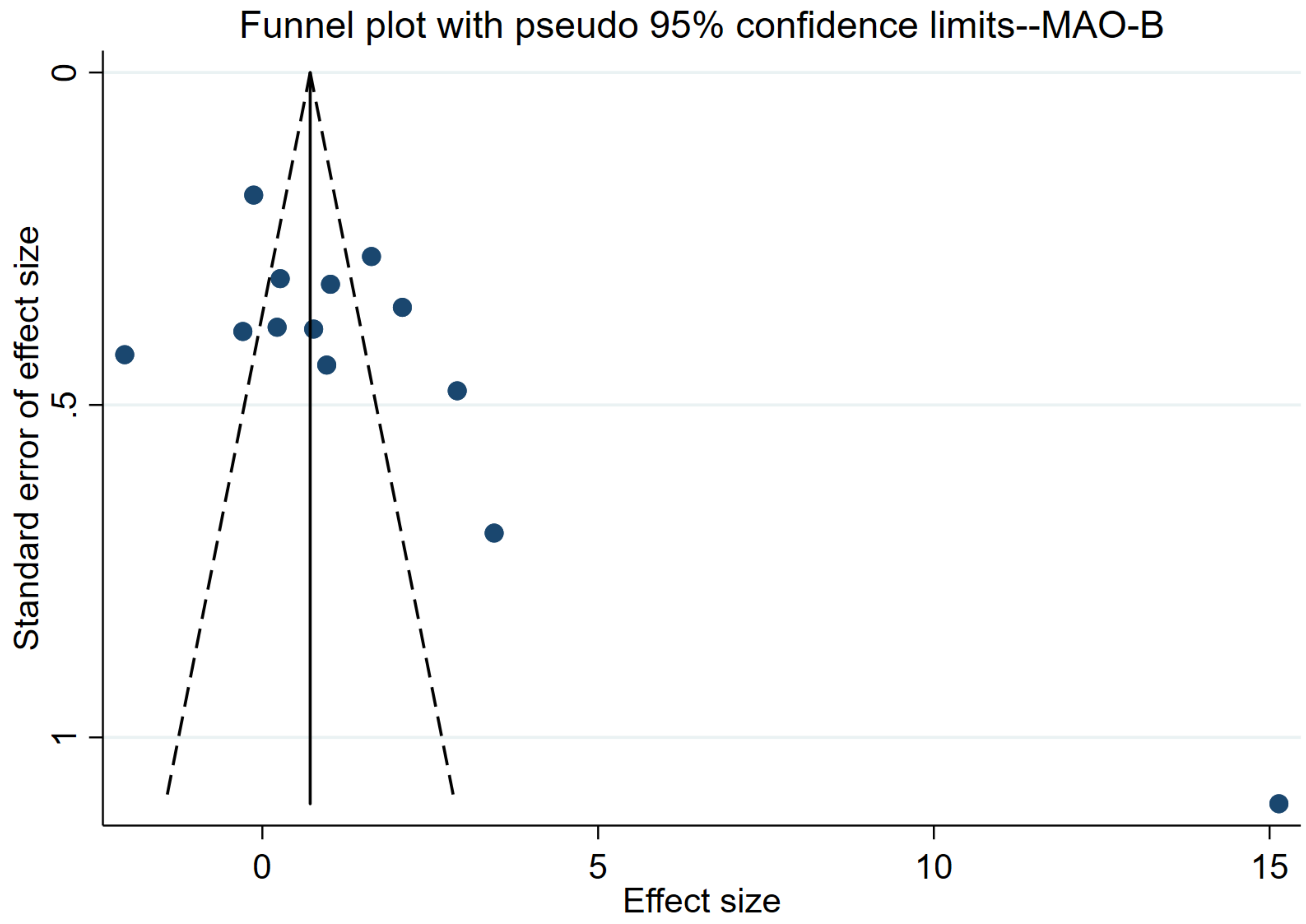

Figure S44: funnel plot of MAO-B; Egger's test: p=0.025

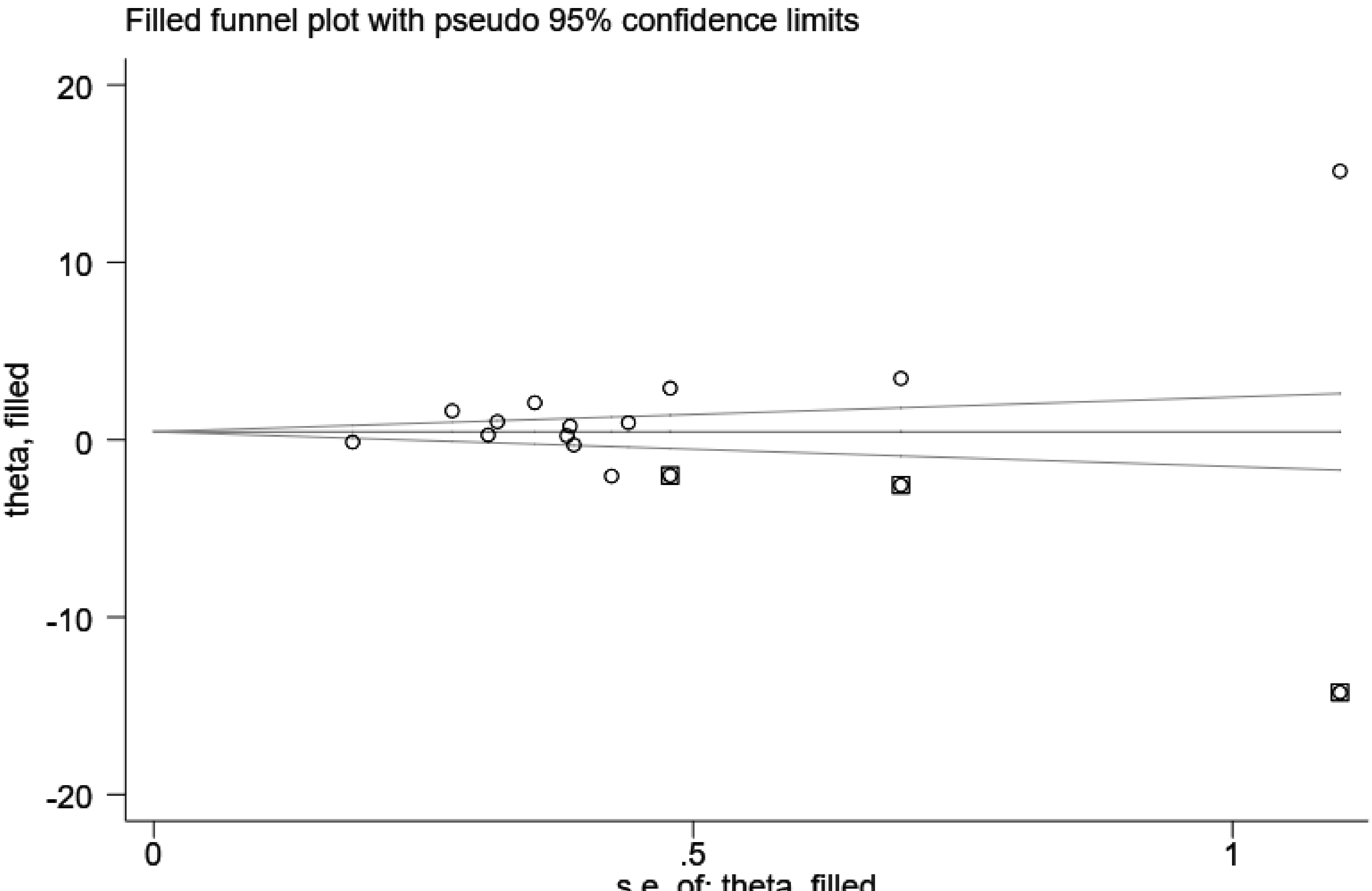

Figure S45: filled funnel plot of MAO-B

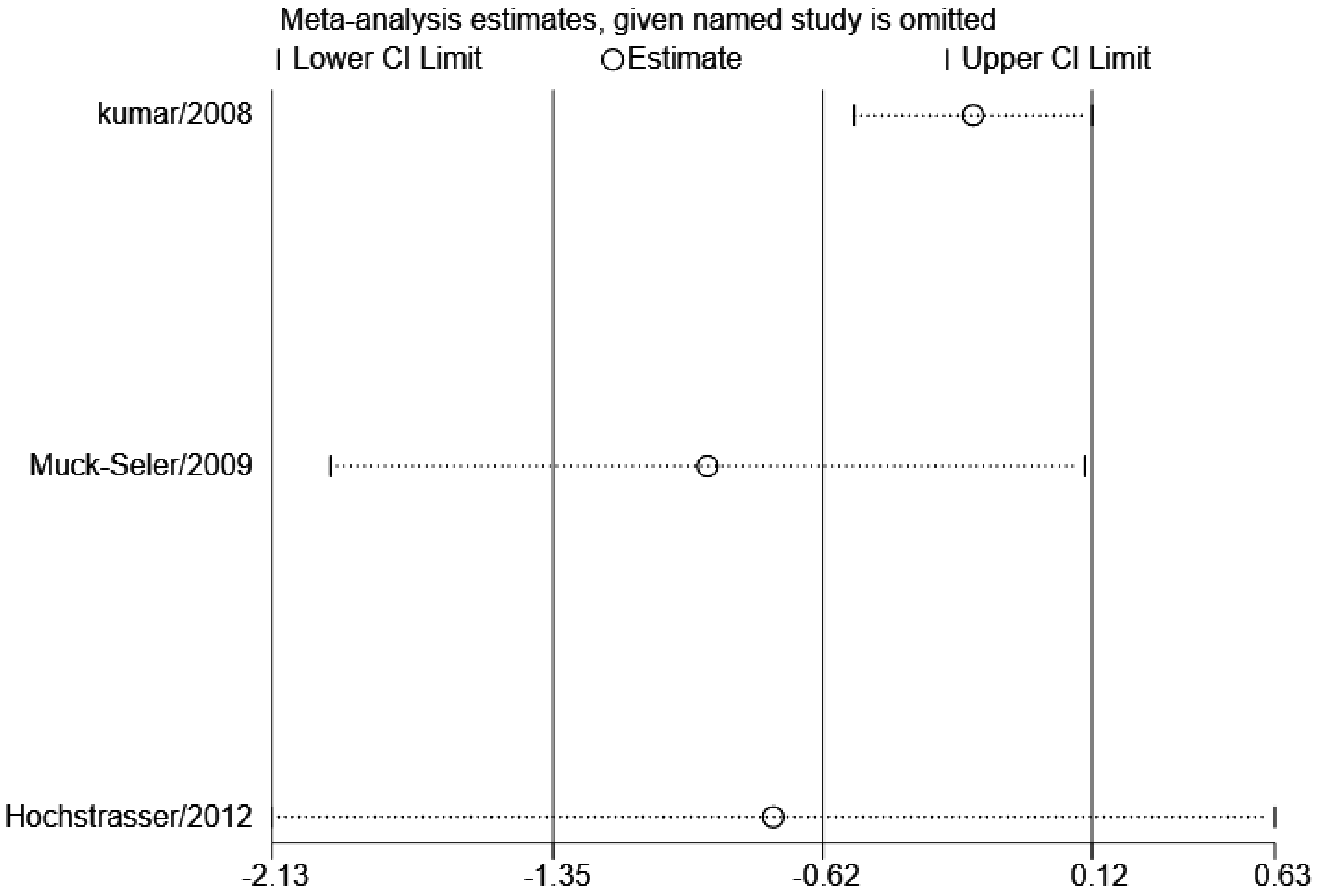

Figure S46: Sensitivity analysis for 5-HT

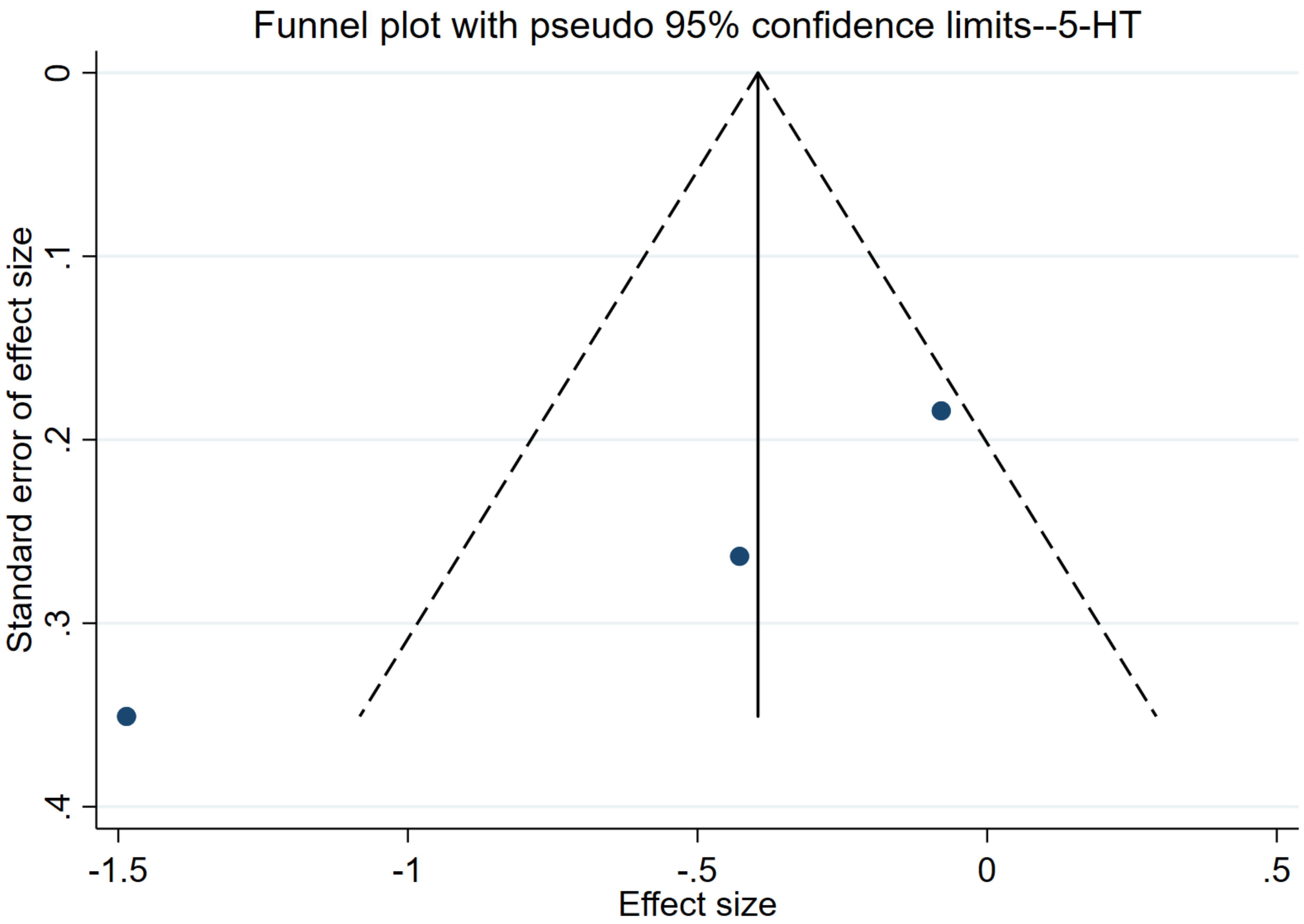

Figure S47: funnel plot of 5-HT; Egger's test: P > 0.05

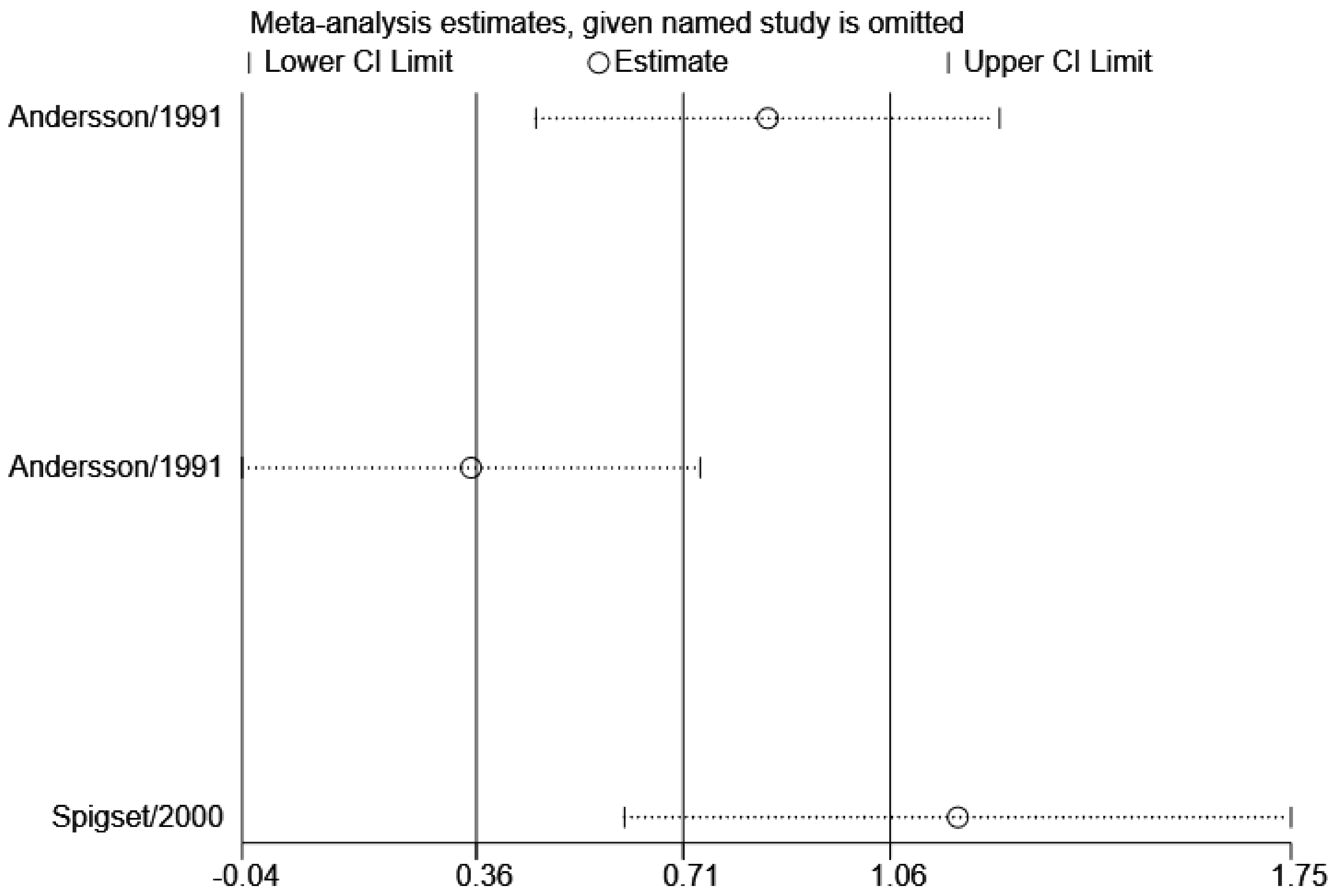

Figure S48: Sensitivity analysis for 5-HT(Bmax)

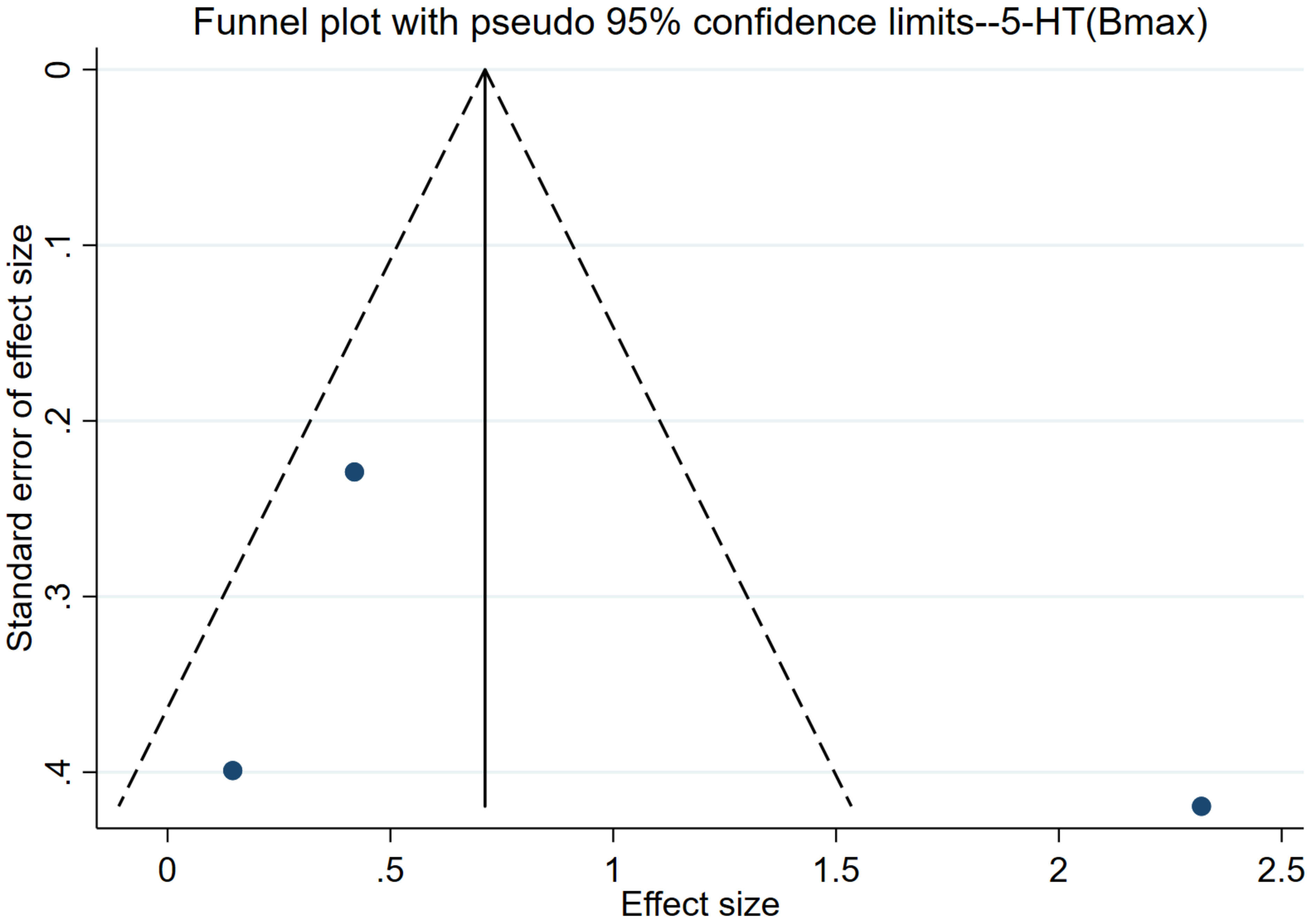

Figure S49: funnel plot of 5-HT(Bmax); Egger's test: P > 0.05

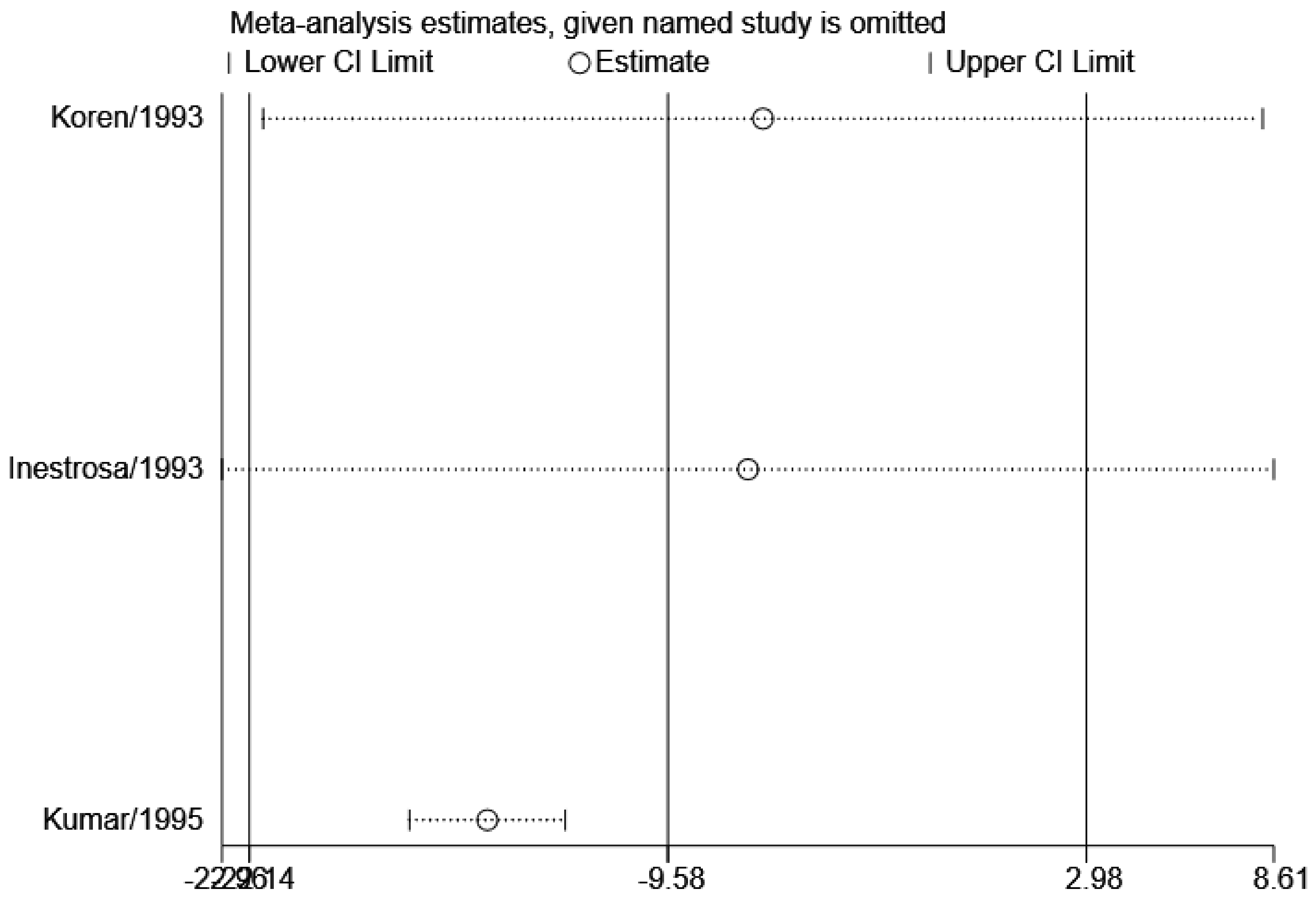

Figure S50: Sensitivity analysis for 5-HT(Vmax)

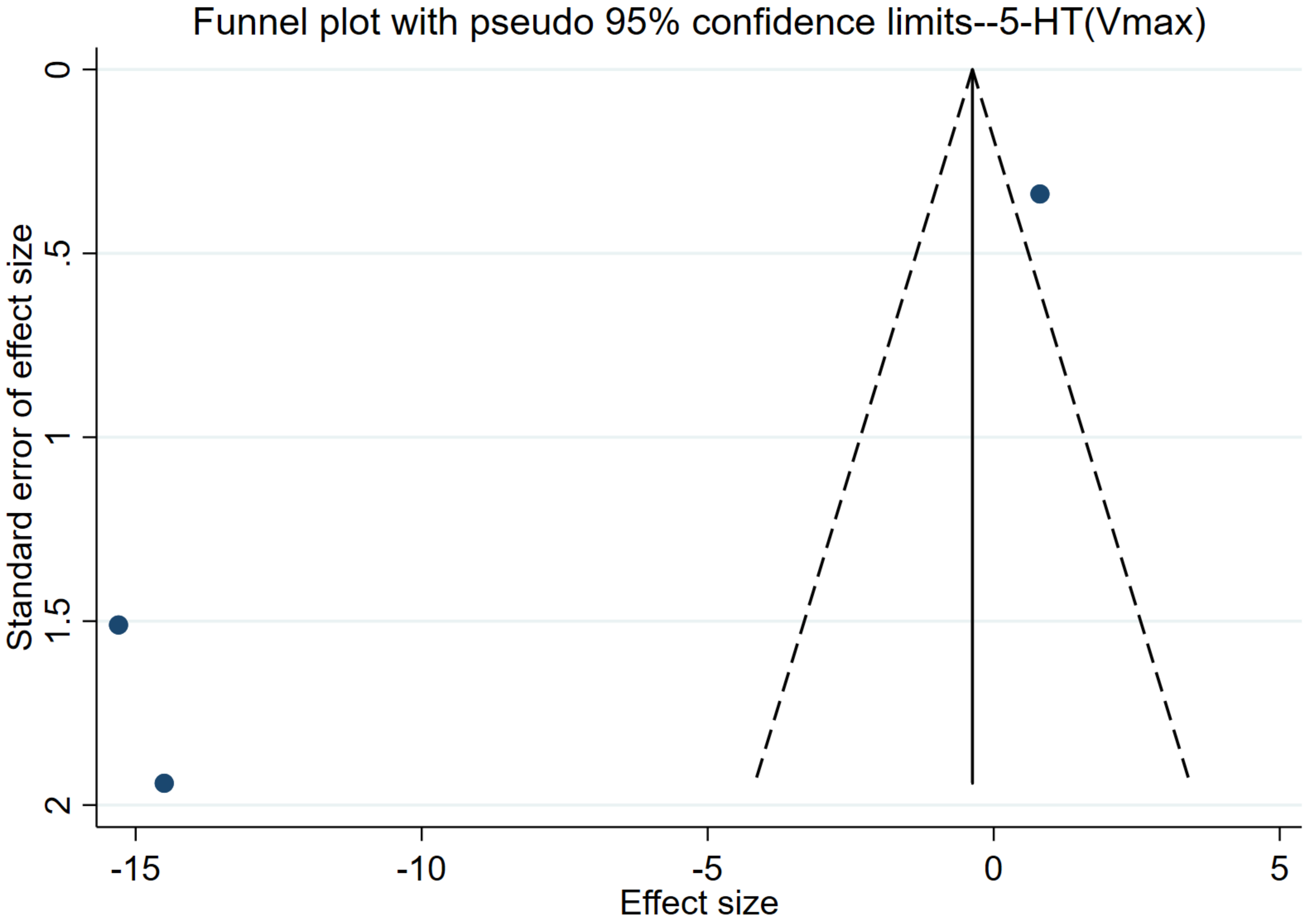

Figure S51: funnel plot of 5-HT(Vmax); Egger's test: P > 0.05

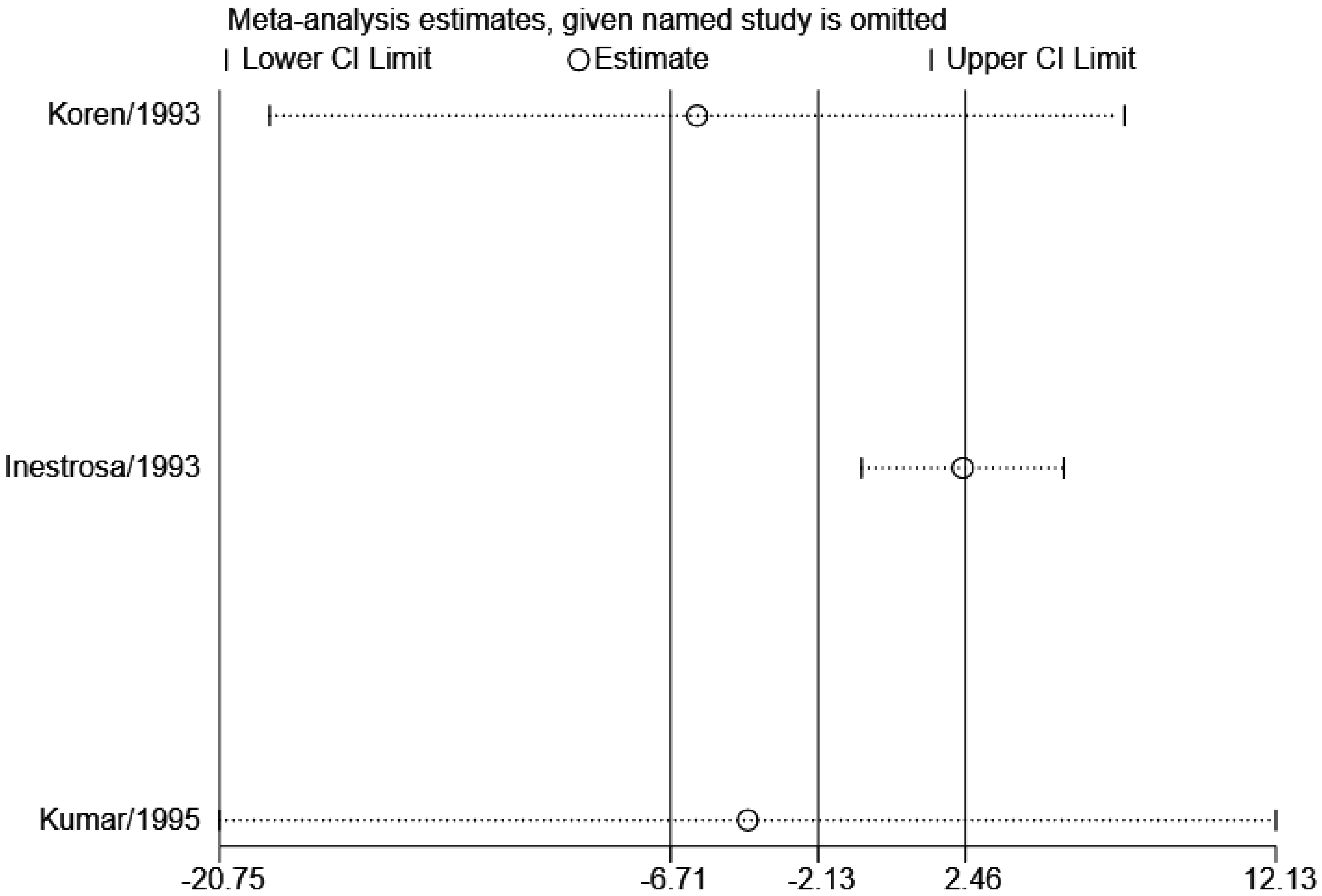

Figure S52: Sensitivity analysis for 5-HT(Km)

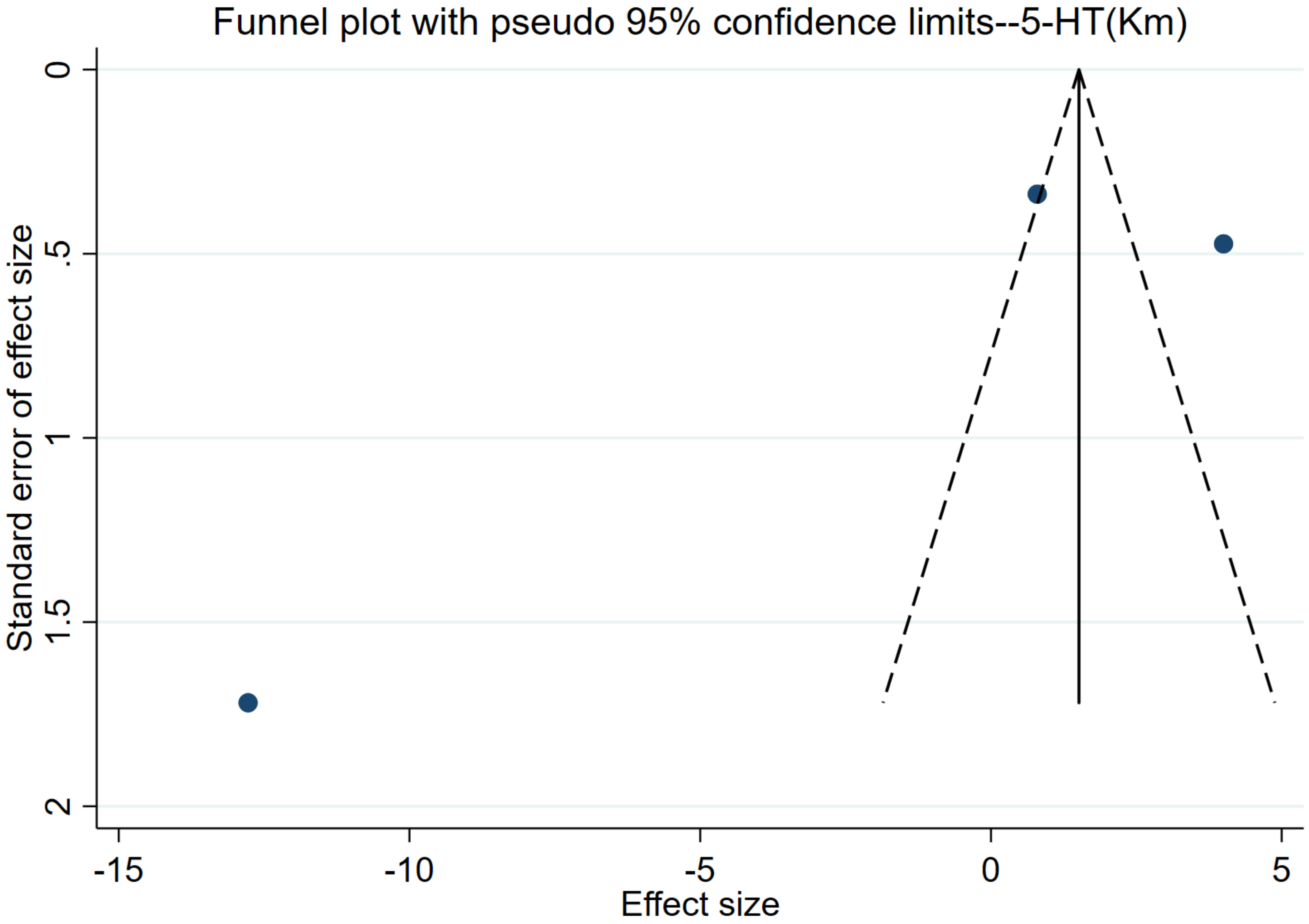

Figure S53: funnel plot of 5-HT(Km); Egger's test: P > 0.05

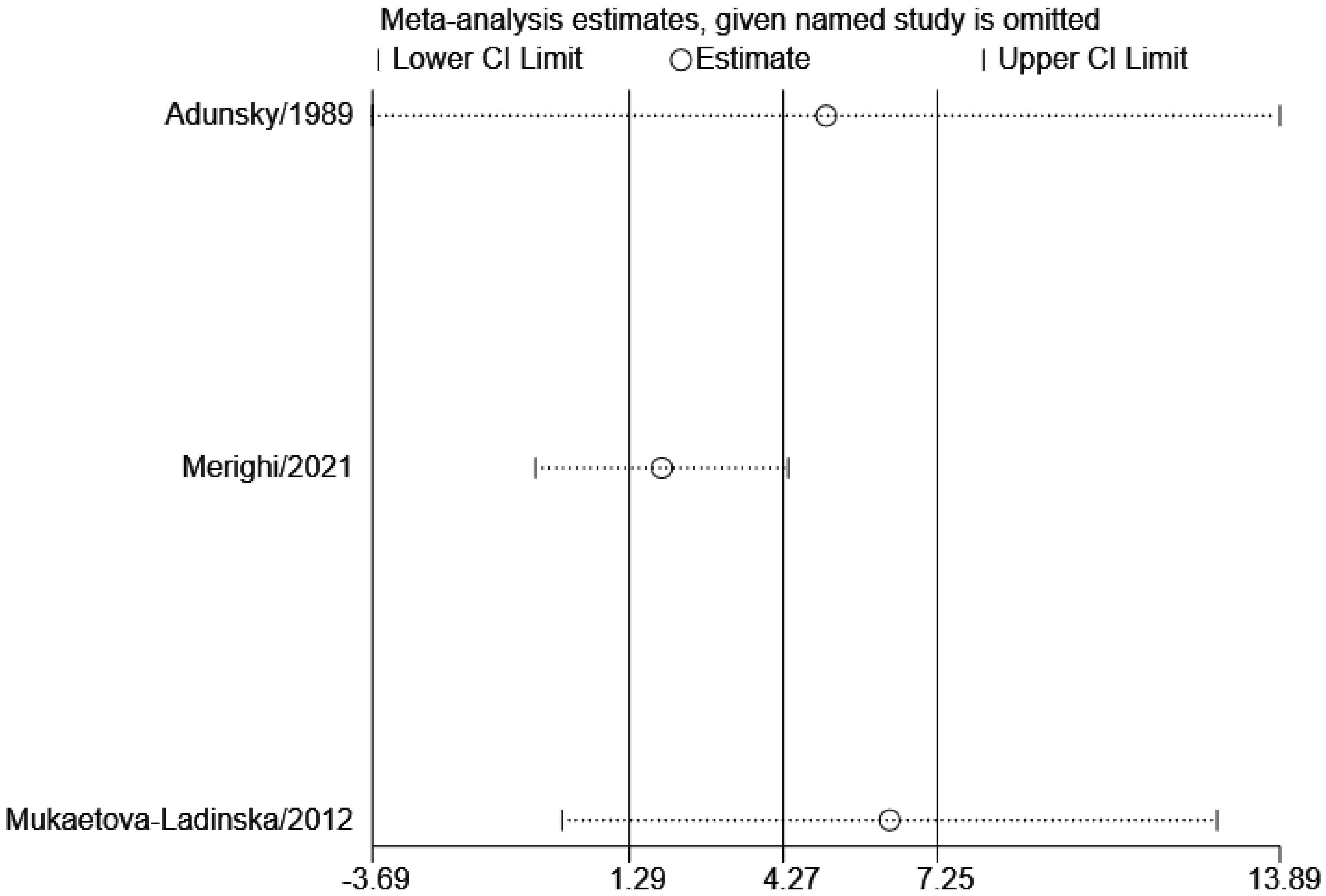

Figure S54: Sensitivity analysis for A_2Receptor

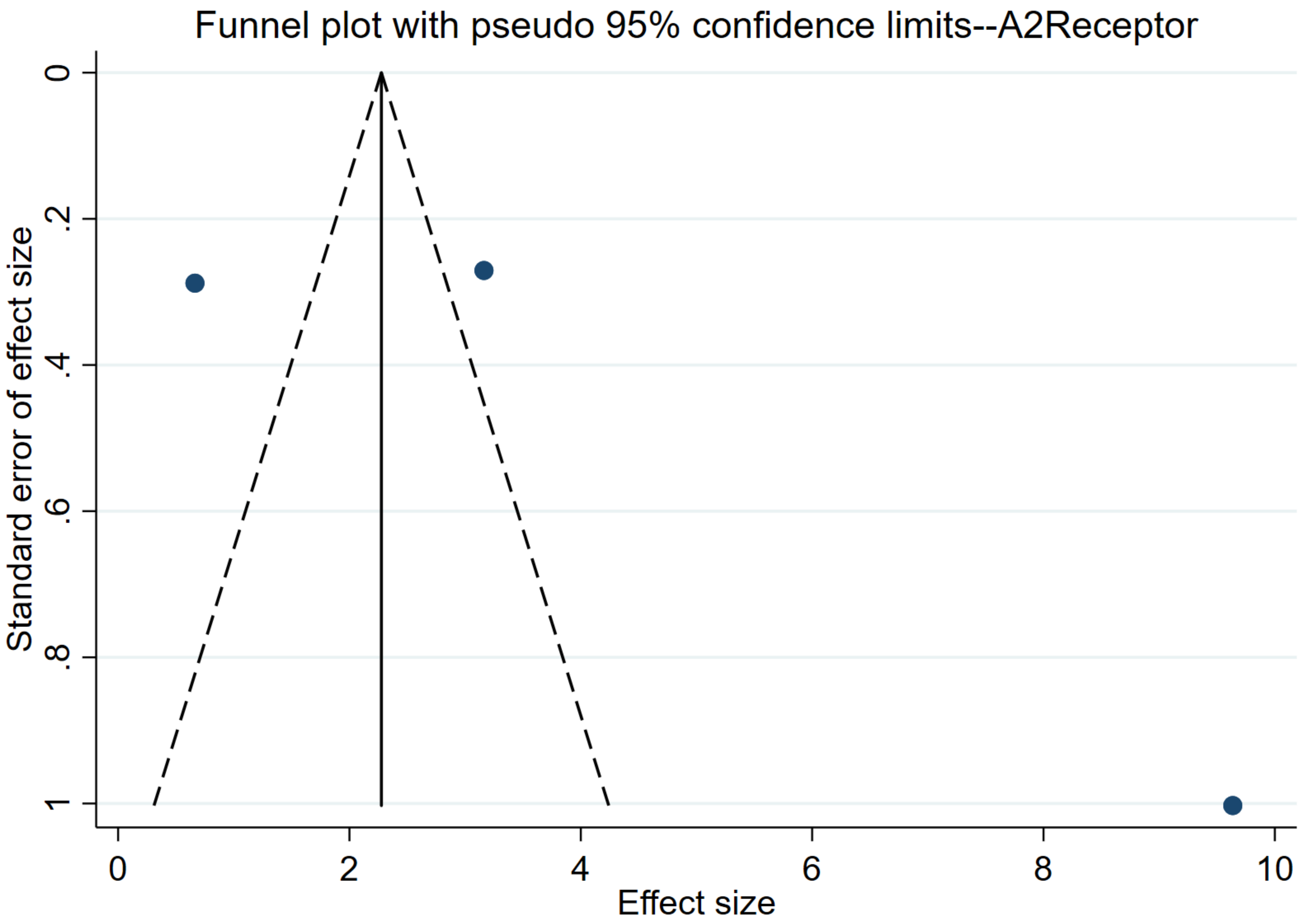

Figure S55: funnel plot of A_2Receptor; Egger's test: P > 0.05

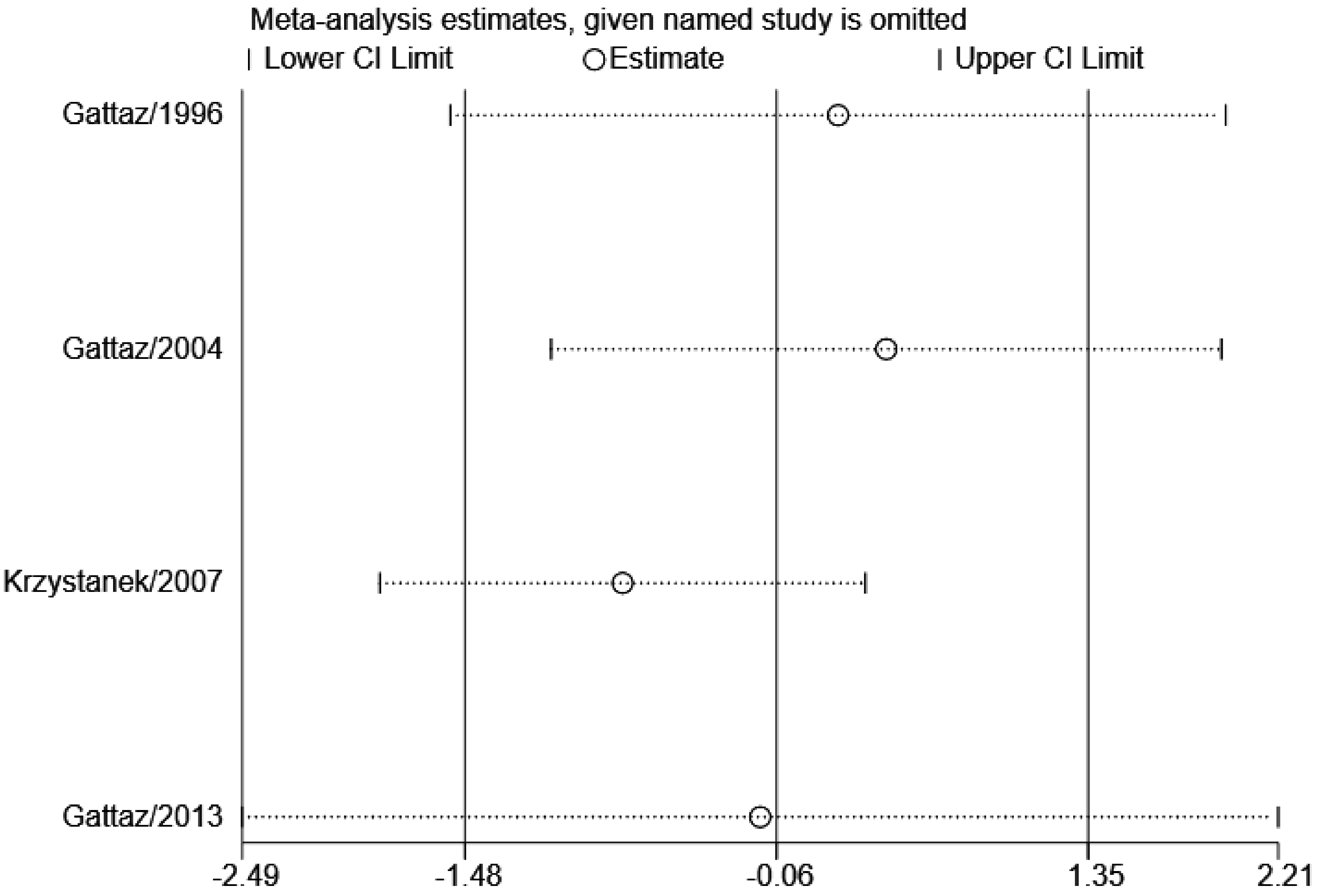

Figure S56: Sensitivity analysis for PLA_2

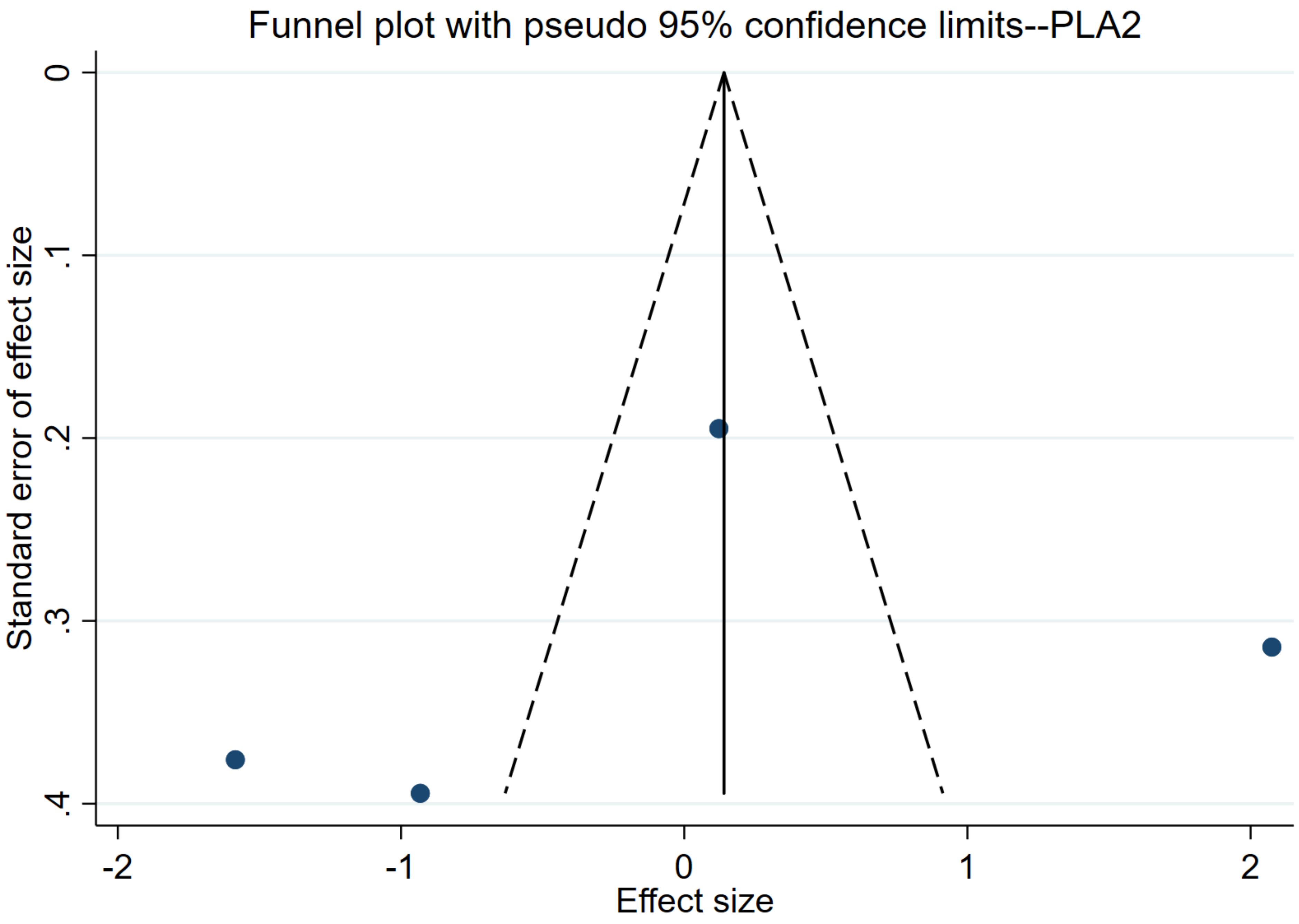

Figure S57: funnel plot of PLA_2; Egger's test: $P > 0.05$

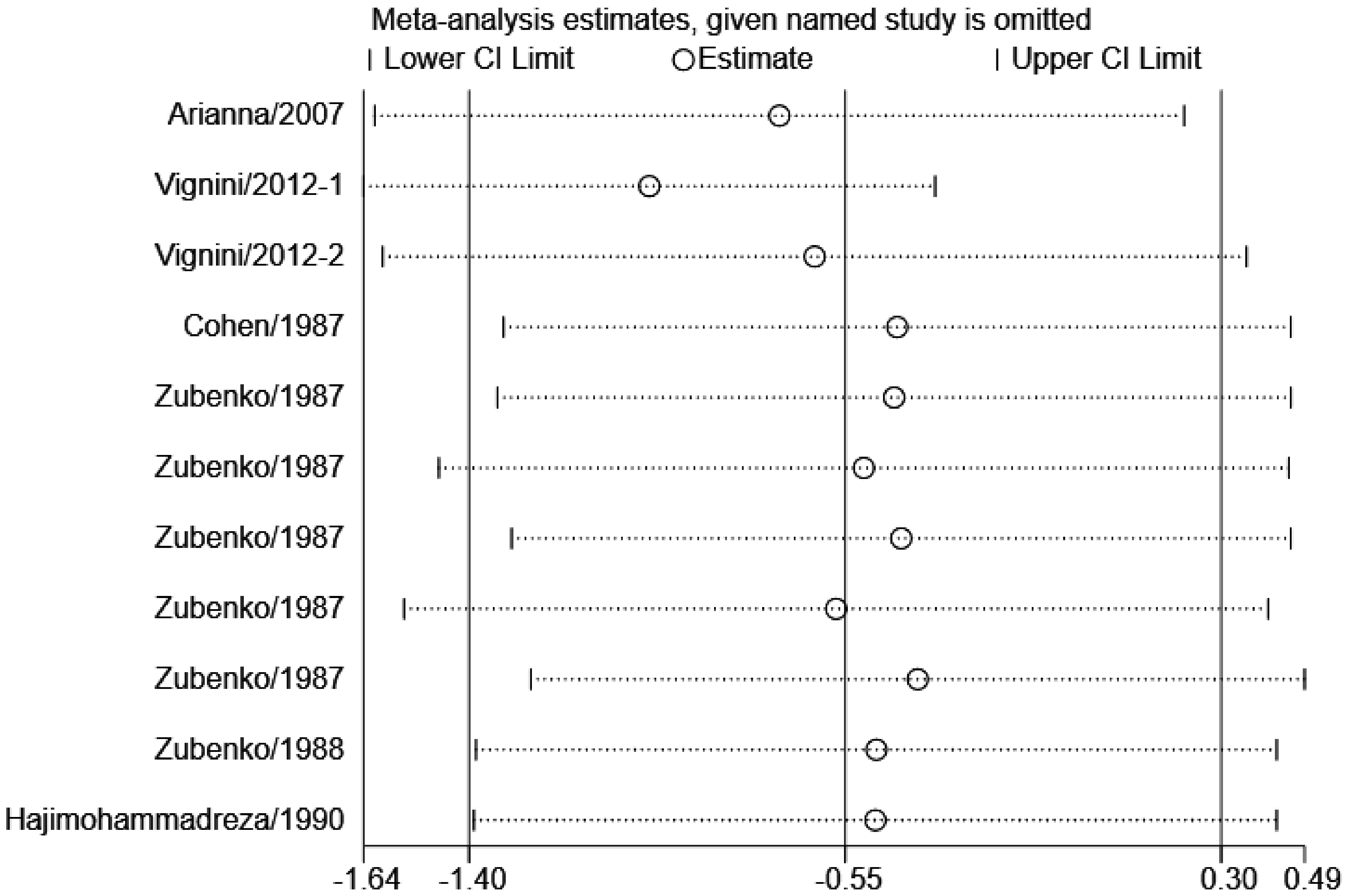

Figure S58: Sensitivity analysis for DPH(Steady-State Anisotropy)

Egger’s test: P＞0.05

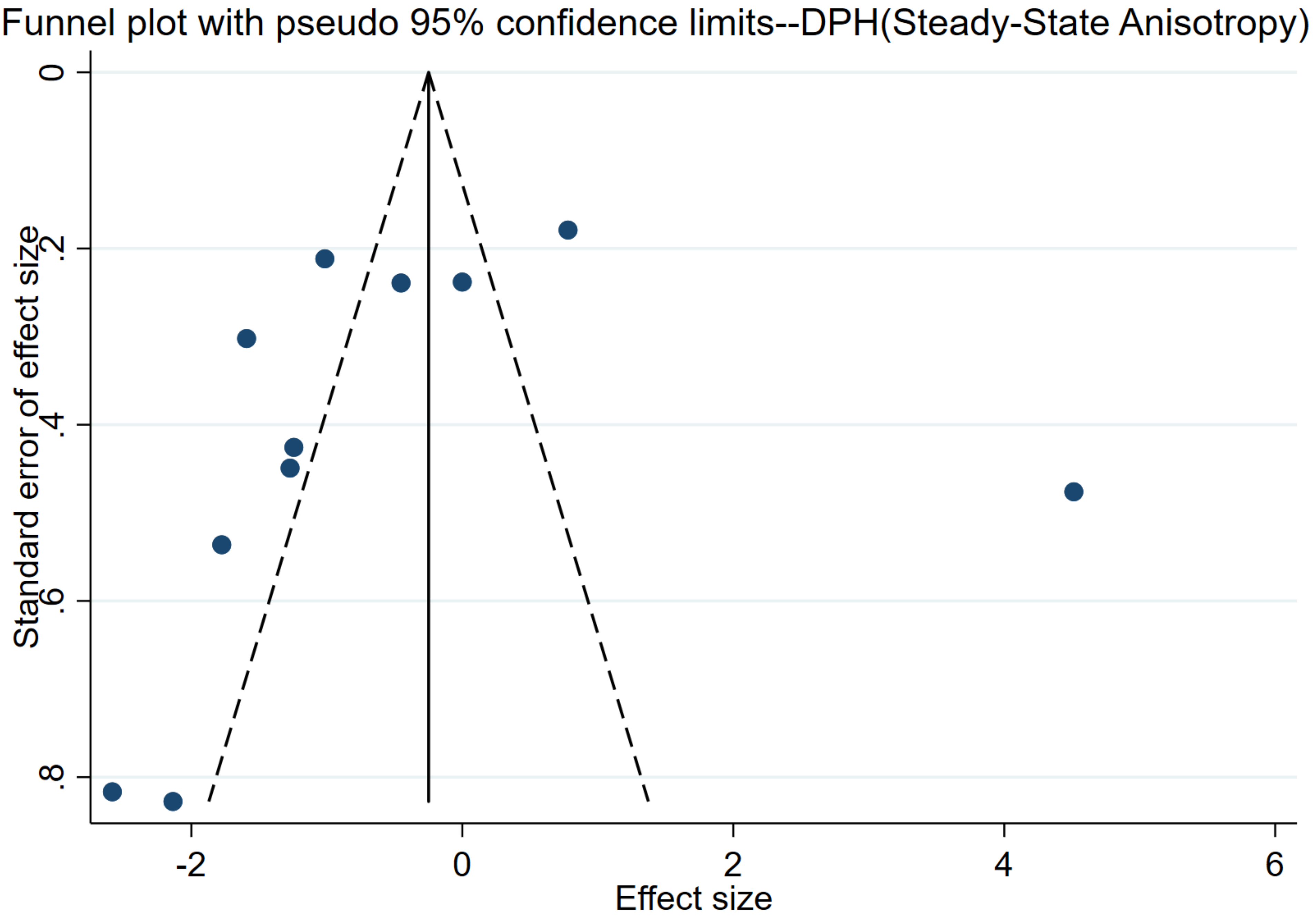

Figure S59: funnel plot of DPH(Steady-State Anisotropy); Egger's test: P > 0.05

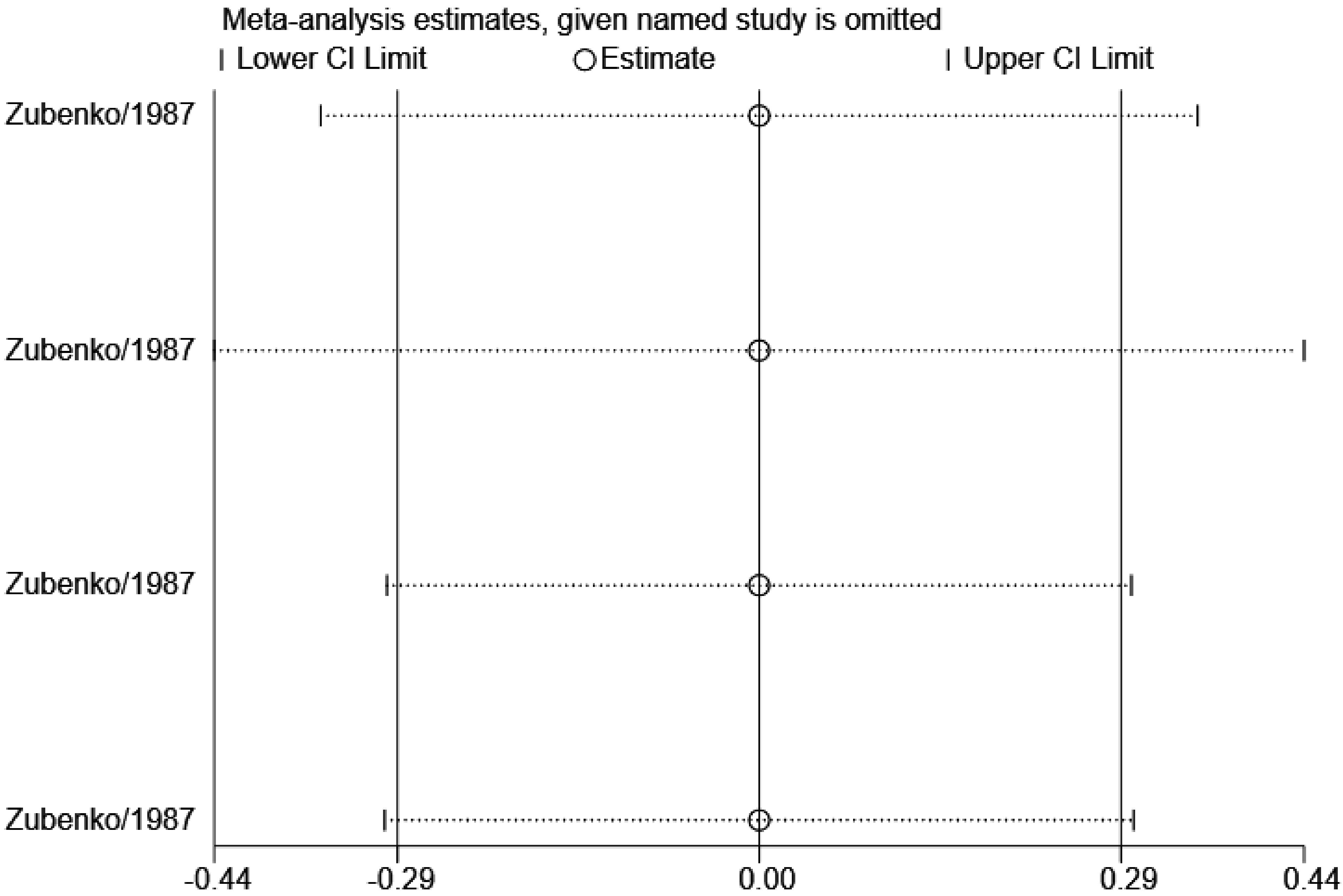

Figure S60: Sensitivity analysis for DPH(Fluorescence Lifetime)

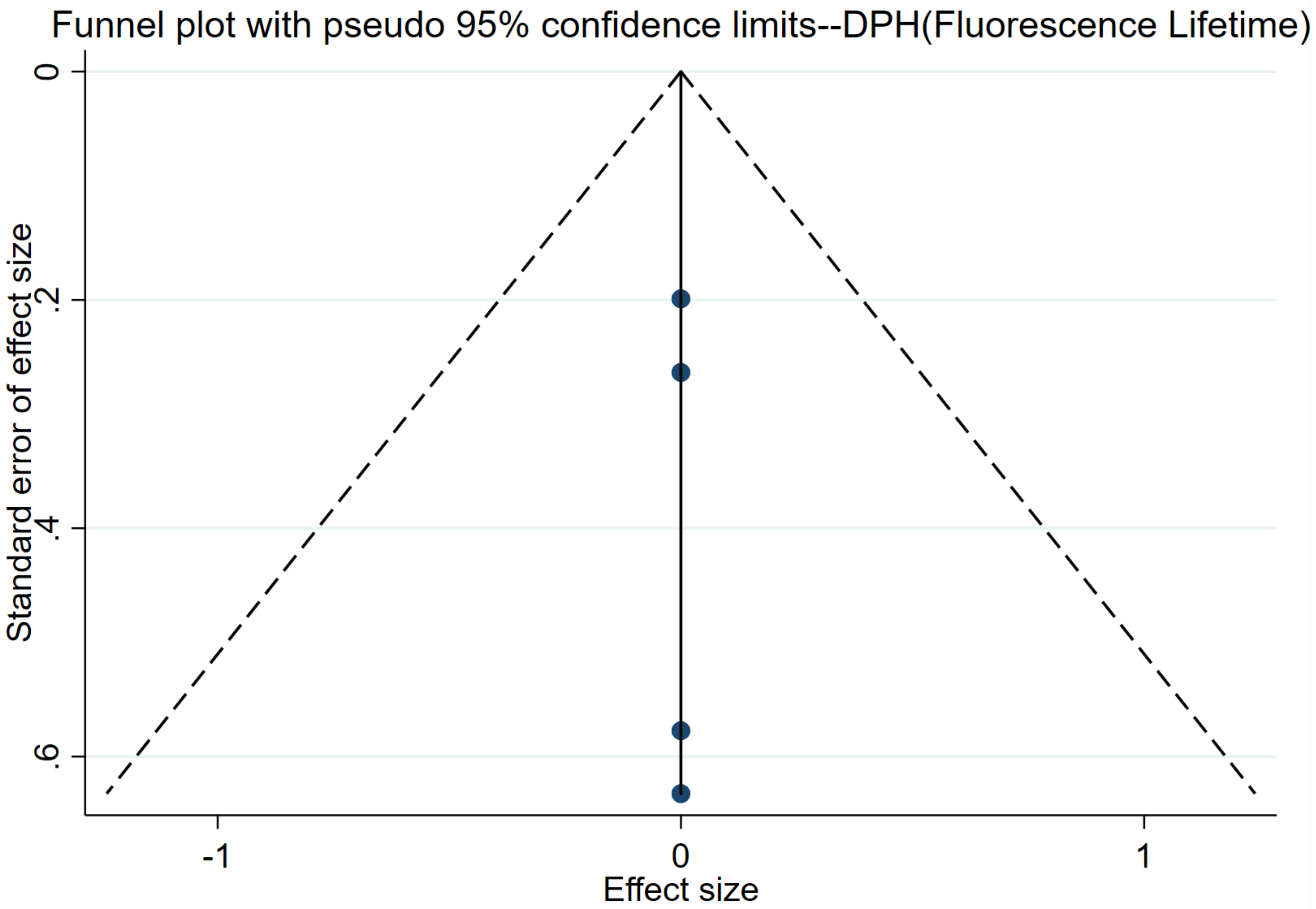

Figure S61: funnel plot of DPH(Fluorescence Lifetime); Egger's test: P > 0.05

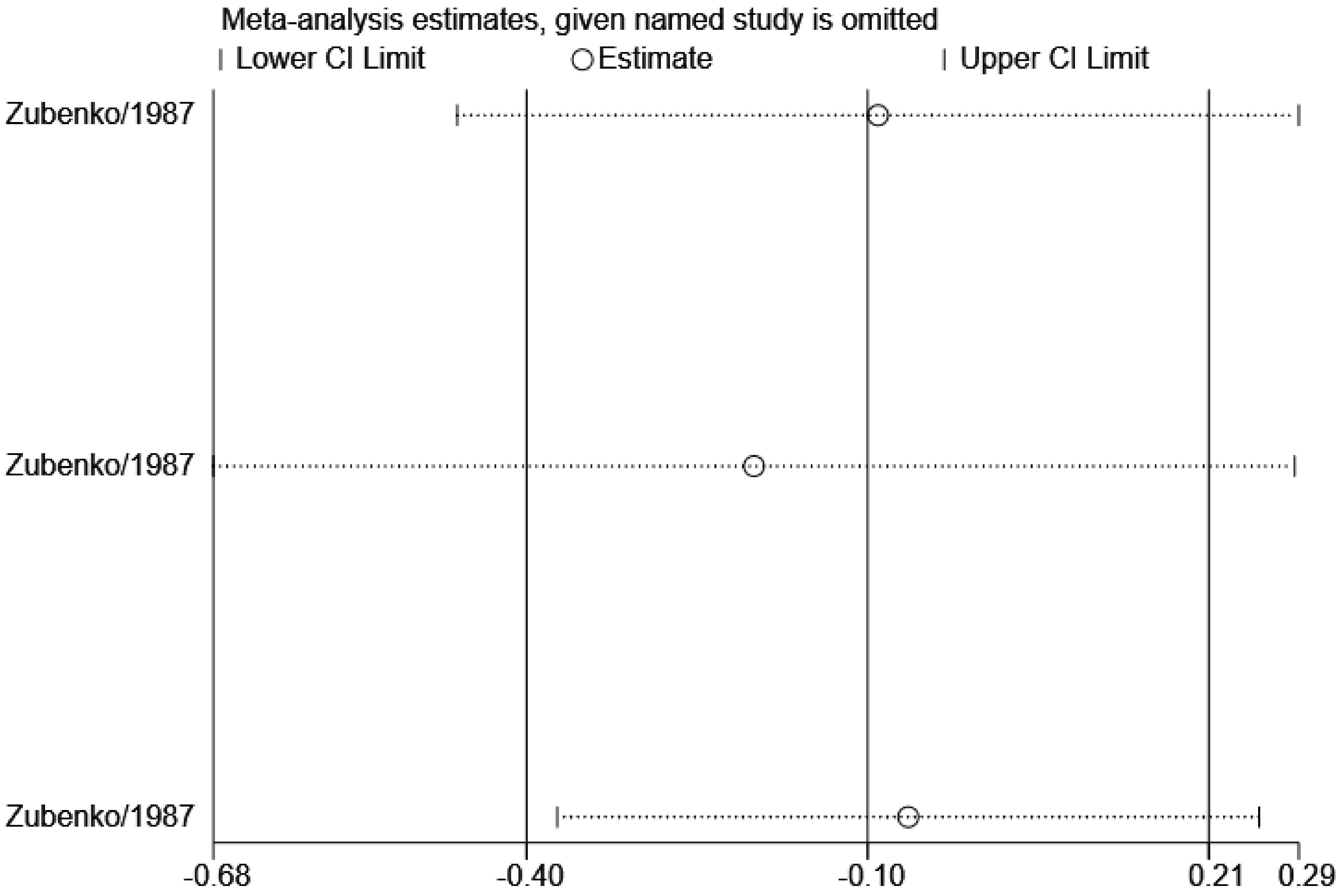

Figure S62: Sensitivity analysis for TMA-DPH(Steady-State Anisotropy)

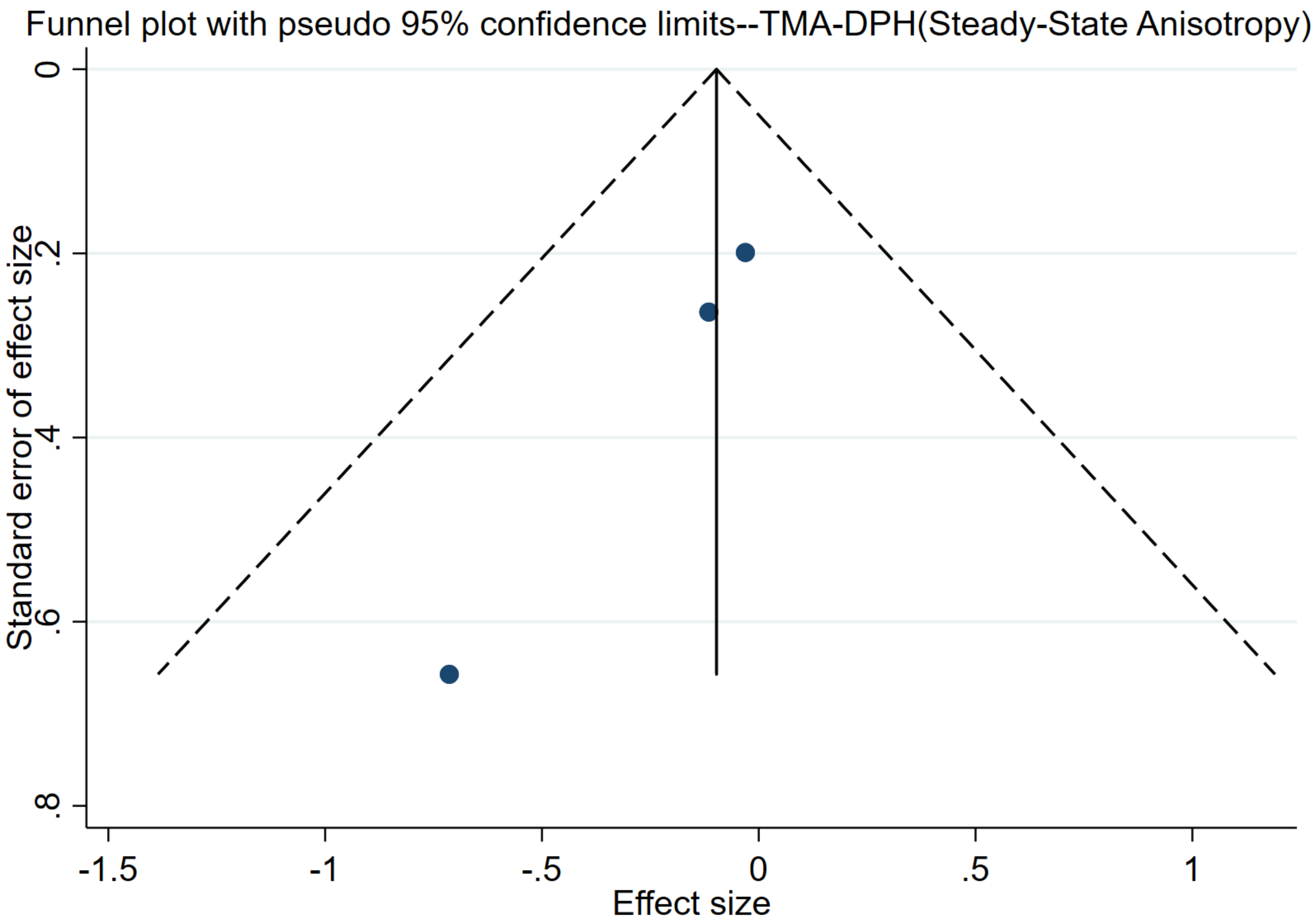

Figure S63: funnel plot of TMA-DPH(Steady-State Anisotropy); Egger's test: P > 0.05

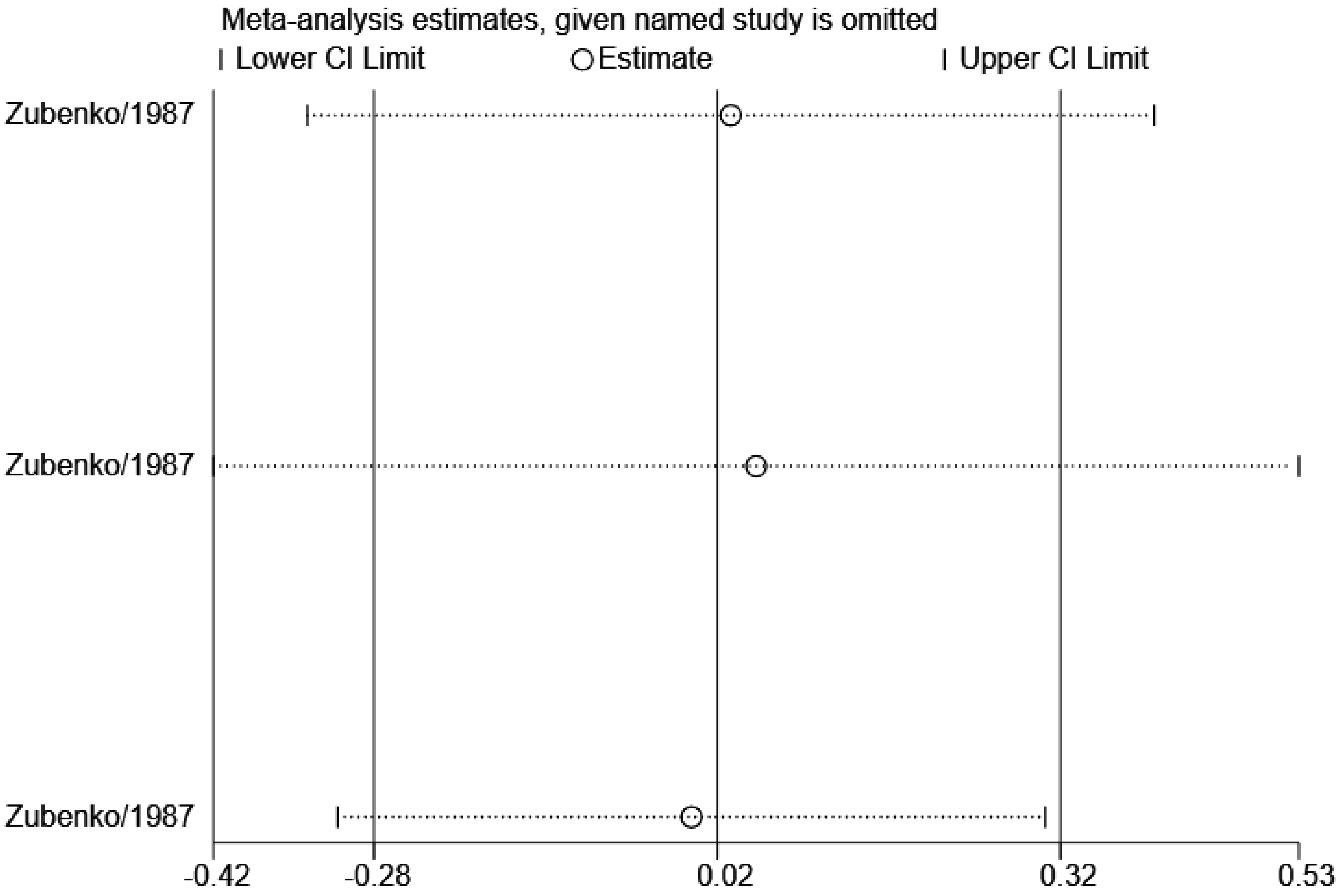

Figure S64: Sensitivity analysis for TMA- DPH(Fluorescence Lifetime)

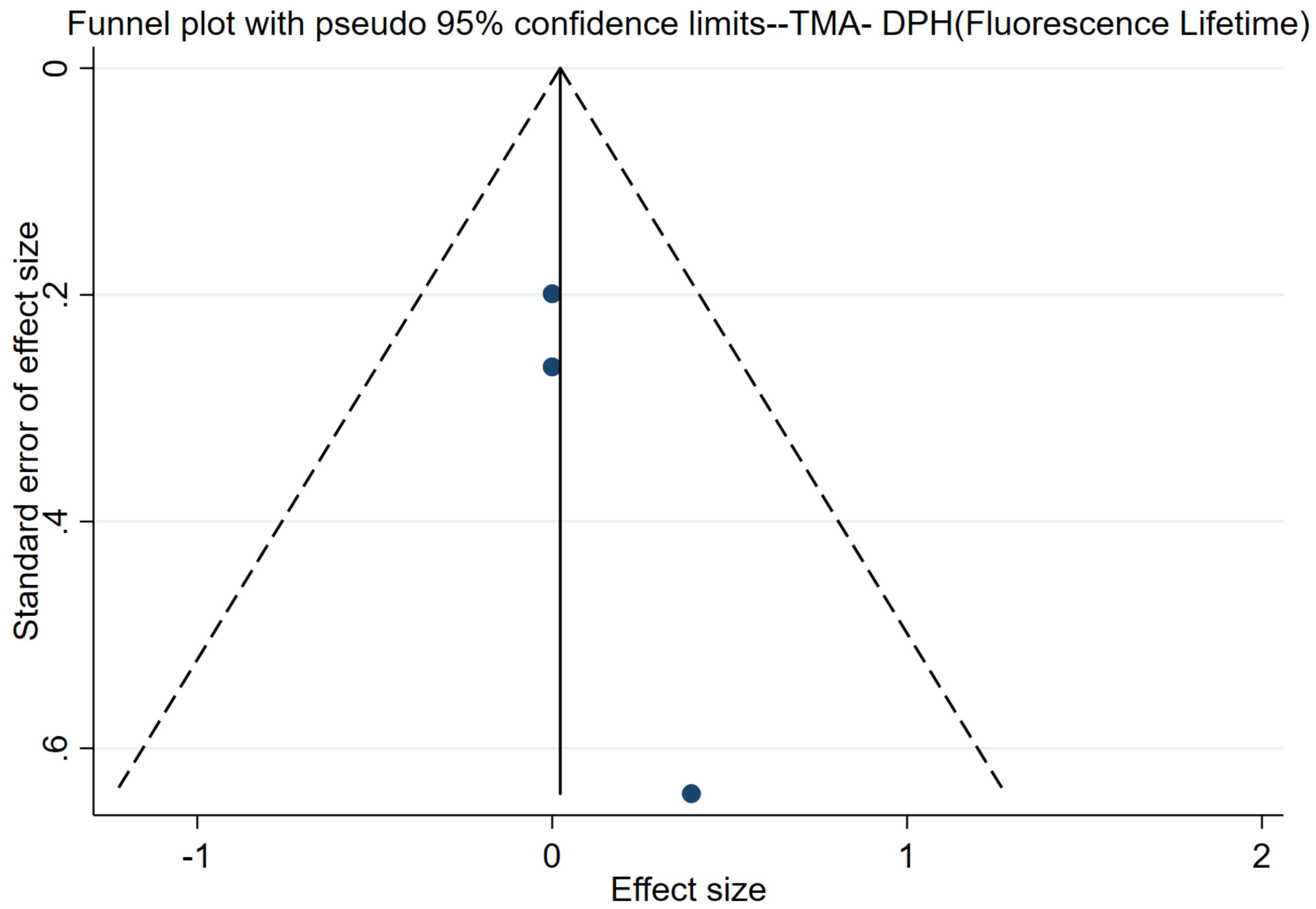

Figure S65: funnel plot of TMA- DPH(Fluorescence Lifetime); Egger’s test: P > 0.05
